# Development and application of a geospatial index of urban playability for young children

**DOI:** 10.1016/j.cities.2025.106642

**Published:** 2026-03

**Authors:** Emily Gemmell, Alicia Cavenaugh, Mariana Brussoni, Martin Guhn, Federico Andrade-Rivas, Ismam Imon, Michael Brauer

**Affiliations:** a School of Population and Public Health, University of British Columbia, 2206 West Mall, Vancouver, BC, V6T 1Z4, Canada; b Department of Geography, McGill University, 805 Sherbrooke Street West, Montreal, Quebec, H3A 0B9, Canada; c Human Early Learning Partnership, University of British Columbia, 2206 West Mall, Vancouver, BC, V6T 1Z4, Canada; d Department of Pediatrics, Rm 2D19, 4480 Oak Street, BC Children’s Hospital, Vancouver, BC, V6H 3V4, Canada; e British Columbia Children’s Hospital Research Institute, 938 West 28th Avenue, Vancouver, BC, V5Z 4H4, Canada; f School of Public Health and Social Policy, University of Victoria, HSD Building, room B202, Victoria, BC, V8N4V3, Canada; g Instituto de Salud y Ambiente, Universidad El Bosque, Av. Cra 9 No. 131 A - 02, Edificio Fundadores, Bogotá, Colombia; h Institute for Health Metrics and Evaluation, University of Washington, Hans Rosling Center for Population Health, 3980 15th Ave NE, Seattle, WA, 98195, USA

**Keywords:** Child health, Outdoor play, Urban health, Health equity, Child development, Physical activity

## Abstract

Playing outdoors supports young children’s physical, cognitive and social-emotional health and development. However, urban environments may limit children’s outdoor play. We developed an evidence-based index to evaluate neighbourhood supportiveness for young children’s outdoor free play, and applied it across 35 Canadian cities.

From an evidence-based, theoretical framework for neighbourhood playability among children, 2–6 years, we identified five major domains influencing outdoor free play: *spaces for play, social, traffic/pedestrian* and *natural environments*, and *child-relevant destinations*. We selected indicators for each domain from open-source geospatial, satellite and census data, and weighted indicators based on findings from a survey of experts. We applied the index at the postal code level, and examined associations between playability, population density and material advantage/disadvantage.

We found wide variation (52–77 %) in neighbourhood playability within the same city. However, average playability differences between cities was relatively small (≤ 20 %). Higher density areas had higher traffic/pedestrian and child-relevant destination scores, but lower social and natural environment scores, while space for play showed no relationship with density (persons/km^2^). Within study cities, 39 % of young children lived in neighbourhoods where at least one domain averaged at or below the 10th percentile score. For a majority of cities (20/35), materially disadvantaged neighbourhoods had lower playability scores.

Across Canadian cities, children’s access to playable neighbourhoods varies widely. The playability index enables small area-level assessment of supportiveness, barriers and facilitators to young children’s outdoor play. The theoretical framework and methodological approach may be adapted to develop indices of playability across diverse urban contexts.

Increasing evidence points to the importance of outdoor free play for young children’s physical, social and emotional health, development and wellbeing (Gray et al., 2015; Herrington & Brussoni, 2015; Sääkslahti & Niemistö, 2021; Whitebread & O’Sullivan, 2012). By 2050, 70 % of human populations are expected to reside in cities (United Nations, 2022), with everyday opportunities, experiences and exposures in early childhood influenced by the form and quality of urban environments. However, urban physical and functional characteristics largely reflect adult activity patterns and priorities (Freeman & Tranter, 2012). Children’s outdoor play has decreased over time, coincident with lower physical activity and higher rates of childhood obesity and poor mental health (Abarca-Gómez et al., 2017; Gray, 2011; Gray et al., 2015). Measures to assess urban environmental supportiveness for health have often focused on adult behaviours and mobility patterns, with existing measures such as walkability found to be inconsistently, or even negatively associated with children’s outdoor play (Janssen & King, 2015; Loptson et al., 2012). This research aimed to develop and apply an evidence-based measure of urban neighbourhood supportiveness for outdoor free play in young children (2–6 years, inclusive), and to investigate children’s equitable access to playable environments across 35 Canadian cities.

The concept of playability, “…how friendly environments are for outdoor play and independent mobility” (Han et al., 2018) centers on factors that make a place more or less conducive to children’s engagement in outdoor play. Though it may be associated with more play, playability is not a measure of play itself. Interactions between neighbourhood characteristics (e.g. land use, transportation infrastructure, greenspace), local social environments and family and child characteristics influence children’s engagement in outdoor free play (Gemmell et al., 2022). Time and freedom to play are often related to wider societal trends and values that influence parenting norms, child time-use and behaviour (Mullan, 2019). Multi-scale, multi-level and interacting drivers of human behaviour, wide variation in populations and settings, and self-selection into neighbourhoods pose challenges to validity and generalizability in studies of neighbourhoods and behaviour (Lamb et al., 2020). Because of this complexity, our work focused on understanding and measuring neighbourhood features shown to influence children’s outdoor free play across diverse urban and cultural contexts (Gemmell et al., 2022), applying this general framework to develop an index of urban playability for Canadian cities. Though varying widely, Canadian cities have, on average, higher population densities (Moore, 2014), mixed-use development (Pucher & Buehler, 2006) and public transit use (Moore, 2014) than U.S. cities, lower density and mixed-use development compared to European cities, and are likely to be less dense and compact than major cities in developing nations (Huang et al., 2007). Despite planned development, more available greenspace and safer traffic/pedestrian infrastructure compared to many urban settings, worldwide (Gemmell et al., 2023), children’s outdoor free play in Canadian cities is still limited by interacting characteristics of physical and social environments (Gerlach et al., 2019; Vlaar et al., 2019).

In this work, we focus on the playability of urban environments for 2–6 year-old children (inclusive), an age range that encompasses the start of semi-independent experiences of outdoor environments, and early developmental stages when experiences, behaviours and exposures are particularly impactful for lifelong health and wellbeing (Wyver, 2024; Herman et al., 2009; Telama et al., 2014). Young children are excluded from active use of a large proportion of public outdoor space in cities (roadways), due to traffic danger, and play in other public outdoor spaces may be limited by parental perceptions of social danger and parental time constraints (Cronin-de-Chavez et al., 2019; Penilla et al., 2017). Young children have slower walking (Cavagna et al., 1983; Vaughan & Bain, 1999) and cycling speeds (Crump & Bui, 2019), making proximity to spaces for play of great importance (Flowers et al., 2019). Young children view nearly any space as potentially playable (Gemmell et al., 2022), and small informal spaces and affordances (e.g. bike racks, planters, bushes, puddles) near home or along routes may be as important as formal play destinations (Ergler et al., 2021). In urban contexts, young children usually require adult accompaniment to access outdoor spaces, thus, adult time, motivation and enjoyment of the space are also important determinants of outdoor free play (Gemmell et al., 2022). Adult-directed, structured and indoor activities take up an increasing share of children’s time, and families who cannot afford these may also lack access to private space for outdoor play (Chawla, 2016). As freedom to engage with local physical and social environments has declined, children’s engagement with virtual worlds has expanded, with evidence increasing for negative impacts on early development (Kerai et al., 2022).

In this paper we operationalize a previously developed, evidence-based, theoretical framework for urban playability (Gemmell et al., 2022), using census and open source geospatial and satellite data, to create a scalable, high-resolution measure of neighbourhood playability for young children. We applied this metric at the postal-code level across major Canadian cities and explored relationships between playability, material advantage and population density within and across cities.

## 1 Methods

**Fig. 1:**
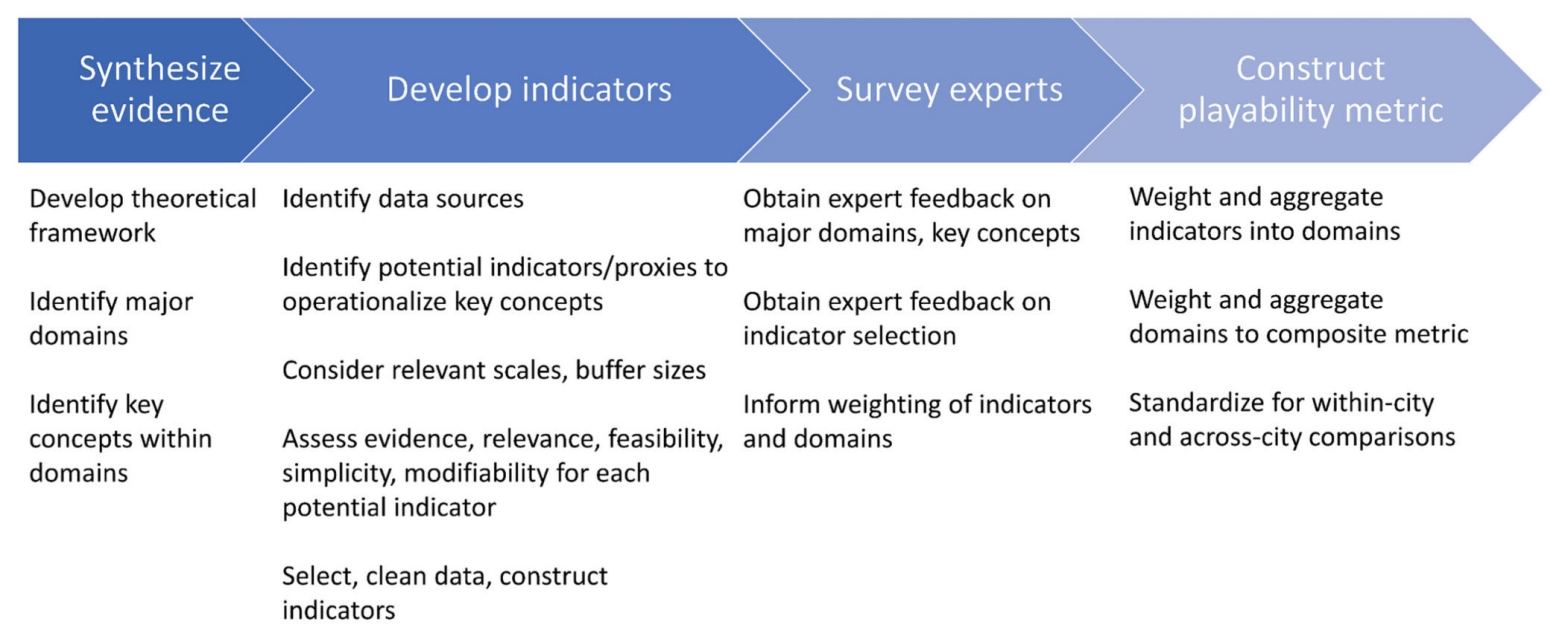
Major steps in the development of an urban playability metric.

Our approach to metric development consisted of four major steps: synthesis of evidence and identification of major neighbourhood-level influences on playability, selection of indicators to operationalize distinct concepts within these domains, a survey of experts to inform weighting of metric components and obtain feedback on indicator selection and finally, weighting and aggregation of indicators and domains into a composite metric. The process illustrated in Fig. 1 is described in detail below.

**Fig. 2:**
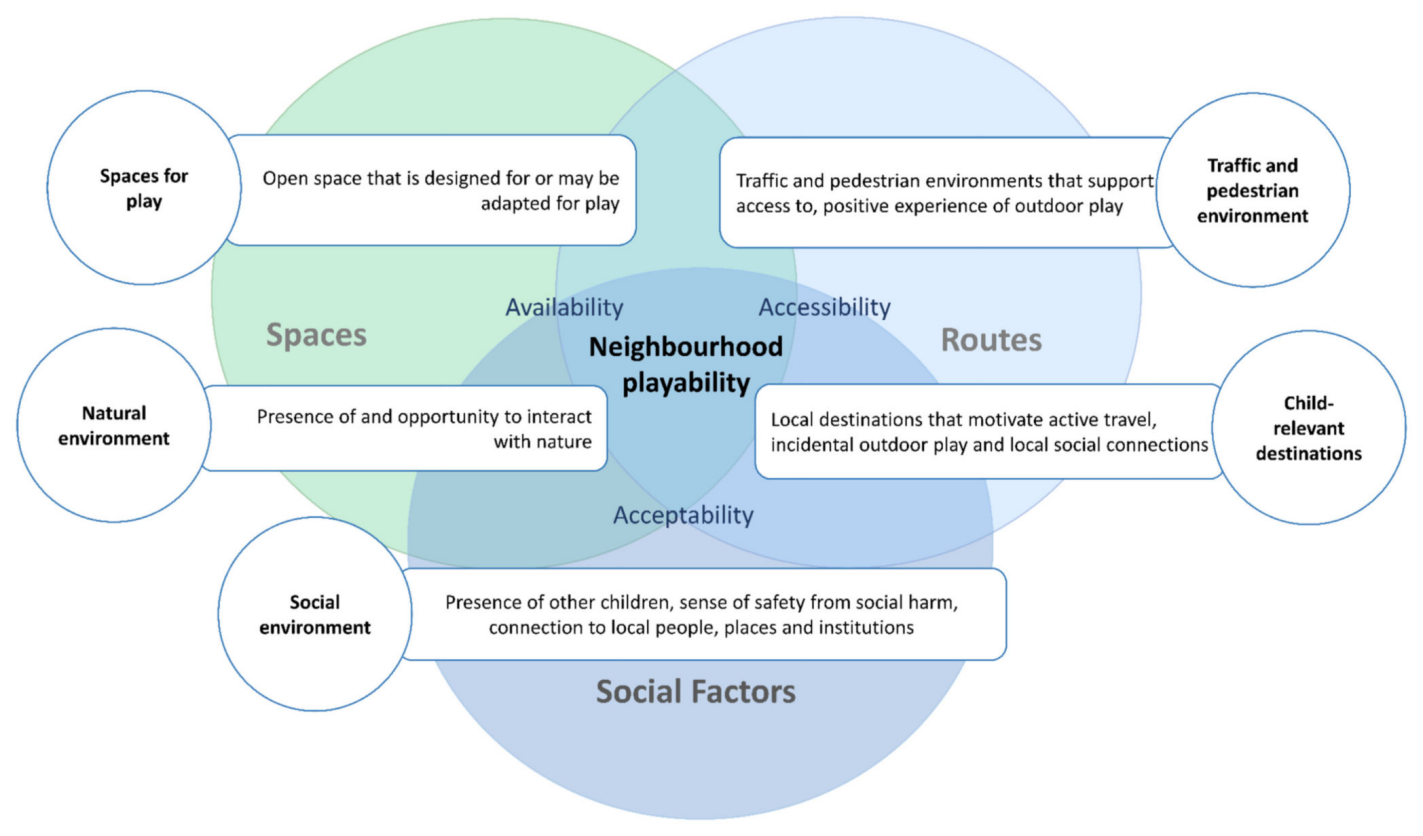
What makes an urban neighbourhood playable? Major domains influencing the availability, accessibility and acceptability of neighbourhoods for young children’s outdoor free play. (adapted from ‘Influence of neighbourhood built environments on the outdoor free play of young children: a systematic, mixed-studies review and thematic synthesis’ by E. Gemmell et al., Journal of Urban Health, 100(1), 142. Copyright 2022 by The New York Academy of Medicine. Used with permission.).

Previously, we conducted a systematic, mixed studies review and thematically synthesized evidence for built environment influences on outdoor free play in young children (Gemmell et al., 2022), developing a simple, theoretical framework to conceptualize neighbourhood influences on young children’s outdoor free play. Intersections of the three broad themes identified in the review: *spaces for play, routes, and social factors*, influenced the availability, accessibility and acceptability of neighbourhood environments for young children’s outdoor free play (Gemmell et al., 2022). To develop the composite playability metric, we thematically grouped specific neighbourhood features identified through the systematic review into five major domains: spaces for play, traffic/pedestrian environment, natural environment, social environment and child relevant destinations. Features within these five major domains arise from intersecting characteristics of spaces, routes and social factors within the neighbourhood (Fig. 2).

### 1.1 Study settings

We selected the 35 largest census metropolitan areas (CMAs) in Canada as the settings for this research. CMAs in Canada are large population centers (>100,000 population) usually composed of multiple smaller municipalities that form continuous urban/suburban areas (Government of Canada, S. C., 2017). In Canadian urban areas, six-digit postal codes are assigned to areas roughly one side of a city block, providing relatively accurate location point estimates for measurement of spatially correlated exposures in cities (Pinault et al., 2020). For indicators for which postal code-level data was not available, we used dissemination area (DA) level data. Dissemination areas (DAs) are the smallest standard geographic area for which census data are disseminated, and are relatively stable geographic units with an average population of 400 to 700 persons (Statistics Canada, 2021a).

### 1.2 Development of playability indicators

Potential data sources for operationalizing distinct concepts within the five major playability domains were identified from data available across study cities. Indicators were selected based on a qualitative evaluation of the conceptual alignment of the indicator with the underlying concept, the feasibility, usefulness and strength of evidence for each potential indicator (Alberta Health Services, 2011). We considered the mechanisms by which indicators influence outdoor free play, and the scales at which they are likely to have the strongest influence for young children, selecting buffer types and sizes for each indicator accordingly. Circular buffers were used when the entire area surrounding the residence may influence a child’s experience or exposure (e.g., building density, neighbourhood greenness, tree canopy, road types around residence). Network buffers were used to measure exposures that are encountered primarily via mobility through road or path networks (e.g. child relevant destinations, intersections). In selecting buffer sizes, we considered walking and cycling speeds for young children. Walking speed for 2–6 year-old children ranges between 2.8 and 4.6 km/h (Cavagna et al., 1983), and cycling speed average for 4–5 year-old children is 6.5 km/h (Crump & Bui, 2019; Vaughan & Bain, 1999).

We identified all postal codes within the study cities using DMTI Spatial CanMap Content Suite 2020 v.3 and DMTI Postal Code Suite 2020 v.3 (DMTI Spatial Inc, 2021a; DMTI Spatial Inc, 2021b) Below we briefly summarize the rationale for selection, data sources and the general methods used in individual indicator development. (Detailed information on indicator selection is available in Supplementary files, Tables A.1-A.8.) Wherever possible, we used open access data to facilitate replication and adaptation of our methods to other contexts. Data cleaning, indicator development and analysis was carried out using R statistical computing language unless otherwise specified (R Core Team, 2022).

#### 1.2.1 Spaces for play

Presence of neighbourhood open space that is designed for, or may be adapted for outdoor free play was operationalized by two indicators representing formal and informal spaces for play. Neighbourhood formal spaces for play were measured by calculating the number of parks, playgrounds and other recreation areas from the CanMap Content Suite 2020 v.3 and OpenStreetMap datasets, within a 500 m circular buffer of each postal code centroid using the sf package (OpenStreetMap contributors, n.d.; DMTI Spatial Inc, 2020). There is a strong conceptual link between neighbourhood open space and outdoor free play, supported by both qualitative (Abu-Ghazzeh, 1998; Ergler et al., 2021; Phillips, 2016) and quantitative evidence (Islam et al., 2016). Availability of yard space has been linked to children’s outdoor play (Marino et al., 2012) and qualitative evidence suggests that children value nearby, informal spaces for play (Gemmell et al., 2022). Previous studies have utilized building footprint data to estimate open space in neighbourhoods, when data for yard or other informal spaces was lacking (Christian et al., 2017; Islam et al., 2016). Islam et al., reported a 1 min decrease in outdoor play time among 9–14 year-olds for each additional 1000m^2^ neighbourhood space covered by a building footprint (Islam et al., 2016). The Canadian Urban Environmental Research Consortium (CANUE) obtained measures of area covered by building footprints using high resolution satellite data and machine learning methods described by Setton et al (Setton & Redivo, 2021). We reverse-coded the percent area within a 250 m circular buffer of each postal code that is covered by a building footprint (Setton & Redivo, 2021) to indicate the simple availability of *neighbourhood open space that may be adapted for play*. The presence of yards, vacant lots, streets, parking lots and other *potentially* playable neighbourhood spaces (Abu-Ghazzeh, 1998; Ergler et al., 2021; Phillips, 2016), are captured by this measure. Actual adaptation and use of informal spaces for play depends not only upon space availability, but also its accessibility and acceptability to parents and children (Gemmell et al., 2022). Although it does not provide information on accessibility or quality of the space, the quality of informal spaces for play may be partially inferred by indicators within other major playability domains (e.g. natural, traffic/pedestrian, social environment indicators).

#### 1.2.2 Social environments

Knowing/trusting neighbours, establishing connection to local spaces and institutions, and presence of other children can support children’s outdoor free play (Gemmell et al., 2022; Parent et al., 2020). We utilized two variables from small area-level (DA) census data: the proportion of residents who moved from outside the municipality within the past 5 years, and the proportion who immigrated within the past 5 years, as proxies for *knowledge of, and connection to local people, places and institutions*. (The proportion who moved or immigrated *within the past year* was also considered, however, the data was not releasable due to privacy considerations.)The proportion of residents age 0–14 years (inclusive) in each DA was used to capture the *potential for social play* in local areas. We combined DAs with very small populations to meet Statistics Canada requirements for release (Statistics Canada, 2016), and linked DA-level variables to each postal code within the DA.

#### 1.2.3 Traffic and pedestrian environments

We used four geospatial variables to characterize neighbourhood traffic and pedestrian environments: road types, intersections, walking routes and cycling routes. Traffic volume, type and speed are linked to child and parental perceptions of the safety and quality of neighbourhood routes to, and spaces for play (Gemmell et al., 2022; Tavakoli et al., 2024), and are associated with environmental noise (Clark et al., 2021) and air pollution (Wang et al., 2022) that may influence outdoor free play. We developed a proxy for road traffic exposure around residence by calculating the lengths of each road type within a 250 m buffer of postal code centroids, and multiplying these by a coefficient based on traffic volume, speed and vehicle type for each road classification. (Supplementary Files, Table A.4).

For young children, intersections posed barriers to reaching spaces for play (Gemmell et al., 2022). We derived intersection point data from OpenStreetMap road network data (OpenStreetMap contributors, n.d.) using QGIS 3.24.2 (QGIS Development Team, 2022),. calculating and reverse-coding the number within a 1000 m network buffer of postal code centroids using the r5r package in R (Pereira et al., 2021; R Core Team, 2022).

Sidewalk networks and paths supported young children’s outdoor free play in urban areas, enabling safe access to local destinations and facilitating incidental play along routes (Gemmell et al., 2022). We developed a proxy for walking environments by summing the lengths of paths, walking-only roads, residential roads and service roads (e.g. alleyways), over the total length of all roads within a 1000 m network buffer of postal code centroids, to represent *walking networks with low traffic exposure*.

Child-appropriate cycling networks were important to children’s outdoor free play and active mobility (Gemmell et al., 2022). Canadian Bikeway Comfort and Safety Classification System (Can-BICS) dataset provides classification of low, medium and high comfort and safety for bike routes and has applied these to OpenStreetMap cycling networks across Canada (Ferster et al., 2023). We calculated the total length of cycling routes classified as high or medium comfort and safety, over the total lengths of all roads within a 1000 m network buffer of each postal code centroid using the r5r package in R (Pereira et al., 2021).

#### 1.2.4 Natural environment indicators

Neighbourhood natural environments were characterized by three indicators: neighbourhood greenness, tree canopy and blue space. The Normalized Difference Vegetation Index (NDVI), a measure derived from remote-sensing (satellite) imagery, has been used widely in environmental health studies to assess exposure to green space (Labib et al., 2020). Higher NDVI (scale: −1 to +1) was associated with children’s outdoor play in some studies in our systematic review, and perceived lack of green space was reported as a barrier to outdoor free play (Gemmell et al., 2022). The annual average NDVI within 250 m circular buffer around each postal code was obtained from the CANUE database. NDVI was derived by CANUE from Top of Atmosphere reflectance data in bands from cloud free annual composites of USGS Landsat 5 and Landsat 8 satellite images (United States Geological Surveyd, n.d.; United States Geological Surveyc, n.d.; United States Geological Surveya, n.d.; DMTI Spatial Inc, 2015; Gorelick et al., 2017; United States Geological Surveyb, 2013).

Qualitative evidence indicates that trees are highly valued by children and parents, supporting outdoor free play across contexts (Gemmell et al., 2022). Tree canopy (area of vegetation of woody plants with height above 5 m) within a 250 m circular buffer around each postal code centroid was obtained for 34 of the 35 study areas (CANUE, n.d.). These estimates were derived from the Global Forest Cover Change (GFCC) Surface reflectance product, based on high-resolution Landsat 5 Thematic Mapper and Landsat 7 Enhanced Thematic Mapper Plus images (Gorelick et al., 2017; Markham, 2010; Sexton et al., 2013). For St. John’s census metropolitan area, no tree canopy data was available from the GFCC for the years represented. To obtain estimates for St. John’s we downloaded GFCC tree canopy data for 2005 from United States Geological Survey (USGS) Earth Data, and calculated the percent tree canopy coverage within 250 m of each postal code location (Townsend, n.d.; Markham, 2010).

Young children demonstrated affinity for water and blue spaces for outdoor free play in qualitative studies (Gemmell et al., 2022). We calculated the proportion covered by waterways, streams, rivers, lakes and oceans within a 1000 m circular buffer of postal code centroids from the CanMap Content Suite 2020 v.3 (DMTI Spatial Inc, 2021a).

#### 1.2.5 Child-relevant destinations

Incidental play often occurs on the way to local destinations and local active travel supports young children’s social and place connections (Gemmell et al., 2022). We obtained data for school, community and recreational facility locations from OpenStreetMap (OpenStreetMap contributors, n.d.), Statistics Canada’s Open Database of Educational Facilities (ODEF) (Statistics Canada, 2021b), and Open Database of Sports Facilities (ODSF) (Statistics Canada, 2021c), and summed the number of each type within a 1000 m network buffer distance from postal code centroids.

### 1.3 Expert survey

We conducted an online survey of researchers and professionals working at the intersections of children’s outdoor play and urban planning, child health and development, child physical activity, injury prevention, children’s rights and environmental health, to inform weighting of domains and indicators (Organisation for Economic Co-Operation and Development (OECD), 2008), and to elicit feedback on proposed indicators. Invitees were identified as authors on peer-reviewed publications related to children’s outdoor play, with efforts made to maximize the diversity of research location and interdisciplinary make-up of the group. Emailed invitations were sent to 26 potential participants with information on the survey and a link for participation. Using the budget allocation method (Organisation for Economic Co-Operation and Development (OECD), 2008), participants assigned points (out of 100 total points) to each of the five major playability domains, based on their assessment of its relative importance to outdoor play for two child age groups; 0 through 6, and 7 through 12 years, inclusive. Experts also assigned points to potential indicators (out of 100 per domain) to indicate the relative importance of each indicator within a domain. (Full survey is available in Supplementary file, Table B.1).

To assess the reliability of the average expert weight rating, we calculated the intra-class correlation coefficient (ICC (Gray et al., 2015; Herrington & Brussoni, 2015) for a two-way, mixed effects model (domains and indicators as random effects, participants as fixed effects) using the *irr* package in R (R Core Team, 2022; Trevethan, 2017). We compared the weights for early childhood and middle childhood age groups, using the Wilcoxon signed rank test for paired samples. We assigned weights to each playability domain, and to individual indicators within a domain, using the expert rating point averages for the 0 through 6 year-old age group. Comments on the overall selection of playability indicators were also provided by participants and considered in indicator selection, operationalization and sensitivity analyses. A detailed description of changes made based on qualitative comments are provided in Supplementary file, Table B.4.

### 1.4 Development of a composite urban playability metric

Because within-city differences at the neighbourhood level are likely to be of greatest relevance to municipalities, urban planners and the public, we developed *within-city* playability scores; normalizing, scaling, weighting and aggregating indicators separately for each city. To enable comparative descriptions across study cities we also calculated *across-city* playability scores, normalizing, scaling, weighting and aggregating indicators using data from across all 35 cities. For both within and across-city score development, we used the following general steps.

1. We normalized data for each indicator (*I*) to a z-score using the equation
  I=(I−mean(I))/sd(I)
  and scaled indicator data to between 0.0001 and 10 (non-zero lower limit avoids issues during weighting and aggregation).
2. We weighted and aggregated indicators into five playability domains, using additive aggregation (Organisation for Economic Co-Operation and Development (OECD), 2008; Gan et al., 2017) calculating domain scores (*ds*) as the weighted arithmetic mean of all indicators within the domain, using the equation
  $$\text{ds}=\omega_{1}{\text{I}}_{1}+\omega_{2}{\text{I}}_{2}\ldots +\omega_{\text{i}}{\text{I}}_{\text{i}}$$
  where *ω*_*i*_ is the weight of the *i*th indicator, and *I*_*i*_ is the normalized, scaled score of the *i*th indicator. (Gan et al., 2017) We then scaled domain scores to between 0.0001 and 10.
3. We weighted domains according to expert survey weighting point averages, and combined the five domains into a composite playability metric with additive aggregation (Gan et al., 2017), using the equation
  $$\text{ps}={\text{ds}}_{1}\omega_{1}+{\text{ds}}_{2}\omega_{2}\ldots +{\text{ds}}_{\text{i}}\omega_{\text{i}}$$
  where *ω*_*i*_ is the weight of the *i*th domain, and *ds*_*i*_ is the scaled score of the *i*th domain. We then scaled the composite playability score to a value between 0.0001 and 10.

Detailed methods and complete code for indicator, domain and metric development are available at https://github.com/egemmell/Playability-Metric-Development/tree/main.

### 1.5 Variables for descriptive and sensitivity analyses

To assess the relationship between playability and neighbourhood material advantage, we reverse-coded the Material Deprivation Index (MDI), and scaled to 0.001–10. The MDI is one component of the Material and Social Deprivation Index (MSDI), a DA-level measure developed through principal component analysis (PCA) of Statistics Canada 2016 census data to assess small-area level disadvantage (Pampelon, 2023). Indicators in the composite MDI score include proportion of individuals without a high-school diploma, the employment–population ratio, and average personal income. Physical and social features of neighbourhoods are often closely related to population density. We examined the relationships between average playability and domain scores, and population density (persons/km^2^) at the DA level using Statistics Canada 2021 census data (Government of Canada, S. C, 2022).

Data that captures the complex and multi-level social factors influencing outdoor free play in local neighbourhoods was difficult to find across study cities. We re-calculated playability scores using the Social Deprivation Index (SDI) component of the MSDI as an alternate measure of the social environment (Pampelon, 2023), and repeated descriptive analyses with complete data for 92.11 % of postal codes in study cities (Details on this measure are available in Supplementary file, Table A.7).

## 2 Results

### 2.1 Expert survey

Of 26 invited experts, 20 participated in the online survey (77 % response rate). Of these, 14 (60 %) conducted their professional work or research in North America, 3 (15 %) in Australia, 4 (17 %) in Europe, 1 (5 %) in Asia, and 1 (5 %) gave no location. Participants self-identified as academics, researchers, child rights advocates, urban planners, architects, educators, consultants, recreation or health professionals. We found high agreement across expert ratings on the relative importance of five playability domains to children’s outdoor free play, with intraclass correlation coefficient (ICC) of 0.96 (95 % CI:0.94–0.98). On average, experts considered spaces for play (mean weight 0.26) and social environments (mean weight 0.25) domains to be most influential on outdoor free play for younger children (2–6 years, inclusive) and assigned equal importance to traffic and natural environments (mean weights 0.17), and lowest importance to child-relevant destinations (mean weight 0.15). (All expert assigned weights are available in Supplementary file, Tables B.2–3).

Operationalization of major and sub-themes into domains and their component geospatial indicators is summarized in Table 1.

We applied the playability metric to 629,228 postal codes within the 35 largest Canadian CMAs. The study CMAs are home to 4,315,165 children under age 15, representing approximately 72 % of all children in this age group living in Canada (Government of Canada, 2022). We conducted descriptive analyses using complete case data for 96.51 % (*n =* 607,270) of all postal codes within the study areas. Of all children living in the study areas, 96.1 % (4,146,150) lived at postal codes with complete data (Missing data summary can be found in Supplementary file Fig. C.1.). Study CMAs vary widely in population (111,184 to 6,202,225 persons in Belleville-Quinte West and Toronto), land area (595.08 to 8046.99 km^2^ in Guelph and Ottawa-Gatineau) and population densities (37 to 1051 persons/km^2^, for St. John’s and Toronto, respectively). Percent of the population under age 5, and under age 15 ranged from 3.81 % and 12.71 % (Victoria) to 6.08 % and 19.10 % (Saskatoon), and percent with low income after tax ranged from 7.3 % (Oshawa) to 14.3 % (Trois-Rivieres). (Characteristics of study cities are given in Supplementary File, Table C.1.)

### 2.2 Playability across study cities

**Fig. 3:**
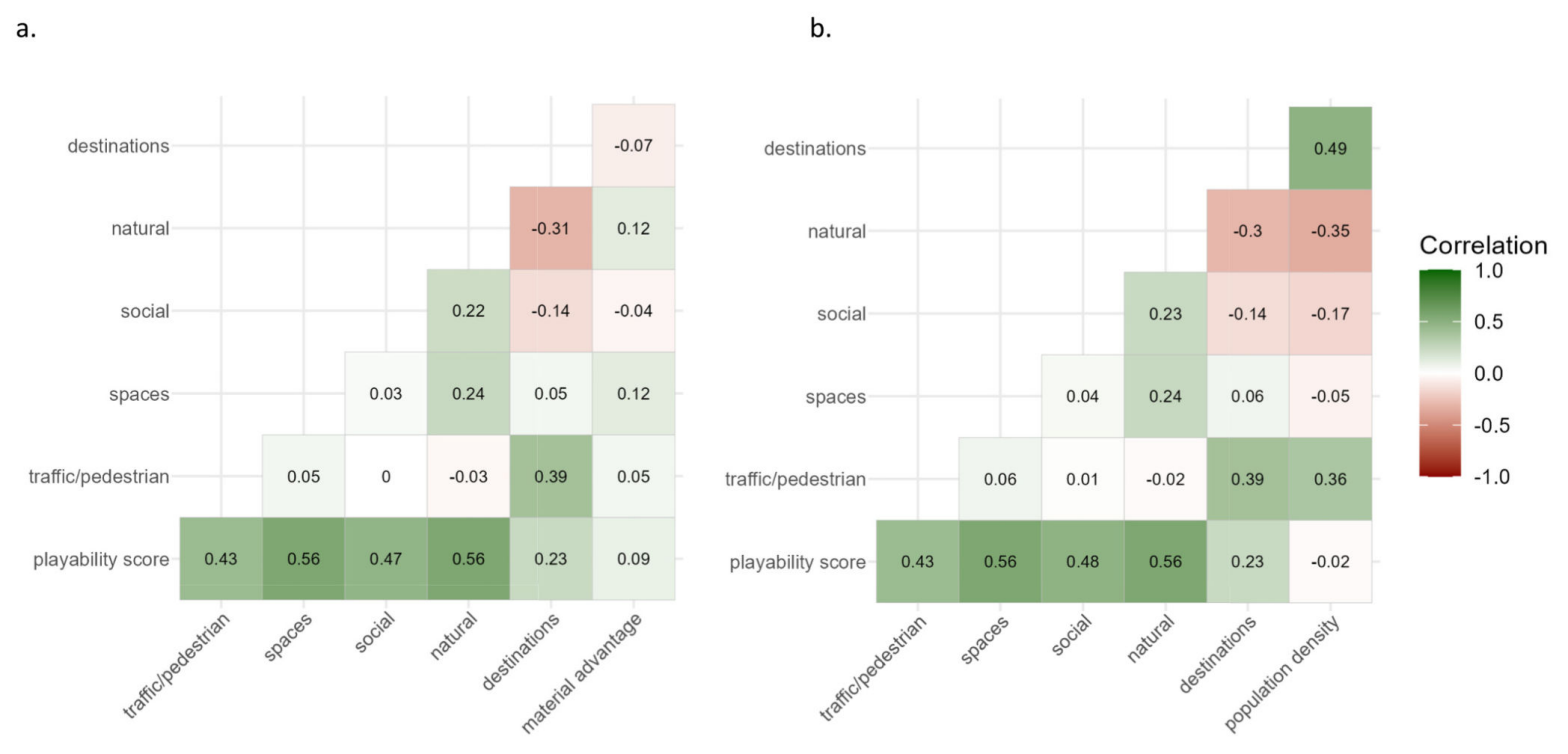
Spearman’s correlations* between playability and domain scores, and a.) small-area level material advantage, b.) small-area level population density (persons/km^2^).

Across-city playability scores (scores normalized and scaled across all study city postal codes) were positively correlated with small-area level (DA) material advantage (ρ_s_ = 0.09, *p* < 0.001); as were space for play, natural and traffic domain scores (ρ_s_ = 0.12, ρ_s_ = 0.12, ρ_s_ = 0.05, p < 0.001). However, material advantage was negatively correlated with social environment (ρ_s_ = −0.04, p < 0.001) and child relevant destination scores (ρ_s_ = −0.07; p < 0.001).(Fig. 3.a.) Population density showed a small negative correlation with across-city playability (ρ_s_ = −0.02, p < 0.001). Space for play, natural and social environment domains were negatively correlated (ρ_s_ = −0.05; ρ_s_ = −0.17; ρ_s_ = −0.35, respectively p < 0.001) and traffic environment and child relevant destinations positively correlated (ρ_s_ = 0.36, ρ_s_ = 0.49, p < 0.001) with DA-level population density. (Fig. 3.b.)

**Fig. 4:**
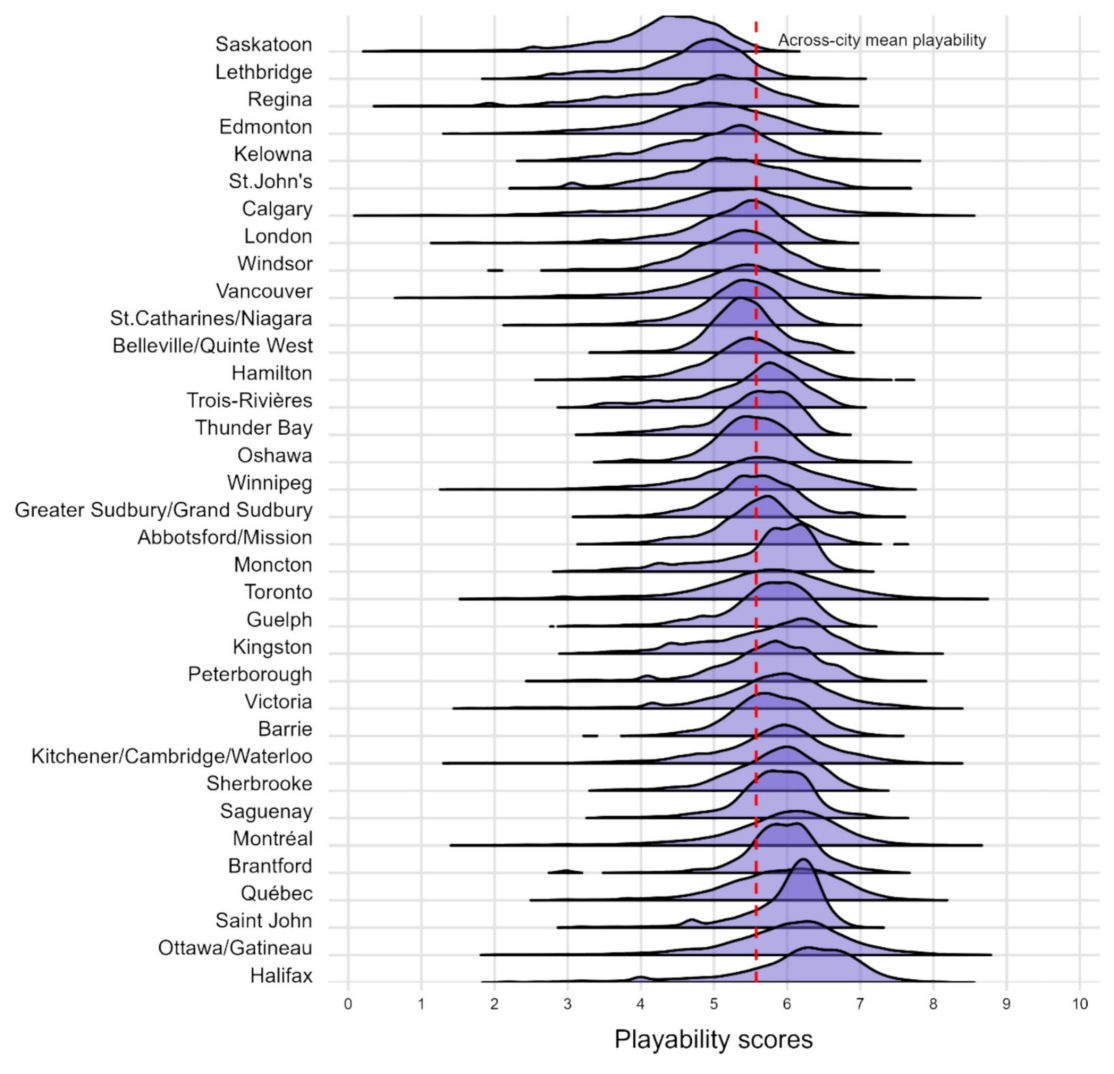
Density of postal code-level playability scores, by city.

Dramatic variation was seen in playability scores between neighbourhoods within the same city, with larger cities showing more variation in playability across neighbourhoods. In contrast, differences in playability averages between cities was relatively small, with a maximum difference of less than 20 % (1.7 points) (Fig. 4). Across study cities, an estimated 39.2 % (494,750) of all children under 5 and 37.4 % (1,548,880) of all children under 15 years lived in neighbourhoods (DAs) where the average for at least one playability domain was at or below the 10th percentile for all neighbourhoods.

### 2.3 Playability within study cities

Within-city composite playability scores (scores normalized and scaled for postal codes within each city) were positively correlated with material advantage in 20 of the 35 study cities, while nine cities showed negative, and six showed no significant correlations. In a majority of cities (30/35), materially advantaged neighbourhoods (DA-level) had higher average natural environment scores, while four showed negative and one showed no correlation (range: −0.07 to 0.52, *p* < 0.001). Traffic/pedestrian environment scores were also higher for materially advantaged neighbourhoods in 22/35 cities but were not correlated in seven, and negatively correlated with material advantage in six cities (range: −0.18 to 0.29, *p* < 0.05 to 0.001). However, social environment and child-relevant destination scores were more often negatively correlated with material advantage (19/35, range −0.33 to 0.32; and 24/35, range −0.40 to 0.32 cities, respectively, *p* < 0.05 to 0.001). The relationship between space for play domain scores and material advantage was variable across cities, with negative correlations in thirteen, positive correlations in sixteen and no relationship in five cities. (range − 0.40 to 0.32, *p* < 0.05 to 0.001). (Visualizations for relationships between playability score, domains and material advantage are available in Supplementary files, Sections C and D).

The relationship between within-city playability and population density (DA-level) was variable, with 21/35 cities showing negative, 11/35 showing positive and 2/35 showing no significant correlations. Child-relevant destinations and traffic/pedestrian environment domain score averages were higher in more dense areas for 34/35 cities (range: 0.14 to 0.74, *p* < 0.001), and 32/35 cities (range: − 0.18 to 0.51, p < 0.05 to <0.001), respectively. Conversely, natural and social environment score averages tended to be negatively associated with higher density in 32/35 and 29/34 cities, respectively (ranges: − 0.69 to 0.17, p < 0.001, and − 0.59 to 0.09, p < 0.05 to <0.001). Space for play correlations with density showed most variation across cities, with 12/35 showing positive, 14/35 negative and 9/35 no relationship with density (range − 0.22 to 0.27, p < 0.05 to <0.001). (Visualizations for relationships between playability score, domains and density are available in Supplementary files, Sections C and D).

**Fig. 5:**
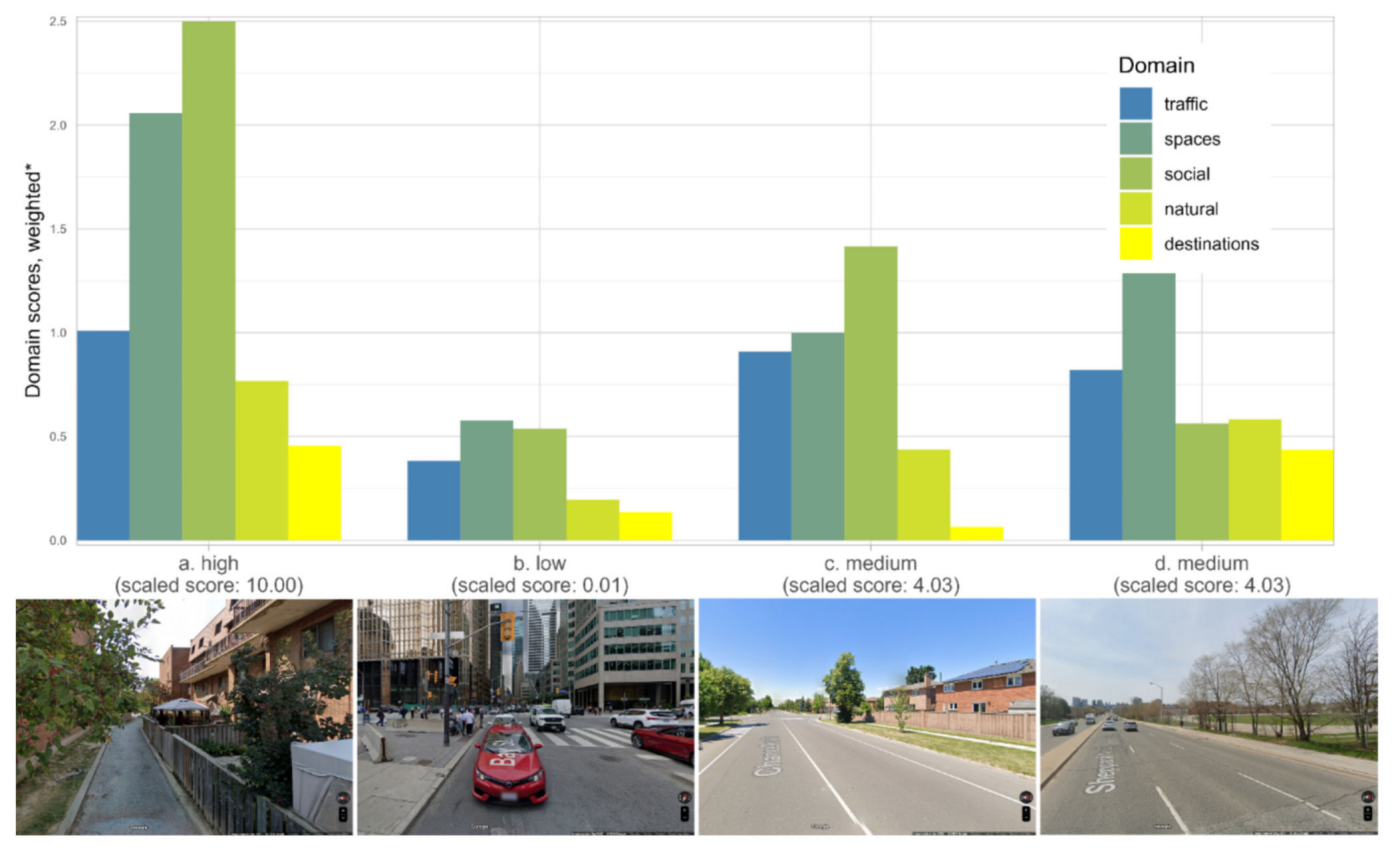
Weighted domain scores and street-view images for Toronto postal codes with high, low, and medium-range composite playability scores. *Domain scores are weighted as follows: traffic 0.17, spaces 0.26, social 0.25, natural 0.17, destinations 0.15. The sum of all weighted domain scores is then scaled to 0.0001–10.

The playability score combines domains that have differing spatial distributions within a city. Though neighbourhoods at the extremes of playability have relatively low or high scores across all domains, neighbourhoods with average playability show both strengths and weaknesses across domain scores (Fig. 5.). For instance, within Toronto, a postal code with a composite playability score of 10.0 has relatively high scores across traffic, space for play, social, natural and destination domains (Fig. 5.a) while another (composite score of 0.01) has low scores across domains (Fig. 5.b). However, two postal codes with nearly identical composite playability scores (4.03) have very different scores across domains (Fig. 5c. and 5d.). Decomposing the composite metric into separate domains allows identification of the drivers of low or high playability score in a particular location.

**Fig. 6:**
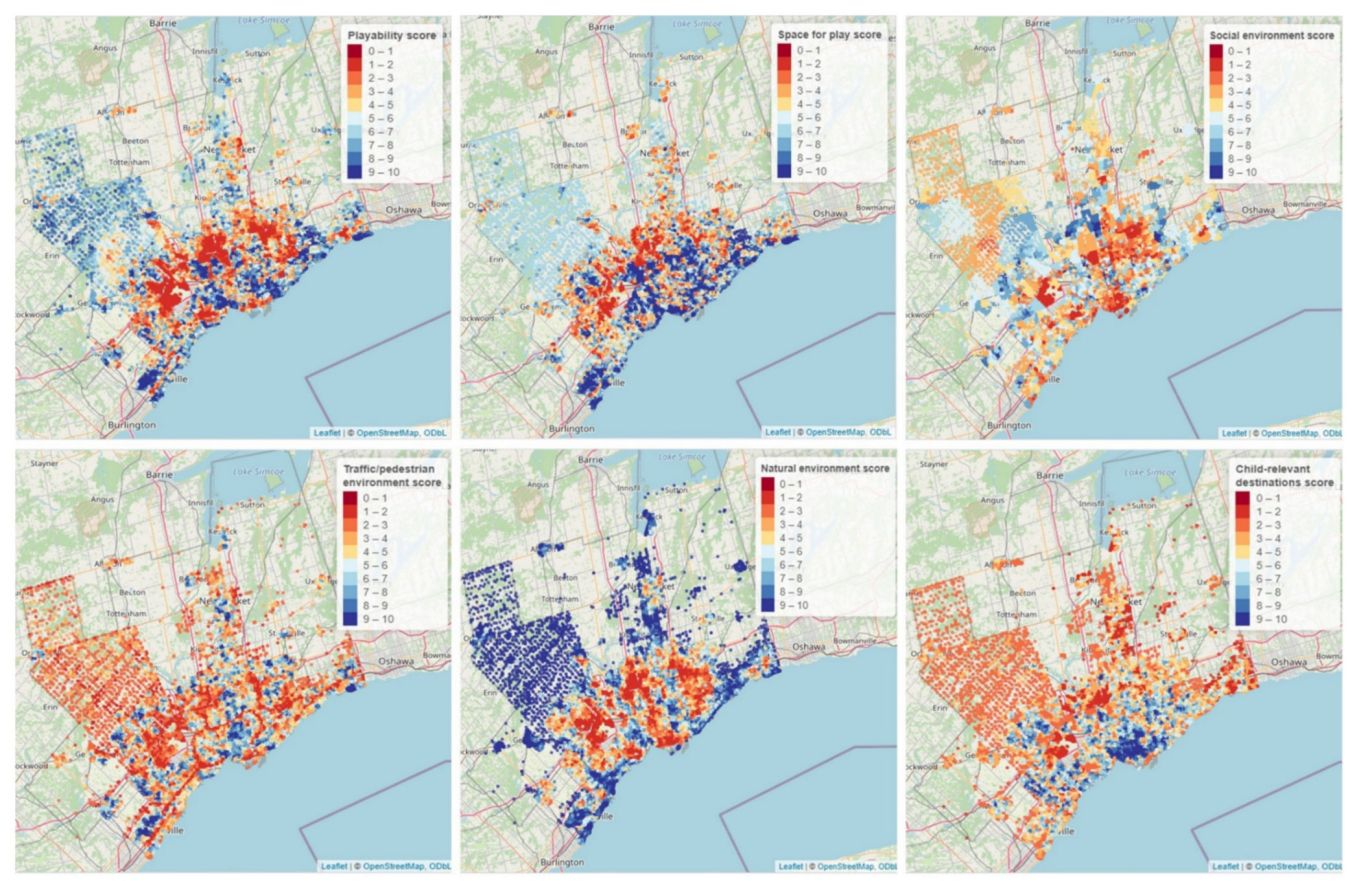
Distribution of playability and domain score deciles for postal codes within the Toronto metropolitan area. Playability score with alternate measure of social environment.

Mapping playability and domain scores at the postal code level across a city provides a high-level view of how characteristics of urban form that influence outdoor free play vary across neighbourhoods. Fig. 6. shows the distribution of playability and domain scores across Toronto Metropolitan Area. In general, city centers have better traffic and pedestrian environments, with slower speeds and better walking and cycling networks. However, natural environment scores tend to be higher further from the city center. An interactive web application, developed to disseminate playability data to local communities, policy makers, planners and the public allows exploration of playability scores and census tract-level characteristics for all study cities (https://playscore-ca-2-6.shinyapps.io/shiny/).

Social environment domain scores were correlated with higher material advantage when using the alternate SDI measure (ρ_s_ = 0.07, *p* < 0.001), (Supplementary files, Fig. C.14) in contrast to negative correlation with material advantage for the original social environment measure (ρ_s_ = −0.04, p < 0.001). The variable “proportion of children under 15” in the original social environment domain is a potential drivers of this difference, since families with young children may be less likely to live in the most affluent neighbourhoods.

## 3 Discussion

Using an evidence-based, theoretical framework for neighbourhood built and social environmental influences on young children’s outdoor free play, we developed a geospatial playability metric and mapped playability scores at the postal code level across the 35 largest cities in Canada. We found large within-city disparities in neighbourhood playability, indicating that children may experience vastly different environments for outdoor play within the same city. In contrast, across-city differences in playability scores were relatively small. Across-city playability scores were positively correlated with material advantage and negatively correlated with population density. Relationships between playability, material advantage and density within-cities were more variable; however, most cities showed a similar pattern of higher playability in more advantaged and less dense neighbourhoods. Individual playability domains revealed interesting and variable within-city relationships with material advantage and density. A majority of cities showed negative correlations between natural and social environment scores and density, and positive correlations between traffic/pedestrian environments, child-relevant destinations and density, while within-city relationships between spaces for play and density did not show a clear pattern across cities. Materially advantaged neighbourhoods had more favourable spaces for play, natural and traffic/pedestrian environments but lower social environment and child-relevant destination scores, in a majority of cities. These findings suggest trade-offs between playability indicators and domains between more and less affluent neighbourhoods and higher and lower density areas. In addition, they highlight the potential utility of a scalable, multi-dimensional index, which may be broken down to its constituent domains, to examine the drivers of playability at small spatial resolution. More than one third of all children in the study cities live in neighbourhoods at or below the 10th percentile for at least one playability domain, a finding that emphasizes the broad scope for improvement in the playability of neighbourhoods across Canadian cities.

This research makes theoretical, methodological and practical contributions to measurement of urban environments for children. The playability metric was based on a systematic review and synthesis of evidence from diverse urban contexts, which identified key barriers and facilitators to outdoor play across these settings. The theoretical framework developed from this review incorporated evidence from 17 countries with wide variation in geographic, cultural and social contexts, allowing generalization of the general framework to diverse urban settings. The subsequent methodological approach to indicator development, expert consultation, weighting and aggregation, may be applied in different contexts, with locally available data used to operationalize key themes. The major practical contribution of this work, an evidence-based, novel, urban playability metric for preschool to kindergarten age children (2 through 6 years), allows transparent assessment of neighbourhood supportiveness for young children’s out-door free play at a small spatial scale across 35 Canadian cities.

This research follows previous exemplary work on measurement of urban environments for children. Buck et al., adapted the well-known walkability measure (Frank et al., 2010) to incorporate child-relevant features such as public open spaces (e.g. green spaces, playgrounds) within a 1 km network distance of home address (Buck et al., 2011). Based on themes identified through a mixed-methods participatory study with 10–13 year-old children in Vancouver, Canada (Brussoni et al., 2020), Boyes developed a predictive model for unsupervised outdoor play (Boyes, 2023). The index included child and family-level characteristics, census tract-level demographics, speed limit, intersections, neighbourhood socio-economic deprivation, parks and other destinations, and visual features derived from street-view imagery (Boyes, 2023). Our work shares similar components with both indices, while making unique contributions to measurement of urban environments for children. A key difference was our theory-based approach to metric development. The theoretical framework was based on a comprehensive evidence synthesis that considered barriers and facilitators across diverse urban contexts, worldwide. Weighting of indicators was informed by an international group of experts. In contrast, data-driven models are often based on data from specific geographic and social contexts. Thus, the generalizability of concepts operationalized in the playability metric is supported by the underlying methodologic approach.

In development of indicators, we also considered evidence for the scale at which various indicators influence outdoor free play in young children. Rather than relying on administrative boundaries or standard buffer types and sizes, the playability metric operationalized each indicator at the most relevant scale possible, varying buffer sizes and types (e.g. network or Euclidean) and employing decay functions to most closely approximate exposures on the scale at which they are most influential to outdoor free play for young children. Accessibility to child-relevant destinations has often been measured without accounting for the impact of traffic exposure on children’s ability to reach these destinations. In a recent analysis, children’s access to urban destinations was estimated to be reduced by up to 75 % when traffic danger was considered (Tavakoli et al., 2024). The traffic/pedestrian domain of the playability metric captures traffic exposure in the immediate neighbourhood and along local walking/biking routes, providing additional context for the child-relevant destination measure. Finally, the playability metric developed here is the first urban measure focused specifically on outdoor free play in *early childhood*, at the very start of independent engagement with outdoor urban space. Much of the research on urban neighbourhood playability to date has understandably focused on older children (Brussoni et al., 2020), who generally have greater freedom to experience the neighbourhood. However, we chose to focus on neighbourhood playability for young children as the experiences and exposures of early developmental periods impact physical, social, emotional and cognitive health and wellbeing across the lifespan. In addition, young children may experience the most exclusion from local urban spaces due to the physical or social characteristics of these spaces, and parental perceptions of risk (Gemmell et al., 2022). This work contributes a quantitative measure that centers the interests and well-being of those who, arguably, have the least power and voice in the design and function of urban environments, despite the importance of these environments to their long-term health.

A major strength of this research was the application of quantitative and qualitative empirical evidence from diverse contexts, as well as expert opinion, to develop indicators of playability. We used open data sources that are widely available in other countries, whenever possible, to maximize the generalizability of our methods and share code for indicator development (QGIS Development Team, 2022; R Core Team, 2022). The major domains, relevant across widely diverse urban contexts, may be operationalized differently, depending on the setting, with local knowledge and data informing the development and weighting of individual indicators within each domain. For instance, though the domain “traffic and pedestrian environments” is critical to outdoor free play across urban contexts, cycling paths may not be relevant in all contexts. Adaptations of the index may use city-specific data that may provide improved measures for many indicators (e.g. sidewalk data, tree counts). Six of the 15 indicators utilized OpenStreetMap data, which is widely available across many countries, and others are available or may be derived from satellite imagery or street-view imagery for other contexts. (see Supplementary file A.8 for information on data sources and coverage). We also recognize some important limitations of the playability measure. The systematic review and evidence synthesis that informed development of the theoretical framework included a higher proportion of studies conducted in Westernized and higher-income countries. Other settings may have unique challenges and strengths: for instance, presence of hazardous waste and poor sanitation systems have been identified as barriers to children’s outdoor play in urban Ghana (Adjei-Boadi et al., 2022), while warmer weather, non-apartment housing types and supportive social norms enabled children’s outdoor free play in Somalia compared to the U.K (Allport et al., 2019). Thus, although the major domains are relevant across settings, further research is needed to identify unique barriers and facilitators for lower income and non-Westernized settings. Time and permission, in addition to space, are required for children to engage in outdoor play (Brussoni, 2020). Thus, family circumstances, values, cultural norms, time constraints and individual-level characteristics that also influence children’s engagement in outdoor play are not captured by this metric. The playability index is a relative, not absolute measure: playability and domain scores are determined by their comparative ranking among all postal code scores within or across study cities. Measuring the playability of urban neighbourhood environments presents challenges as determinants act and interact across multiple domains (e.g. social, natural, built environments) and at multiple scales (family, neighbourhood, city, regional, national). Data on neighbourhood characteristics is often inconsistently measured and available across cities (e.g. sidewalk networks), and to ensure data consistency across all study cities, we rely on proxies for some indicators of built environment features (e.g. road type as a proxy for traffic exposure and walking routes). In addition, the presence of some neighbourhood features does not necessarily indicate accessibility for outdoor free play (e.g. private vs. public greenspace). Future versions of the playability metric may be improved as better geospatial indicator measures become available. Parent et al. (2020) showed that outdoor play in young children was strongly associated with parental trust in neighbours, an individual-level, subjective assessment based on a persons’ characteristics, experiences and identity (Parent et al., 2020). An ideal measure of neighbourhood social environments for outdoor play would be a survey of residents to assess local social trust, however, such data are not available across study cities. We used small area (DA) level census variables as indirect means for estimating individual-level factors related to development of social trust (e. g. residential mobility, recent immigration). Acknowledging the challenges and complexity of characterizing social environments using existing data sources, we included both physical and social factors that influence opportunity for social connection across contexts. Future work to improve scalable measurement of social environments, through development of indices of social trust using census or other representative data sources, may provide an improved measure of neighbourhood social environments for outdoor play. The index provides a high-level measure of neighbourhood supportiveness of young children’s outdoor free play. However, it does not capture the nuanced, highly individual experiences, affordances and relationships within a neighbourhood so salient to the outdoor play of individual children. Future qualitative research with families and children within communities is needed to understand the complex, individual-level determinants of outdoor free play and to inform refinements to future versions of this high-level index.

Though many cities have developed plans that consider children’s inclusion and access to the city, scalable assessment of environmental supportiveness of children’s outdoor play are lacking. The playability index may enable cities to identify inequities, facilitators and barriers to everyday behaviours that promote child health and wellbeing. The playability of neighbourhoods around school and childcare centers may be examined, and facilities in less playable neighbourhoods prioritized for interventions that support children’s outdoor play (e.g. via implementation of Play Streets or School Streets programming) (Russell & Stenning, 2021). Planning for specific urban design interventions (e.g. traffic calming infrastructure, bike lanes, etc.) will require more detailed, city-specific information (e.g. traffic density data at specific intersections, street parking policies, etc.) than is provided by this measure. However, a high-level view of neighbourhood playability across the city provides a tool to amplify the unique perspectives and needs of urban children and prioritize areas for deeper engagement.. For instance, areas with low playability and a high proportion of children may be identified and participatory research conducted in the community to identify specific barriers and facilitators, and to guide local policy and action. More broadly, our finding of extremely wide variation in playability *within* cities, compared to relatively small differences *between* cities raises interesting questions about the scale at which child-friendly designations are most meaningful to children’s’ everyday experiences. Measurement of local neighbourhood supportiveness for young children's out-door play within cities may provide a practical and actionable measure of challenges and progress toward realization of children’s human rights “…in the small places, close to home…” (Roosevelt, 1999).

## 4 Conclusion

Play is young children’s primary mode of relating to their environments; their interactions with place demonstrate a deep desire to connect, understand and be in relationship with other species and humans (Ergler et al., 2021). This innate drive for connection may be supported or prevented by physical and social characteristics of urban neighbourhoods, acting through multiple pathways, including opportunity for outdoor free play, to influence life-long health and well-being. This research provides a novel, evidence-informed, high-level and scalable measure of neighbourhood playability for young children across major Canadian cities. We found wide within-city variation in neighbourhood playability, indicating that children within the same city experience disparities in quality of local environments for outdoor play. The playability metric may be used by cities to assess children’s equitable access to local opportunities for play and identify specific strengths or challenges within a neighbourhood. The theoretical framework and methodology used to develop this metric may be applied in diverse urban settings, to develop local measures of urban neighbourhood playability for young children.

## Supplementary Material

**Appendix A.Supplementary data**

Supplementary data to this article can be found online at https://doi.org/10.1016/j.cities.2025.106642.

Supplementary Files

## Figures and Tables

**Table 1:** Summary of playability metric components: domains, indicators, data sources, methods and weighting.

Domains (major themes)	Distinct Concepts (sub-themes)	+/–^a^	Data Sources^b^	Operationalization: description, scale of measurement	Indicator weight	Domain weight
**Space for play:**	Presence of nearby spaces designed for outdoor play	**+**	OpenStreetMap, CanMapContent Suite 2020 v.3 ParksSportsFieldRegion dataset	Number of parks or playgrounds within 500 m circular buffer of postal code centroid.	0.49	0.26
Neighbourhood space that is designed or may be adapted for play	Presence of open space in neighbourhood that may be adapted for play.	**+**	Canadian Urban Environmental Research Consortium	Percent of 250 m circular buffer around postal code centroid that is not part of building footprint (proxy for informal space for play)	0.51	
**Social environment:**	Knowledge of, connection to community places, people, institutions	**+**	Statistics Canada, 2016 Census	Reverse-coded proportion of 5-year residential mobility (DA-level^c^, a higher value indicates less residential mobility)	0.24	0.25
Parent and child sense of safety from social harm, opportunity for social connection with other children / community members, trusting and knowing neighbours		**+**	Statistics Canada, 2016 Census	Reverse-coded proportion of 5-year recent immigrants (DA-level^c^, a higher value indicates lower proportion of recent immigrants)	0.21	
	Presence of other children in the neighbourhood	**+**	Statistics Canada, 2016 Census	Proportion of children under 14 in neighbourhood (DA-level^c^)	0.55	
**Traffic environment**: Features of outdoor environment related to traffic that influence access to and experience of children’s outdoor play and independent mobility.	Exposure to vehicle traffic in the area around residence	–	Open Street Map road networks	Reverse-coded length and type of roads within 250 m circular buffer around postal code centroid, multiplied by coefficients representing relative traffic density and speed for each road type.^d^ (a higher value indicates less traffic).	0.33	0.17
	Exposure to road crossings en route to nearby destinations	–	OpenStreetMap road networks	Reverse-coded intersection density: number in 1000 m network buffer around postal code centroid. (a higher value indicates fewer intersections)	0.17	
	Availability of walking routes	**+**	OpenStreetMap road networks	Walking routes: total length of walking routes (pedestrian only streets, paths, local roads) over total length of roads in 1000 m network buffer	0.38	
	Availability of safe and comfortable cycling routes for children	**+**	Canadian Bikeway Comfort and Safety metric	Total length of cycling routes designated as medium or high comfort and safety by the CAN-BICS designation over total length of roads within 1000 m network buffer of postal code centroids.	0.12	
**Natural environment**:	Neighbourhood greenness	**+**	Canadian Urban Environmental Health Research Consortium	Normalized difference vegetation index (3 year average annual NDVI in 250 m circular buffer)	0.45	0.17
Setting that provides exposure to and opportunity to interact with nature	Presence of tree canopy	**+**	Canadian Urban Environmental Health Research Consortium and US Geological Survey	Tree canopy (percent coverage within 250 m circular buffer around postal code centroid)	0.35	
	Presence of blue space	**+**	CanMap Content Suite v.32020 Landcover data	Blue space (ocean, reservoir/pond/lake, watercourse/tidal river/side channel, canal, other waterbody. Percent bluespace within 1000 m circular buffer area around postal code centroid.	0.20	
**Child relevant destinations:** Services or destinations that are of interest to children and parents	Nearby destinations that may support active travel, incidental outdoor play, knowledge of local	**+**	OpenStreetMap, Statistics Canada’s Open Database of Educational Facilities (ODEF) and Open Database of Sports Facilities (ODSF)	Childcare, ECE centers, kindergarten, elementary schools (number within 1000 m network buffer around postal code centroid)	0.50	0.15
	areas and people	**+**		Library, community centre (number within 1000 m network buffer around postal code centroid)	0.25	
		**+**		Swimming pool, sports centre, recreation centre (number within 1000 m network buffer around postal code centroid)	0.25	
**Sensitivity analysis**: alternate measure of social environment	Family stress, social connection, family time and resources (reverse coded such that a higher score represents higher family social support connection, time and lower stress)	–	Social deprivation^e^ (Index is based on principle component analysis of census data and includes the following 2016 census variables and the dissemination area level: proportion who live alone proportion who have moved at least once in past 5 years proportion separated, widowed, divorced	Reverse-coded dissemination area-level Social Deprivation Index was reverse-coded (such that a lower score indicates more deprivation), linked to each postal code and rescaled to between 0.0001 and 10.	Individual SDI components were previously weighted and aggregated through PCA analysis (Pampelon, 2023).	0.25

a Direction of effect of the concept on outdoor free play.

b See Appendix Table B.8 for detailed information and licenses for data sources.

c Dissemination area, administrative small-area boundary with population between 400 and 700 persons.

d Coefficients were selected based on road classifications used in Canadian cities and are related to the relative traffic density and speed of each type of road. (see Appendix Table B.4).

e The SDI used separate PCA analyses conducted in four geographical zones: large CMAs, smaller CMAs, small towns, villages and rural areas. We used the data from the PCA for large CMAs.

## Data Availability

Data will be made available on request.
