# Supplementary material for: Development and application of a geospatial index of urban playability for young children
Source: Cities. Author manuscript; Available in PMC 2026 Mar 14. (PMC7618875; doi:10.1016/j.cities.2025.106642)
Supplement: Supplementary Files [file EMS212814-supplement-Supplementary_Files.pdf]

Supplementary Files
Section A.
Section A Tables provide a record of indicators considered, and the rationale for selection/non-selection for each major concept within a domain. References are included at the end of the document.

Table A.1. Spaces for play domain indicator selection

Concept	Potential Indicator	Data Source(s)	Availability	Indicator selection questions	Relevance/Impact	Simplicity	Modifiability	Decision	Main reason
Major themes within this domain that influence children's outdoor play in urban and suburban neighbour-hoods.	Potential measure for operation-alizing the concept.	Reference and link (if available) to indicator data source.	Is indicator data available at the relevant scale across all study CMA's?	1.How well does the potential indicator measure/ or act as a proxy for the concept.<br>2. How consistently does the potential indicator measure/partially measure concept over time, across diverse populations, settings, researchers.	How relevant/impactful is the potential indicator for the concept compared to other potential measures of the concept?	Is the indicator conceptually simple? Is it feasible to access/process the data with existing resources?	Is the indicator modifiable? Is the data consistently updated over time?		Primary reason for selecting or ruling this measure out as a playability indicator
			Yes	High/Medium	Medium	High	High	Yes	Reason
Formal spaces designated for outdoor play	Number of park (polygons) that overlap with a 250 meter circular buffer around postal code centroid.	OSM data, [1]<br>CanMap Content Suite 2020 v.3 [2]	A comparison of park polygons from CanMap vs. OSM shows fewer parks/playgrounds in CanMap compared to OSM and less detail in CanMap categories)	Strong qualitative evidence for importance of nearby parks[3,4] mixed quantitative evidence[5] with more urban areas showing more consistent and strong associations between parks and OFP. [3,4,6] In addition, social environment influences use of parks for OFP [7,8]<br>Parks important to OFP across contexts, with some exceptions Parks more important to OFP in denser areas because of less private outdoor space, home yards more important in less dense areas[3,4,6]	Likely to be highly relevant in urban settings, may be less relevant in sub-urban settings [3,9] Evidence for nearby parks as important to young children's outdoor play in both quantitative and qualitative literature . Evidence for the proximity, rather than size or amenities to be most important to young children's use of parks [10,11]	Conceptually simple (counts), though doesn't capture quality, size or amenities within parks.	May vary over the long term. Data updated at least yearly.		Strong evidence for importance of parks, especially in more dense urban areas. Some discrepancies seen between CanMap parks and OSM parks. We will combine both data sources for the "formal space for play" indicator.

Informal spaces that may be adapted for outdoor play	Percent of 250m buffer around postal code centroid that is not covered by building footprint. (proxy for informal space for play – may include streets, yards, vacant lots, temporary play spaces)		Yes	Medium/High	High	High	High	Yes	Reason
		Canadian Urban Environmental Health Research Consortium[12]		Strong conceptual link between open space in neighbourhood and OFP, supported by quantitative and qualitative evidence.[9,13–19]<br><br>This specific measure has been associated with time in outdoor play among 9-14 year olds.[20]<br><br>However, percent not covered by building footprint does not capture access to, suitability of space (e.g. may be private, unsafe road space, etc.)<br><br>Consistent qualitative evidence importance of nearby informal space for play and OFP, however, quantitative evidence for this particular measure is less abundant.	Likely highly relevant/impactful for OFP.<br><br>(Likely to also be correlated with density which may have an effect on OFP through social, traffic, destination pathways)<br><br>young children, in general, need less space for OFP[11] and need more supervision than do older children, so small nearby spaces may be more important than larger spaces farther away.	Conceptually simple.<br><br>However, percent not covered by building footprint around postal code does not indicate accessibility of space (e.g. may be private, unsafe road space, etc)	May vary over the long term.		Available across study cities. Data updated every few years. Conceptualizes potential (rather than actual) informal space for play in a neighbourhood.

Table A.2. Social environment domain indicator selection

Concept	Potential Indicator	Data Source(s)	Availability	Indicator selection questions	Relevance/Impact	Simplicity	Modifiability	Decision	Main reason
Major themes within this domain that influence children's outdoor play in urban and suburban neighbour-hoods.	Potential measure for operation-alizing the concept.	Reference and link (if available) to indicator data source.	Is indicator data available at the relevant scale across all study CMA's?	1. How well does the potential indicator measures/ or act as a proxy for the concept.<br><br>2. How consistently does the potential indicator measures/partially measures concept over time, across diverse populations, settings, researchers.	How relevant/impactful is the potential indicator for the concept compared to other potential measures of the concept?	Is the indicator conceptually simple? Is it feasible to access/process the data with existing resources?	Is the indicator modifiable? Is the data consistently updated over time?		Primary reason for selecting or ruling this measure out as a playability indicator
			Yes	Low	Medium	High	Medium	Yes	Reason

Opportunity for play with other children in neighbourhood	Proportion of children under 15 in neighbourhood (dissemination, small area level)	Statistics Canada 2016 Census microdata[21]	Available at the DA (or aggregated DA) level.	1.Strong conceptual link between availability of other children in the neighbourhood and OFP supported by qualitative evidence.[5] However, various measures of residential density of children in neighbourhood showed no link with OFP. [22–24]<br><br>This measure also does not account for the social context, which strongly influences whether play with other children is allowed/desired/supported. [25,26] The availability of other children to play with is necessary, but not sufficient for social play: neighbourhood physical/cultural/social contexts may largely determine if local children actually play together.[25–28]<br><br>2. Evidence for importance of opportunities for social play across contexts.[5]	Residential density of children may not be a good indicator of opportunity for social play. In the absence of a measure of neighbourhood social connection across study areas, it captures at least the potential for social play in local areas.	Conceptually simple, easy to measure.	Updated every 5 years		The physical and social context of a neighbourhood influence the degree to which local children play together. We included this variable due to the central importance of other children to play with (from qualitative findings) and the absence of other available measures of social connection across study CMAs.
			Yes	Medium/Medium	Medium		Medium	Yes	
Knowledge of, connection to local people, places, institutions	Proportion of residents who have immigrated in the past 5 years.	Statistics Canada 2016 Census microdata[21]		1.Experience of recent immigration (unfamiliarity with local context and places, social anxiety, lack of connection, parental stress, isolation) that intersected with features of physical environments to limit OFP.[26,29,30] However, these effects were reported at the family-level, rather than the neighbourhood level.<br><br>2. Evidence that recent immigration may be a barrier to OFP, as connections and trust require time to develop was seen across North American and European contexts.[26,29,30]	A potentially more robust measure of the concept could incorporate measures of social cohesion, collective efficacy, social capital and local organization infrastructure.[31]	Conceptually simple, easy to measure.	Updated every 5 years		Recent immigration may be linked to lower social and place connection. In the absence of measures of social cohesion, collective efficacy, social capital, organization infrastructure, across study cities, at a small spatial resolution, we use recent immigration as a proxy for lower social/place connection.
			Yes	Medium/Low	Medium	High	Medium	Yes	

Knowledge of, connection to local people, places, institutions	Proportion of residents who have moved within the past 5 years.	Statistics Canada 2016 Census microdata[21]	Yes	1.Knowing/trusting neighbours has been shown to influence children's OFP.[32] Because trusting relationships typically develop over time, high residential mobility may be a barrier to development of trust.[33,34]<br><br>2. Evidence across contexts that social connections, trust facilitate OFP.[32,35] However, limited evidence for specific measure (residential mobility in past 5 years) links to concept.<br><br>(We also considered residential mobility in the past year, however, this variable was not releasable due to privacy concerns related to small cell counts at the DA level)	A potentially more robust measure of the concept could incorporate measures of social cohesion, collective efficacy, social capital and local organization infrastructure.[31] Other potential proxies include measures of inequality (a barrier to social cohesion) and social exclusion.[34]	Conceptuall y simple, easy to measure.	Updated every 5 years		High residential mobility may be linked to lower social and place connection. In the absence of measures of social cohesion, collective efficacy, social capital, organization infrastructure, across study cities, at a small spatial resolution, percent of residents who have moved in the past 5 years will be used as a proxy for lower social/place connection.
			No	Low/Low	Low	High	High	No	Reason
Sense of social safety	Small area-level crime rates	Municipal datasets[36]	Crime rates may be available from municipalities,[36] however, they are usually not at a small-area level, inconsistencie s in reporting are likely and data may not be available for all municipalities in a metropolitan area.	Though conceptually plausible, crime rates have been inconsistently associated with parental perceptions of social safety related to children's outdoor activity.[37,38]	A potentially more robust measure of the concept could incorporate measures of social cohesion, collective efficacy, social capital and local organization infrastructure.[31]	Conceptuall y simple, difficult to measure.	May be updated continuously in some areas.		Not consistently linked to perceptions of social safety, data is inconsistently available across municipalities.
			Yes	Low/Low	Low/Low	High	Medium	No	

Neighbourhood deprivation	Material Deprivation Index[39]<br><br>(Index based on PCA analysis of census variables)	Available across all study cities at the DA level.[39]	Available across all study areas at the small-area level.	An Irish study reported lower participation in structured activity, but not in OFP in more deprived neighbourhoods.[40] Parental perception of neighbourhood as unsafe due to crime was higher in more deprived areas, however satisfaction with neighbourhood for raising children was also higher in these areas.[40] Physical disorder is generally associated with more OFP,[6,10,41] with some exceptions.[7,25]	Inconsistent evidence for neighbourhood deprivation as a barrier to OFP[6,40] Other characteristics of physical and social environments may mediate effect of neighbourhood deprivation on OFP.[5]	Conceptually simple, easy to measure	Updated every 5 years (Based on census data)		Inconsistently associated with children's OFP, though it may be linked to parental perception of social safety.[40]

Table A.3. Traffic/pedestrian domain indicator selection table

Concept	Potential Indicator	Data Source(s)	Availability	Validity/Reliability	Relevance / impact	Simplicity	Modifiability	Decision	Main reason
Major themes within the domain	Potential measure for operatio n-alizing the concept.	Reference and link (if available) to indicator data source.	Is indicator data available at the relevant scale across all study CMA's?	1. How well does the potential indicator measures/ or acts as a proxy the concept.<br><br>2. How well the potential indicator measures/partially measures concept consistently over time, across diverse populations, settings, researchers.	How relevant/impactful is the potential indicator for the concept compared to other potential measures of the concept?	Is the indicator conceptually simple? Is it feasible to access/process the data with existing resources?	Is the indicator modifiable? Is the data consistently updated over time?		Primary reason for selecting or ruling this measure out as a playability indicator
			No	Medium/Low	Medium	Medium	High		

Exposure to traffic in the area around residence	Traffic volume estimation model[42] (traffic flow dashboard data https://www150.statcan.gc.ca/n1/pub/71-607-x/71-607-x2022018-eng.htm)	Computer vision model for estimation of traffic volume using data from traffic cameras.[42] (experimental traffic flow dashboard data https://www150.statcan.gc.ca/n1/pub/71-607-x/71-607-x2022018-eng.htm)	Traffic cameras not available for all roads in study CMAs. Model applied to only a few sites.	1.Traffic volume influences safety, perceptions of safety for OFP.[3,7,10,13,19,22,29,35,40,43] but speed, vehicle type is also important. [14]<br><br>No evidence for association between this specific measure of traffic volume and OFP found.<br><br>Evidence for validity of measure in estimating traffic volumes compared to manual counts. [42]<br><br>2. Unclear. Initial evidence in Canada suggests a promising data source, but few testing sites. Needs replication.[42]<br><br>Consistent links between perceived traffic volume and OFP, perception of safety for OFP,[5,15] though no evidence found linking this particular measure of traffic volume with OFP.	Traffic volume is highly relevant to OFP.[3,7,10,13,19,22,29,35,40,43]<br><br>However, measure does not account for traffic vehicle type or speeds.	Conceptually simple (though doesn't capture some relevant characteristics of traffic)	Potential for real-time estimation of traffic volumes	No	Not yet available across all streets or CMAs, model tested in Canada does not assess vehicle type or speed. Low-volume, high speed roads likely to be a concern for OFP. Heavy vehicles result in more noise, air pollution than light vehicles - these factors may influence OFP.
			No	Medium/Low	Medium	Medium	Low		

Exposure to traffic in the area around residence	Annual Average Daily Traffic Volume (average 24hour traffic volume at a given location over a year)	BC data<br><br>https://prdoas6.pub-apps.th.gov.bc.ca/tsg/index.html#:~:text=AADT%20represents%20the%20average%20number,at%20various%20intersections%20around%20BC.<br><br>QC data<br>https://open.canada.ca/data/en/dataset/c77c495a-2a4c-447e-9184-25722289007f	Not available for all roads in study areas, AADT can be significantly different from one year to another, data is often outdated (>5 years) and often does not reflect current conditions.[44]	1.Traffic volume influences safety, perceptions of safety for OFP. [3,7,10,13,19,22,29,35,40,43] but speed, vehicle type is also important. [14] AADT commonly used in predictive models for collision along segments and intersections, simple counts can be used to estimate yearly average by annualization models. [45]<br><br>Strong conceptual link, but no evidence for association between this specific measure of traffic volume and OFP found. 2. Used widely in modeling vehicle traffic injury risk. AADT can be significantly different from one year to another, data is often outdated[44]	Traffic volume is highly relevant to OFP. Averaging over a year does not capture variability within smaller time frames (days, seasons). Measure does not account for traffic vehicle type or speeds. Evidence that heavy vehicles result in more noise, air pollution than light vehicles - these factors contribute to parental and child assessments of outdoor space for OFP.	Conceptually simple (though doesn't capture some relevant characteristics of traffic)	Data may not be available or up to date for all roads but major roads are assessed regularly.	No	Not available for all roads or CMAs, does not account for traffic vehicle type or speed. Low-volume, high speed roads are likely to be a concern for OFP. Evidence that heavy vehicles result in more noise, air pollution than light vehicles - these factors contribute to parental and child assessments of neighbourhood suitability for OFP.<br><br>Available from provinces and municipalities, frequency of measurement may vary across jurisdictions.
			No	Medium/Medium	Medium	Low	Low		
Exposure to traffic in the area around residence	Design hourly volume	Vancouver data<br><br>https://vancouver.ca/streets-transportation/traffic-count-data.aspx	Not available for all roads and in all CMAs.	Traffic volume influences safety, perceptions of safety for OFP. [3,7,10,13,19,22,29,35,40,43] but speed, vehicle type is also important. [14]<br><br>Commonly used in road design decision-making.[44] Conceptual link but no direct evidence for association between measure and OFP found.	Focused on intersections	May be complicated to incorporate variation related to time of day into metric	Although it provides real-time estimation, hourly estimates may be too fine for our purposes.	No	Not available for all roads or CMAs, does not account for traffic vehicle type or speed. Different municipalities may use various methods for short-term count data.
			Low	Low/Low	Low	Low	Medium		

Exposure to traffic in the area around residence	Collisions per million entering vehicles (MEV)	National Data available at CMA level – (no geocoding) https://wwwapps2.tc.gc.ca/Saf-Sec-Sur/7/NCDB-BNDC/p.aspx?l=en	Not available for all roads, information may not be up to date.	Though parental fear of traffic injury is a strong barrier to OFP, assessment of risk not based on or consistently correlated with data.[37]<br><br>CMEV is a reactive measure used to prioritize road safety infrastructure.[44] Does not take into account proactive parental action (not allowing OFP or active travel.[46] Parental perceptions of traffic safety (which may influence OFP) has not been consistently linked to actual traffic danger – and may be moderated by parental and neighbourhood social characteristics.[37]	Perception of traffic safety (proactive injury prevention) is more relevant parent/child decision making about OP/active mobility[46] Though important information, does not reflect most of the day-to-day considerations that influence OP: comfort, noise, perceptions, traffic volumes, speed.	Conceptually simple	Able to reflect trends or pre-post changes in specific areas.	No	No evidence that measure correlates with parent/child perceptions of traffic safety for OFP.<br><br>Does not capture non-collision traffic impacts (perceptions, air pollution, noise, comfort) Low-use intersections may have high rate with few collisions.
			Yes	Medium/High	Medium	High	Low	No	
Exposure to traffic in the area around residence	Road Type	CanMap Content Suite 2020 v3, [2] https://abacus.library.ubc.ca/dataset.xhtml?persistentId=hdl:11272.1/AB2/FHWOBD	Available for all roads across all CMAs.	Road type classifications are used to define purpose and use of roads (volume, speed, vehicle type). Road type also linked to environmental noise,[47] air pollution,[48] factors that may influence OFP.[5] Road type classifications may vary across jurisdictions.<br><br>Reasonably consistent road type definitions and corresponding traffic volumes, speed across Canadian jurisdictions[49–52]	Though updated infrequently, road type generally captures traffic speed, volume and primary use purposes.[51] Approximating traffic density and traffic type, characteristics that influence comfort, noise, perceptions of safety for OFP.<br><br>Generally (though imperfectly) reflects traffic densities and speeds.	Conceptually simple. Road type captures many outdoor-play relevant characteristics of traffic (e.g. speed, volume, vehicle types).	Does not reflect daily, weekly or seasonal temporal trends. Re-designation of road type is likely to be a slow process (e.g. in response to traffic increases over time)	No	Though this could be a reasonable measure, but some evidence that OpenStreetMap is a more complete and accurate data source for road networks and types in Canada.[53]
			Yes	Medium/High	Medium	High	Low	Yes	
Exposure to traffic in the area around residence	Road Type	Open Street Map road networks<br><br>[54]	Open Source. Available across all CMAs.	Road type classifications are used to define purpose and use of roads (volume, speed, vehicle type).[51]<br><br>Traffic volume, type and speed are linked to parental perceptions of the safety and quality	Though updated infrequently, road type generally captures traffic speed, volume and primary use purposes.[51]	Conceptually simple. Road type captures many outdoor-play	Does not reflect daily, weekly or seasonal temporal trends. Re-designation of		Evidence that OSM road networks are more up to date than other road networks available across Canada [53]

				of neighbourhood routes to and spaces for OFP.<br><br>Additionally, they are linked to environmental noise,[47] air pollution,[48] that may influence OFP.[5]<br><br>Strong conceptual link, some measure-specific evidence (e.g. pedestrian-only, residential streets)[55]<br><br>2. OSM road types are consistent and well-defined across study areas.[54]<br><br>Consistent support for conceptual link between road type (as proxy for traffic speed volume, type).[5,15,56]	Approximating traffic density and traffic type, characteristics that influence comfort, noise, perceptions of safety for OFP. Evidence that OSM road networks more up to date than other road networks available across Canada [53]	relevant characteristics of traffic (e.g. speed, volume, vehicle types).	road type is likely to be a slow process (e.g. in response to traffic increases over time)		
			No	High/High	High	High	Medium	No	
Exposure to traffic in the area around residence	Road Width	Could be calculated using a machine learning model and satellite imagery.<br><br>(Municipal sources may have right-of-way data, but this does not necessarily measure curb-to-curb widths https://opendata.vancouver.ca/explore/dataset/right-of-way-widths/information/)	Not available. Calculation possible but time/effort intensive.	1.May be a good approximation of the expected traffic volume and speed of a road regardless of road designation.<br><br>Evidence for link between road width, traffic volume and speed for some road types.[57,58] Small negative correlation (-0.17) (not significant) found between road width and average outdoor play among 9-14 year-olds in Dhaka city (unadjusted model only).<br><br>2.Consistent evidence for link between road width, traffic volume and speed for some road types.[57,58]	Good proxy for traffic density, type and speed as more lanes/wider roads are built to accommodate higher traffic volumes/ heavier vehicle types. In addition, narrower roads often slow traffic regardless of posted speeds.	Conceptually simple. May capture many relevant characteristics of traffic (speed, volume, vehicle types)	Potential for measurement based on municipal records or using machine learning model and satellite imagery to be updated frequently.	No	Potential as a good proxy for traffic exposure influencing OFP. Calculation possible but time/effort intensive.

			Yes	High	High	High	Medium	Yes	
Exposure to traffic en route to local destination [11,59]	Intersections	Can calculate intersections from OSM road networks[54]		1.Evidence that intersections are a barrier to children's access to play spaces. [11] More intersections associated with less time in OP.[22]<br><br>Greater street connectivity may be linked to higher perceptions of traffic danger [60,61]<br><br>Intersections negatively correlated with avg outdoor play time, (unadjusted, ages 9-14)[20] and negatively associated with PA in 4 year old boys. [62]<br><br>2. Evidence consistent for negative impact of intersections on OFP and/or independent mobility across studies, settings for younger age groups,[11,20,22,60,62] but intersections are positively correlated with active travel in older adolescents.[62]<br><br>(However, even in younger age groups, more intersections may also be associated with more nearby child-relevant destinations)	Evidence for high relevance of intersection density to traffic exposure along routes.<br><br>Potential for high impact of intersections (as proxy for traffic exposure en route to nearby destinations).	Conceptually simple. Measure is consistent across diverse contexts.	May be updated with same frequency as road network data (OSM data – continuously updated esp. in urban areas.	Yes	Overall, intersections are negatively linked to young children's OFP. This indicator is easy to calculate and update.<br><br>(However, more connectivity may also be linked to better access to child-relevant destinations within walking distance, so CRD indicator and intersection indicator may partially cancel one another.)
			No	High	High	High	Low	No	Reason
Exposure to traffic en route to local destination [11,59]	Sidewalk networks	Municipal open data (e.g. https://geoweb.dnv.org/data/, https://vancouver.ca/files/cov/sidewalk-priorities-map-2023-2027.pdf)	Open data not available across all municipalities in all CMAs (e.g. no open sidewalk data for St. John's)	1.Evidence for importance of sidewalk networks to OFP in urban areas. [19,22,63,64]<br><br>2. Overall, evidence consistent for positive association between sidewalks and OFP across urban contexts.[19,22,63,64]	High relevance, potential impact of sidewalks on active mobility, OFP in local areas.	Conceptually simple.	Unclear how frequently, consistently data is updated across municipalities.		Not all study CMAs have sidewalk network open data, different data and data portals for municipalities within CMAs. Unclear how up to date sidewalk data is across study municipalities/CMAs. Time and labor intensive to collect, consolidate data.

			No	High	High	High	Low	No	Reason
Exposure to traffic en route to local destination [11,59]	Sidewalk networks,<br><br>Walking-only streets, paths, trails	OpenStreetMap sidewalk networks	OSM has incomplete sidewalk networks for many study cities	1. Evidence for importance of sidewalk networks, paths to OFP in urban areas. [19,22,63,64]<br><br>2. Overall, evidence consistent for positive association between sidewalks and OFP across urban contexts.[19,22,63,64]	High relevance, potential impact of sidewalks on active mobility, OFP in local areas.	Conceptually simple.	Existing OSM data is not accurate in some cities, unclear how often data is updated in cities with accurate data.	No	OSM sidewalk data is incomplete in many study cities.<br><br>(We randomly sampled 10 locations along road network without sidewalks in OSM, then verified using GSV satellite imagery. In the 15 CMAs sampled, all had at least 10% (1/10) missing sidewalks and 10 CMAs had 30% (3/10) or more sidewalks missing for the locations checked.)
			No	High/High	High	High	High	No	Reason
Exposure to traffic en route to local destination [11,59]	Sidewalk Networks	Potential to calculate sidewalk networks using a computer vision model and satellite/street-view imagery[65]	Not available. Calculation possible but time/effort intensive.	1.Evidence for importance of sidewalk networks to OFP in urban areas. [19,22,63,64]<br><br>2. Overall, evidence consistent for positive association between sidewalks and OFP across urban contexts.[19,22,63,64]	High relevance, potential impact of sidewalks on active mobility, OFP in local areas.	Conceptually simple.	Would be relatively simple to update at the rate sidewalk network changes.	No	Calculation possible but time/effort intensive.<br><br>(Plan for PlayScore 2.0!)
			Yes	High/High	High	High	High	Yes	Reason

Exposure to traffic en route to local destination [11,59]	Paths, Pedestrian-only streets, roads classified as "local" roads. (proxy for sidewalk networks)	OpenStreet Map	Yes	Evidence for importance of sidewalk networks to OFP in urban areas. [19,22,63,64] Additionally, pedestrian-only, quiet,[19] low speed, low traffic volume streets facilitate OFP.[14] In Canadian urban areas, "local road" is the lowest speed, lowest traffic-volume designation next to pedestrian-only street.[54] Local roads in Canadian urban areas often have sidewalks (even when missing in OSM data). 2. Overall, evidence consistent for positive association between sidewalks and OFP across urban contexts. [19,22,63,64] (Though not all studies showed association [41,64] Evidence that low vehicle traffic streets and pedestrian-only streets are supportive of OFP and children's active travel.	High relevance, potential impact of "walking routes" to active mobility, OFP in local areas.	Not as conceptually simple as sidewalk network, but includes important additional information on potential walking routes (e.g. pedestrian-only streets)	Pedestrian-only streets and local roads are accurate in OSM data [53] however, accuracy of path network in OSM has not been evaluated.	Yes	Because of the high missingness of sidewalk data in OSM, we will use a proxy measure based on OSM local roads (lowest traffic speed, volume of all vehicle road classifications), merged with OSM paths and pedestrian-only streets.
			No	Medium/Medium	Medium	Medium	Low	No	Reason
Exposure to traffic en route to local destination [11,59]	Pedestrian crossings	Municipal data?	Municipal data not available across all municipalities, study CMAs.	1.Evidence for importance of pedestrian road crossings to OFP. [11,19,22] 2. May be more important where traffic speeds, volume, road widths are higher (number of crossings, type and characteristics of roads, traffic volume, speed)	Relevant to active mobility and OFP. Depending on neighbourhood and traffic context, may have more or less influence on OFP.	Conceptually simple	Accuracy and up-to-date status of pedestrian infrastructure data is unclear across municipalities	No	Availability of pedestrian crossings likely influences children's access to OFP (through parental/child perceptions of safety). Data on road crossings is not available across all study CMAs, unclear how updated existing datasets are.
			Medium	Medium/Medium	Medium	High	Medium	No	Reason
Exposure to traffic en route to local destination [11,59]	Cycling networks	OSM data	Unclear how complete OSM cycling data is, OSM does not include measure of quality for cycling paths.	1.Evidence for importance of child-appropriate cycling networks to children's OFP, active mobility OSM cycling networks are geared toward adult mobility, may not be safe or appropriate for child active travel, especially for young ages. 2.Consistent evidence for importance of cycling networks perceived as safe by parents and children.[13,19,40,63] (though specific cycling infrastructure may be less important on very low traffic roads)	Relevant to children's active mobility, OFP. In general, safe walking routes are likely to be more critical than cycling routes in supporting OFP for young children.	Conceptually simple	Accuracy and up-to-date status of cycling network data is unclear.		OSM data do not classify cycling network comfort or safety – important factors in making them accessible to children (especially young children).

				High/High	Medium/Low			Yes	Reason
Exposure to traffic en route to local destination [11,59]	Cycling network s	Canadian Bike Network and Safety[66,67]	Unclear how complete OSM cycling data is across jurisdiction, however, applying the Can-BICs classification to existing OSM cycling networks provides a measure of quality/appro priateness for young children's use.	Evidence for importance of child-appropriate cycling networks to children's OFP, active mobility[13,19,40,63] (though specific cycling infrastructure may be less important on very low traffic roads) Strong conceptual link between safe/comfortable cycling networks and OFP though no specific evidence for Can-BICs – OFP association.<br><br>OSM cycling networks are geared toward adult mobility, many not safe or appropriate for child active travel, especially for young ages. Can-BICS classification system, applied to OSM cycling networks can indicate quality/appropriateness for young children's use.[66,68]	Relevant to children's active mobility, OFP.<br><br>In general, walking routes are likely to be more important than cycling routes in supporting OFP for young children.	Conceptuall y simple	Accuracy and up-to-date status of OSM cycling network data is unclear.	Yes	Cycling network comfort and safety are important factors in making them accessible to children (especially young children). OSM data is available across all CMAs, though unclear how complete and updated.

Table A.4 Road type classifications and coefficients

Road Type Variable*	Open Street Map	Ottawa Road Classification Summary [69]	B.C. Highway Functional Classification [70]	City of Toronto Road Classification System [49]	Primary Function	Speed km/hr	Traffic Volume vehicles/day**	Co-efficient to characterize relative traffic volume and speeds
**Local_Road**	residential service	Local Road	Local Road	Local Road	access to property, generally no bus routes	<40	< 2,500	2
**Major_Road**	secondary, secondary_link unclassified tertiary tertiary_link	Collector Roads<br><br>Major Collector	Minor Road	Collector Road<br><br>Minor Arterial	support traffic movement, parking and access to residences possible	40 –60	2,500 – 8,000 (collector)<br><br>8,000 – 20,000 (minor arterial)	4
**Highway**	trunk trunk_link primary	Arterial Roads	Secondary highways Major Roads	Major Arterial	primarily support traffic movement	50-60	>20,000	6
**Motorway**	motorway motorway_link	City Freeway	Primary highway	Expressway	prioritize traffic movement and speed, no property access, pedestrians/ cyclist prohibited	80-100	>40,000	8

* Road type classifications used in playability metric variable "Road Type" to indicate relative traffic volume and speeds, and corresponding classifications in other Canadian systems.
** Speed and traffic volumes for road types varied slightly across cities, City of Toronto values are presented here.

Table A.5 Natural environment domain indicator selection

Concept	Potential Indicator	Data Source(s)	Availability	Indicator selection questions	Relative relevance and impact	Simplicity	Modifiability	Decision	Main reason
Major themes within the domain	Potential measure for operation-alizing the concept.	Reference and link (if available) to indicator data source.	Is indicator data available at the relevant scale across all study CMA's?	1. How well does the potential indicator measures/ or acts as a proxy for the concept?<br>2. How well does the potential indicator measures/partially measures concept consistently over time, across diverse populations, settings, researchers?	How relevant/impactful is the potential indicator for the concept compared to other potential measures of the concept?	Is the indicator conceptually simple? Is it feasible to access/process the data with existing resources?	Is the indicator modifiable? Is the data consistently updated over time?		Primary reason for selecting or ruling this measure out as a playability indicator
			No	High/High	Medium	Medium	High	No	
Presence of trees in neighbourhood	Street tree count, extracted from google street view images	[71]	Yes (potentially).	Strong qualitative evidence for trees being valued by children and parents as supporting OFP. [16,17,27,72] Adult perception of playability was associated with tree count using street-view imagery.[38]	Only captures trees seen from the street.	Conceptually simple, feasible.	Could potentially extract trees from street view imagery at regular intervals.		Possible, but only captures trees seen from the street.
			Yes	High/High		High	Medium	Yes	
Presence of trees in neighbourhood	Tree canopy	[12]	Yes (Canue repository is missing data from St. John's but possible to calculate this from USGS satellite data)	Strong qualitative evidence for trees being valued by children and parents as supporting OFP across contexts.[16,17,27,72]	Captures vegetation over 2m in height across study areas. May be a more complete measure than one based on street trees.	Conceptually simple. Data available for almost all study areas from Canue. (able to calculate from satellite data)	Satellite data is updated every 10 years		Captures vegetation above 2m in height across study areas.
				High/High	High	High	High	Yes	
Neighbourhood greenness	Normalized difference	[12]	Yes. (CANUE repository contains datasets for	1. Quantitative evidence for neighbourhood greenness supporting OFP.[72,73] Strong qualitative evidence	Widely used measure of neighbourhood greenness, has been associated with	Conceptually simple. Data available across study cities.	Can be derived from satellite data every year.		

	vegetation index.		postal code level measures of NDVI for multiple years)	for benefits of greenspace to young children's OFP.[16,29,74,75]<br><br>2. Evidence for NDVI links to OFP and physical activity is fairly (but not completely[76]) consistent across urban contexts.	behavioural and health outcomes for children.[72,77]				
				Medium/Medium	Medium		High	Yes	Reason
Blue Space	Waterbodies (rivers, canals, lakes, ocean)	CanMap Content Suite 2020v.3<br><br>Landcover	Yes.	1.Qualitative results highlight children's strong affinity for water and blue spaces.[17,78,79] However, blue spaces may also pose risks to young children and heighten parental perception of danger.<br><br>2.Though water is likely to be attractive to children across diverse settings, mention of these features generally occurred in studies conducted in coastal areas or areas with rivers, etc.[17,79]	Small-scale blue spaces may be more important to everyday OFP for young children (e.g. ditches, water features, puddles). The Landcover dataset captures larger water features that may also support OFP, but likely require closer caregiver supervision, potentially limiting access.	Conceptually simple. Data available across study cities.	Updated every year.		Despite limitations (may not be relevant in all contexts), will include blue spaces as highly valued by children for OFP. A better measure may include small-scale blue spaces (ditches, etc) that are likely to be more widely available across contexts. However, currently such data is not available.

Table A.6. Child-relevant destinations domain indicator selection

Concept	Potential Indicator	Data Source(s)	Availability	Indicator selection questions	Relevance / impact	Simplicity	Modifiability	Decision	Main reason
Major themes within the domain	Potential measure for operation-alizing the concept.	Reference and link (if available) to indicator data source.	Is indicator data available at the relevant scale across all study CMA's?	1. How well the potential indicator measures/ or acts as a proxy the concept.<br>2. How well the potential indicator measures/partially measures concept consistently over time, across diverse populations, settings, researchers.	How relevant/impactful is the potential indicator for the concept compared to other potential measures of the concept?	Is the indicator conceptually simple? Is it feasible to access/process the data with existing resources?	Is the indicator modifiable? Is the data consistently updated over time?		Primary reason for selecting or ruling this measure out as a playability indicator
Nearby destinations that may support active travel, incidental outdoor play, knowledge of local areas and people [74,80,81]				High/High	High	Medium	Low/High	Yes	
	Schools, childcare centers, ECE centers	OpenStreet Map, Statistics Canada's Open database of educational facilities	Yes. However, the data is likely to be incomplete for childcare centers. Comparison with Vancouver childcare center registries showed many childcare centers missing from both data sources as available sources include only public childcare facilities.	Strong evidence for proximal, everyday destinations supporting OFP.[5]	Highly relevant. About half of all young children attend childcare, nearly all kindergarten age children in Canada attend school.	Conceptually simple.	Number of schools/child care centers changes slowly.		Nearby schools, ECE centers may be some of the most important destinations for supporting OFP, as they are accessed regularly and promote interaction with other local children.
			Yes	Medium/Medium	Medium	High	Low/High	Yes	

	Library, community center	OpenStreet Map, Statistics Canada's Open database of educational facilities		Evidence indicates that proximal public spaces, institutions support development of social cohesion and connections.[82,83] Conceptual link for neighbourhood resources, destinations supporting OFP.[3,17,78]		Conceptually simple	Number, locations of libraries and community centers change slowly, influenced by local population density and resources.<br>.		Strong conceptual evidence for neighbourhood institutions and destinations supporting incidental OFP and local social connections.
			Yes	Medium/Medium	Medium	High	Low/High	Yes	
	Swimming pool, sports centre, recreation centre	OpenStreet Map, Statistics Canada's Open Database of Sports Facilities		Evidence indicates that proximal public spaces, institutions support development of social cohesion and connections, active mobility. Evidence for local recreation centres supporting OFP.[19,40,84]		Conceptually simple	Number, locations of sports centres, rec centres change slowly, influenced by local population density and resources.<br>OSM data updated continuously, ODEF updated every year.		Strong conceptual link

Table A.7. Operationalization of Selected Indicators

Indicator	Data Source	Details
Road type	OpenStreetMap road network[54]	OpenStreetMap road network data (OSM key = "highway") [1] was downloaded using the osmdata package in R [85].<br>Variables were recoded as follows: motorway, motorway_link recoded as "Motorway"; trunk, trunk_link, primary, primary_link recoded as "Highway"; secondary, secondary_link recoded as "Major_Road"; unclassified, tertiary, tertiary_link, residential, road, service recoded as "Local_Road"; living_street, pedestrian recoded as "Walking_Street"; footway, path recoded as "Path".<br>Road length for each road type within a 250m circular buffer of postal code centroid was calculated.<br>Road lengths were multiplied by coefficients representing the relative traffic volume and speed for each road type (see Appendix B.4)<br>Totals for all road types were then summed to estimate exposure to vehicle traffic within a 250 m circular buffer of postal code centroids.
Intersections	OpenStreetMap road network data [1]	We derived intersection point data for intersections of all local roads, major roads, highways and motorways using QGIS 3.24.2.<br>We then calculated the number of intersections within a 1000m network buffer of postal code centroids using the r5r package in R, [86,87]<br>We reverse-coded the intersection indicator so that a higher value represents a lower number of intersections.
Walking networks	OpenStreetMap road network data [1]	Walking environments for children were represented by summing the total length of OpenStreetMap variables (feature key = "highway"): "unclassified", "tertiary", "tertiary_link", "residential", "road", "service" (recoded as "Local_Road"); "living_street", "pedestrian" (recoded as "Walking_Street"); and "path" (recoded as "Path") within a 1000m network buffer of postal code centroids, using the r5r package. Before summing, walking route types were weighted based on their estimated relative protection from traffic: Local Road x 2, Path x 4 and Walking Streets x 6. To allow for comparisons of walking environments between high and lower density areas, we then divided the total walking route sum by the total road lengths (Local Road, Major Road, Highway, Motorway).
Cycling networks	Canadian Bikeway Comfort and Safety Classification System (Can-BICS) dataset [88,89]	Cycling route data was obtained from the Canadian Bikeway Comfort and Safety Classification System (Can-BICS) dataset [88,89] We calculated the total length of cycling routes classified as high or medium comfort and safety within a 1000m network buffer of each postal code centroid using the r5r package in R [90]. High and medium comfort and safety designations were weighted by multiplying the total length of each type by coefficients 6 and 4, respectively. We summed the cycling route lengths and

		divided by the total road lengths within 1000m buffer distance (Local, Major Roads, Highway, Motorway lengths)
Formal Spaces for Play	CanMap Content Suite[2]	We recoded the variables PARK/SPORTS FIELD, RECREATION, DAY USE, BOTANICAL GARDEN, NATURAL ENVIRONMENT, PICNIC SITE, NATURE RESERVE, RECREATION AREA, PROVINCIAL PARK, NATURAL AREA and PARK RESERVE from the CanMapContent Suite 2020 v.3 ParksSportsFieldRegion dataset as “park”[2].<br><br>We downloaded and recoded OpenStreetMap variables (feature key = “leisure”) “common”, “park”, “dog_park” and “playground” as “park”. We combined both datasets, unioned overlapping polygons, and summed the number of unique polygons within a 500m circular buffer of each postal code centroid using the sf package [91].
Informal Spaces for Play	CANUE[12]	The percent area within 250m circular of buffers of postal code centroids that is covered by building footprints was obtained from the Canadian Urban Environmental Research Consortium database [92]. Building footprints used by CANUE to derive this measure were calculated using high resolution satellite data and machine learning methods described by Setton et al.[92] We reverse-coded this value to represent the percent area *not* covered by building footprint as a proxy for neighbourhood open space that may be adapted for play.
Percent of population 0-14 years	2016 Statistics Canada Census of Population Microdata[93]	Data from the 2016 Statistics Canada Census was used to represent features of the social environment that influence outdoor free play. This data is available at the dissemination area (DA) level. Percent of population under age 15, percent of residents who moved from outside the census sub-division (CSD) within the last 5 years and percent of residents who immigrated within the last 5 years were calculated for each DA and linked to postal codes within those DAs. We aggregated data for DAs with very small populations until total and residual counts for each variable met Statistics Canada requirements for release.[21]
Percent residents who moved from outside the census sub-division (CSD)* within the last 5 years.	2016 Statistics Canada Census of Population Microdata	
Percent residents who immigrated	2016 Statistics Canada Census of Population Microdata	

within the last 5 years		
NDVI	CANUE[12]	Normalized Difference Vegetation Index (NDVI). Annual average NDVI within 250m circular buffer around each postal code was obtained from the Canadian Urban Environmental Research Consortium database, indexed to DMTI Spatial postal codes. NDVI was derived by CANUE from Top of Atmosphere (TOA) reflectance data in bands from cloud free annual composites of USGS Landsat 5 and Landsat 8 satellite images.[94–99]
Tree canopy		Tree canopy (area of vegetation of woody plants with height above 5m) within 250m circular buffer around each postal code centroid was obtained from the postal code-linked Tree Canopy dataset available on the Canadian Urban Environmental Research Consortium database for 34 of the 35 study areas. These estimates were derived from the Global Forest Cover Change (GFCC) Surface reflectance product, based on high-resolution Landsat 5 Thematic Mapper (TM) and Landsat 7 Enhanced Thematic Mapper Plus (ETM) images. [100] For St. John's census metropolitan area, no tree canopy data was available from the GFCC for the years represented in CANUE tree canopy datasets. To obtain estimates for St. John's we downloaded GFCC tree canopy data for 2005 from USGS Earth Data, and calculated the percent tree canopy coverage within 250m of each postal code location. [101,102]
Blue space		Percent area covered by blue space within a 1000m circular buffer of postal code centroids was calculated from the CanMap Content Suite 2020 v.3 LandCoverRegion dataset.[2] We subset the landcover data to that classified as "WATERBODY", including waterways, streams, rivers, lakes and oceans, and calculated the percent covered by any waterbody within 1000m buffer of postal codes using the sf package. [91]
Child-relevant destinations (schools, community centers/libraries, sports facilities)		We downloaded school, community and recreational facility locations from OpenStreetMap [1], Statistics Canada's Open Database of Educational Facilities (ODEF) [103] and Open Database of Sports Facilities ODSF) [104], removed duplicates and calculated number of each type within a 1000m network buffer distance from postal code centroid using OSM road networks and the r5r package [90]. The score for each child-relevant destination was set to decline (via and exponential decay function) with distance from postal code, with a value of 1 at the postal code declining to 0.5 at 1000m.
Social Deprivation Index		This measure represents small-area (DA) level concentration of family characteristics that may influence children's outdoor play through social support, family time, stress and resources. The index is derived from Statistics Canada 2016 Census data and includes the following variables: proportion of residents who live

(alternative measure of social environment)		alone, proportion who have moved, and proportion separated, widowed or divorced. The dissemination area level SDI score was scaled to 0.001 -10 and reverse-coded (such that a lower score indicates more deprivation) and assigned to the postal codes within each dissemination area.[105]

* Census subdivision (CSD) is the general term for municipalities or areas treated as municipal equivalents for statistical purposes.[106]

Table A.8. Indicator data licenses

Indicator	Data source(s)	Reference(s)	Link to data terms of use, license	Data source coverage*
Road type	OpenStreetMap	1	www.opendatacommons.org/licenses/odbl	Nearly globally
Intersections	OpenStreetMap	1	www.opendatacommons.org/licenses/odbl	Nearly globally
Walking routes	OpenStreetMap	1	www.opendatacommons.org/licenses/odbl	Nearly globally
Cycling Routes	Canadian Bikeway Comfort and Safety Classification System	66	https://opendatacommons.org/licenses/odbl	Canada
Formal space for play	OpenStreetMap<br><br>DMTI Spatial CanMap Content Suite 2020 v.3	1,2	www.opendatacommons.org/licenses/odbl<br><br>https://abacus.library.ubc.ca/dataset.xhtml?persistentId=hdl:11272.1/AB2/FHWOBD	Nearly globally<br><br>Canada
Informal space for play	Canadian Urban Environmental Research Consortium	92	Canadian Building Footprint data is licensed under the open data commons license https://opendatacommons.org/licenses/odbl/	Canada

			CANUE data use governed by the Data Sharing and Use Agreement https://www.canuedata.ca/metadata.php	
Percent DA population under 15 years	Statistics Canada, Census of Population, 2016	21	Totals for the three variables were calculated at the dissemination area level. DA-level data was then aggregated as needed to meet Statistics Canada privacy standards, with DAs with very low number of residents aggregated with adjacent DAs. Data from Statistics Canada Census 2016 was vetted to meet privacy requirements and released for use in the approved research project.	Canada
Percent DA residents who have moved within past 5 years				Canada
Percent DA residents who have immigrated within past 5 years				Canada
NDVI	Canadian Urban Environmental Research Consortium	94–97,99	CANUE data use governed by the Data Sharing and Use Agreement https://www.canuedata.ca/metadata.php	Canada (NDVI is widely available across countries from other data sources or may be derived from satellite imagery)
Tree canopy	Canadian Urban Environmental Research Consortium	100	Tree canopy data available through USGS Earth Data. Terms of use at https://lpdaac.usgs.gov/data/data-citation-and-policies/	Canada<br><br>(tree canopy estimates for other countries may be available from other sources, or may be derived using satellite imagery)

			CANUE data use governed by the Data Sharing and Use Agreement https://www.canuedata.ca/metadata.php	
Blue space	CanMap Content Suite 2020 v.3	2	https://abacus.library.ubc.ca/dataset.xhtml?persistentId=hdl:11272.1/AB2/FHWOBD<br>See "Terms"	Canada
Child relevant destinations - schools	Open Database of Educational Facilities<br><br>Open Database of Sports Facilities<br><br>OpenStreetMap	1,103,104	https://open.canada.ca/en/open-government-licence-canada	Canada
Child relevant destinations – recreational facilities			https://open.canada.ca/en/open-government-licence-canada	Canada
Child relevant destinations – community facilities			https://open.canada.ca/en/open-government-licence-canada	Canada<br><br>(OSM is available nearly globally)
Alternate measure of neighbourhood social environment (for sensitivity analysis)	Material and Social Deprivation Index<br><br>(only the Social Deprivation Index component used in sensitivity analysis)	105	Publicly available metric based on Statistics Canada Census 2016 data.	Canada

*Data quality and completeness may vary across contexts

Section B. Expert Survey

Expert survey recruitment

Potential experts were identified as authors of peer-reviewed publications on topics relevant to children's OFP in urban areas, and conducted their professional work in 10 different countries (Canada, Germany, USA, Australia, Ghana, Ireland, UK, New Zealand, Norway, Bangladesh). Participants were emailed an invitation to participate, along with a link to the online survey and consent form.

Table B.1. Expert Survey

**PlayScore: developing a measure of neighborhood playability for young children**<br>**Expert survey**	
**Instructions**	
Thank you for sharing your knowledge, experience and expertise on neighborhood environments and children's outdoor play!<br><br>In this survey you will be asked to series of questions about neighborhood characteristics that may influence children's outdoor play in urban and suburban contexts. These playability domains and indicators have been selected based on literature review and available data.<br><br>You will be asked to assign points to indicate your view of the relative importance of the dimensions and potential indicators for two age groups: children 0 - 6 years, and children 7-12 years.*<br><br>You will also have an opportunity to provide additional comments at the end of the survey.	
**Demographics**	
Which of the following best describes you? (select all that apply)	
Academic researcher	
Urban planner	
Educator	
Child rights advocate	
Health professional	
Other (please explain)	
In what location(s) do you conduct your professional work or research? (select all that apply)	

Asia	
Africa	
Australia	
Europe	
North America	
South America	
**Domains**	
Below are 5 broad domains that may influence children's outdoor play in urban and suburban settings. Based on your knowledge and expertise, and considering urban and suburban contexts, allocate points to each category to reflect your assessment of its relative importance to young children's (0-6 years) outdoor play. (points must total 100).	
Availability of outdoor space for play (formal or informal)	
Traffic environments	
Social environments	
Natural environments	
Child relevant destinations	
Total	
Considering the same 5 dimensions, allocate points to each category to reflect your assessment of its relative importance to 7-12 year old children's outdoor play. (points must total 100).	
Availability of outdoor space for play (formal or informal)	
Traffic environments	
Social environments	
Natural environments	
Child relevant destinations	
Total	
**Indicators**	
**Availability of outdoor space for play**	
In your view what is the relative importance of these potential indicators for the dimension “availability of outdoor space for play” to 0-6 year old children's outdoor play? (points should total 100)	

Percent of neighborhood space not part of building footprint (proxy for yard or informal space)	
Number of parks and or playgrounds in the neighborhood	
Total	
In your view what is the relative importance of these potential indicators for the dimension “availability of outdoor space for play” to 7-12 year-old children's outdoor play? (points should total 100)	
Percent of neighbourhood space not part of building footprint (proxy for yard or informal space)	
Number of parks and or playgrounds in neighbourhood	
Total	
**Natural environments**	
In your view, what is the relative importance of these potential indicators for the domain “natural environments” to 0-6 year old children's outdoor play? (points should total 100)	
Greenness of neighborhood (Normalized Difference Vegetation Index)	
Bodies of water in neighbourhood (river, lake, stream, canal, ocean shore)	
Tree canopy (percent area covered)	
Total	
In your view what is the relative importance of these potential indicators for the domain “natural environments” to 7-12 year old children's outdoor play? (points should total 100)	
Greenness of neighbourhood (Normalized Difference Vegetation Index)	
Bodies of water in neighbourhood (river, lake, stream, canal, ocean shore)	
Tree canopy (percent area covered)	
Total	
**Traffic environments**	

In your view, what is the relative importance of these potential indicators for the domain “traffic environments” to 0-6 year old children's outdoor play? (points should total 100)	
Road type (highway, major road, residential, pedestrian only, etc.)	
Sidewalks, paths, trails	
Cycling paths	
Number of intersections in neighbourhood	
Total	
In your view, what is the relative importance of these potential indicators for the domain “traffic environments” to 7-12 year old children's outdoor play? (points should total 100)	
Road type (highway major road, residential, pedestrian only, etc.)	
Sidewalks, paths, trails c	
Cycling paths	
Number of intersections in neighbourhood	
Total	
**Child-relevant destinations**	
In your view, what is the relative importance of these potential indicators for the domain “child-relevant destinations” to 0-6 year old children's outdoor play? (points should total 100)	
Child care center, kindergarten, primary school,	
Library, community center	
Sports facility, swimming pool	
Total	
In your view, what is the relative importance of these potential indicators for the domain “child-relevant destinations” to 0-6 year old children's outdoor play? (points should total 100)	
Child care center, kindergarten, primary school,	
Library, community center	
Sports facility, swimming pool	

Total	
**Social environments**	
In your view, what is the relative importance of these potential indicators for the domain "social environments" to 0-6 year old children's outdoor play? (points should total 100)	
Residential mobility (proportion of residents who have moved within the past five years)	
Recent immigration (proportion of residents who have immigrated in the past five years)	
Proportion of neighbourhood residents 0-14 years	
Total	
In your view, what is the relative importance of these potential indicators for the domain "social environments" to 7-12 year old children's outdoor play? (points should total 100)	
Residential mobility (percent in neighborhood who have moved within the past five years)	
Recent immigration (percent in neighborhood who have immigrated in the past five years)	
Proportion of neighbourhood residents 0-14 years	
Total	
**Additional comments:**	
Please add any comments related to your responses above.	
Are there any additional domains or indicators that you feel should be considered?*	

* The second age group, 7-12 years, was included to compare expert opinion on the importance of neighbourhood features to OFP for younger vs. older children.

****** We considered this feedback in final indicator selection and selection of alternate social environment measure.

Expert survey participant characteristics

Of 26 invited experts, 20 participated in the online survey (77% response rate). Of these, 14 (60%) conducted their professional work or research in North America, 3 (15%) in Australia, 4 (17%) in Europe, 1 (5%) in Asia, and 1 (5%) gave no location. Participants self-identified as academics, researchers, child rights advocates, urban planners, architects, educators, consultants, recreation or health professionals.

Table B.2. Expert-assigned mean domain weights for early and middle childhood.

Domain	Early childhood (0-6 years) (mean weight, sd)	Middle childhood (7-12 years) (mean weight, sd)	Difference in median[a] p
Spaces for play	26 (11.58)	21 (7.45)	0.11
Traffic/pedestrian environments	17 (9.34)	22 (9.12)	0.02*
Social environments	25** (12.35)	25 (8.10)	0.96
Natural environments	17 (6.74)	15 (4.72)	0.12
Child-relevant destinations	15 (10.05)	17 (8.10)	0.29

*Wilcoxon signed rank test for paired samples.
** Averages for both spaces for play and social environments were 25.6. We rounded social environments down (25) and spaces for play up (26) to achieve a total of 100 for all domains, rounded to whole numbers. We made this rounding decision as the median value for social environments was more than 8 points below that of spaces for play.

Table B.3. Differences in expert-assigned indicator weights for early and middle childhood.

Domain	Indicator	Early childhood (0-6 years) (mean weight, sd)	Middle childhood (7-12 years) mean weight (sd)	Difference in means[a] p
Spaces for play	Formal spaces	49 (18.89)	56 (13.98)	0.06
	Informal spaces	51 (18.89)	44 (13.98)	0.06
Traffic/ pedestrian environments	Road type	33 (15.10)	33 (14.36)	0.89
	Intersections	17 (10.82)	16 (8.56)	0.38
	**Walking routes**	**38 (9.66)**	**30 (8.35)**	**0.003****
	**Cycling routes**	**12 (9.38)**	**21 (8.37)**	**0.002****
Social environments	Residential mobility	24 (11.46)	24 (12.45)	0.67
	Recent immigration	21 (11.22)	19 (10.87)	0.17
	Children 0-14 years	55 (18.99)	57 (18.44)	0.39

Natural environments	Greenspace (NDVI[b])	45 (12.25)	43 (10.55)	0.53
	**Blue space**	**20 (10.50)**	**27 (9.47)**	**0.02***
	**Tree canopy**	**35 (8.26)**	**30 (9.80)**	**0.02***
Child-relevant destinations	**Schools, childcare centers**	**50 (13.28)**	44 (12.76)	**0.02***
	Community centers, libraries	25 (6.17)	23 (8.32)	0.43
	**Recreation centers**	**25 (9.39)**	**33 (8.18)**	**0.0006*****

[a]p value for Wilcoxon signed rank test for non-normal distributions of differences for paired samples, $p < 0.05$ = *; $p < 0.01$, = **; $p < 0.001$ = ***

Table B.4. Qualitative responses from expert survey*

**Participant**	**Participant feedback**	**Changes, comments in response to feedback**	
Spaces for play			
R1	the importance of proximal public green space is more important for those without private outdoor space, something that may not necessarily be captured in your survey	Yes, this aligns with the expert survey quantitative results that assigned nearly equal weighting to parks and informal space for play. In areas with higher density, number and proximity of parks is generally higher across the study cities, the reverse is true for lower density areas, where parks are less frequent, but private yards and other informal spaces are usually more common. Equal weighting means that one type of space is not automatically prioritized over the other.<br>(We acknowledge that we are constrained by available data across the study cities, which does not provide information on accessibility of space, boundaries of private spaces, etc. Future versions of the index may be able to obtain information on lot sizes to better estimate private space boundaries.)	
R4	What is the difference between a playspace and natural space?	A play space may also be a natural space (e.g. a park with many natural elements), but not every play space is a natural space and vice versa. We consciously chose to measure both play spaces and natural spaces, though there is some overlap, as they represent distinct dimensions, based on our systematic evidence review.	

R8	I think proximity is important as well as the kind of neighbourhood-wide features you explore.	This feedback prompted us to consider the relevant buffer size for each indicator as well as to consider step-wise or exponential decay functions to increase/decrease the effect of the indicator based on the distance from the postal code centroid.	
Social Environment			
R2	The social domain is very difficult to get at and I suspect will be largely unsuccessful at predicting outdoor play with these kind of data. A tricky task!	Our goal is not necessarily to predict outdoor play (as we are unable to measure many important individual and family-level determinants that influence behaviour), but to characterize neighbourhood environments that are most supportive of play, informed by an evidence-based theoretical framework. We do acknowledge that we are limited by the available data and future versions of the index may benefit from more precise measures of neighbourhood social environments.	
R3	Surprised that income is not considered.	We considered income as a potential indicator of neighbourhood social environments for outdoor play, however, evidence for the influence of neighbourhood income on children's physical activity and outdoor free play is inconsistent.[6,107,108] On one hand, parent perception of social disorder may be more common in lower income neighbourhoods and pose a barrier to children's outdoor play,[25,26,109] on the other, lower income neighbourhoods may have more children, or less advantaged children may participate in fewer formal programs and thus engage in more outdoor play.[40,110] Low household income may influence outdoor free play in young children due to parental time and resource constraints. Conversely, high parental education (education is often used as a proxy for household income) has been linked to less outdoor play in several studies. We chose not to include income in the index, due to this inconsistency in the literature.	
R3	Unsure where the questions about immigration come from.	Our rationale for selection of the indicator "proportion who have immigrated in the past 5 years" as a proxy for connection to local people, places and institutions, is based on evidence	

		from North American and European contexts,[26,29,30] and evidence that increased familiarity and incidental local social connectedness tends to increase with increasing time in a neighbourhood.[111] However, we acknowledge that this is an imperfect proxy that does not capture the full range of experiences or community settings.	
R5	…tolerance of children outdoors. For the latter, there are some areas in Australia e.g. see https://www.lookupstrata.com.au/children-noise-complaints-in-apartments/ for apartment blocks, but similar issues arise for adjacent parks etc. It is definitely a divisive issue.	Yes, this may be a significant barrier in Canadian settings.[112] Unfortunately, we lack data on toleration of children outdoors across the study cities. Future surveys and qualitative work is needed to explore the extent of the influence of local social pressures, bylaws and norms on children's access to outdoor spaces.	
R4	…social environments are not defined in the beginning and you create social environments and social gathering places in physical spaces e.g. playgrounds, nature spaces, too.	In response to this comment, we have tried to emphasize that the *social environment domain* seeks to capture "connection to local places, people and institutions, sense of safety from social harm and presence of other children" We agree that there is overlap between indicators of the social environment, and indicators of physical environments (e.g. natural spaces, play spaces, traffic environments) that may promote or inhibit social interaction.	
R6	Social environment could include more indicators eg, children's family information such as the number of family members, family income and so on) and the proportion of older adults living in the neighbourhood (intergenerational play), in case, data is available.	Individual and family-level variables are not available across the study cities, due to privacy protections. We considered including the proportion of older adults in the neighbourhood, however, we did not identify this as a clear determinant of children's outdoor play in our review of the literature.	
R7	I think parents working hours should be considered. Questions like what is their commute time, does their language and/or cultural of origin factor into community connections, how much control do the parents have with their work schedules are some factors that I have noticed affect a child's access to formal and informal outdoor play in an urban environment. Thank you for asking.	These are very important considerations which could be explored in a qualitative study. As a sensitivity analysis, we used the Social Deprivation Index (which includes proportion of residents who live alone, proportion who have moved, and proportion separated, widowed or divorced) to capture neighbourhood-level data on family structure and support that could influence parental time and resources.	

R8	For most of the dimensions, I thought there were other factors that might have featured on your list (eg for social, something about adult attitudes).	Yes. Although data is currently not available across the study cities, future surveys or qualitative work could explore these important determinants and additional nuanced information not captured in the index.	
R10	For social environments, I would recommend adding items about perceived social norms and social cohesion. I would rate them as more important than the percentage of immigrants and the percentage of recent movers.	Agree. The ideal measure of social environments would be a survey of residents. Future work may use survey data from specific communities to better characterize their local social environments.	
	**Traffic Environment**		
R3	The traffic component should include traffic volume.	We considered various measures of traffic volume. However, data on objectively measured traffic volume was not available across study cities. The selected measure of traffic exposure is derived from road classifications, which are based on the road's intended use and consider type of vehicle, speed and volume. Future work may incorporate estimates of actual use if these data become available.	
R4	…you also ask the same a few times in the same question(e.g. traffic and roading and then you can also tick cycle paths etc.)	In a Canadian context, cycling paths may be part of the roadway (e.g. painted bike lanes) or separated in various ways.	
R9	Traffic calming/speed (tied with perceptions of traffic/safety)	Speed is currently incorporated into the traffic exposure measure. Traffic calming may be available at the city level in various forms, however, no consistent data is available across study cities, to our knowledge. Future versions may incorporate more detailed data on traffic and pedestrian environments.	
	**Other comments**		
R3	Hi, a couple comments: The five basic domains are initially very broad, I'm not sure the first question is all that pertinent.		
R3	Generally, this survey is very vague, but that may have been the intent! Thanks and best of luck :)		

R4	Something to consider more broadly for your survey: you have a very North American, European focus in your questions e.g. someone who works in the Pacific or Pacific Rim countries cannot tick any boxes; you also ask the same a few times in the same question(e.g. traffic and roading and then you can also tick cycle paths etc.);	A majority of available evidence on urban built environments and outdoor play has been carried out in North American / European contexts. Future work in other contexts is needed.	
R5	…potential indicators are: weather…	Yes, weather may be a significant barrier/facilitator. For this index we focus on measuring modifiable features of urban neighbourhoods rather than including all factors that may predict outdoor play.	
R8	…many of the answers are highly context- and culture-specific - I found myself thinking I'd answer very differently if I were thinking about the Netherlands compared to Canada.	This is a great point, highlighting that the development and weighting of indicators to capture the broad playability domains should consider local context. We have added this point to our discussion of the generalizability of the theoretical framework to support development of playability indices in diverse settings.	
R11	Perhaps cultural background and how this can affect children's permissions to go out to play. And a follow on from this the role that gender plays in children being allowed to go out into a neighbourhood for outdoor play	Agree that these are important determinants of children's outdoor play. The goal of the index is to characterize the playability of neighbourhood environments rather than to predict outdoor play – however, built environments may interact with cultural and parenting norms to influence outdoor play. This comment emphasized the importance of considering cultural context in the operationalization of indicators based on the broad theoretical framework.	

*Expert responses to "Please add any comments related to your responses above. Are there any additional domains or indicators that you feel should be considered?

Section C.

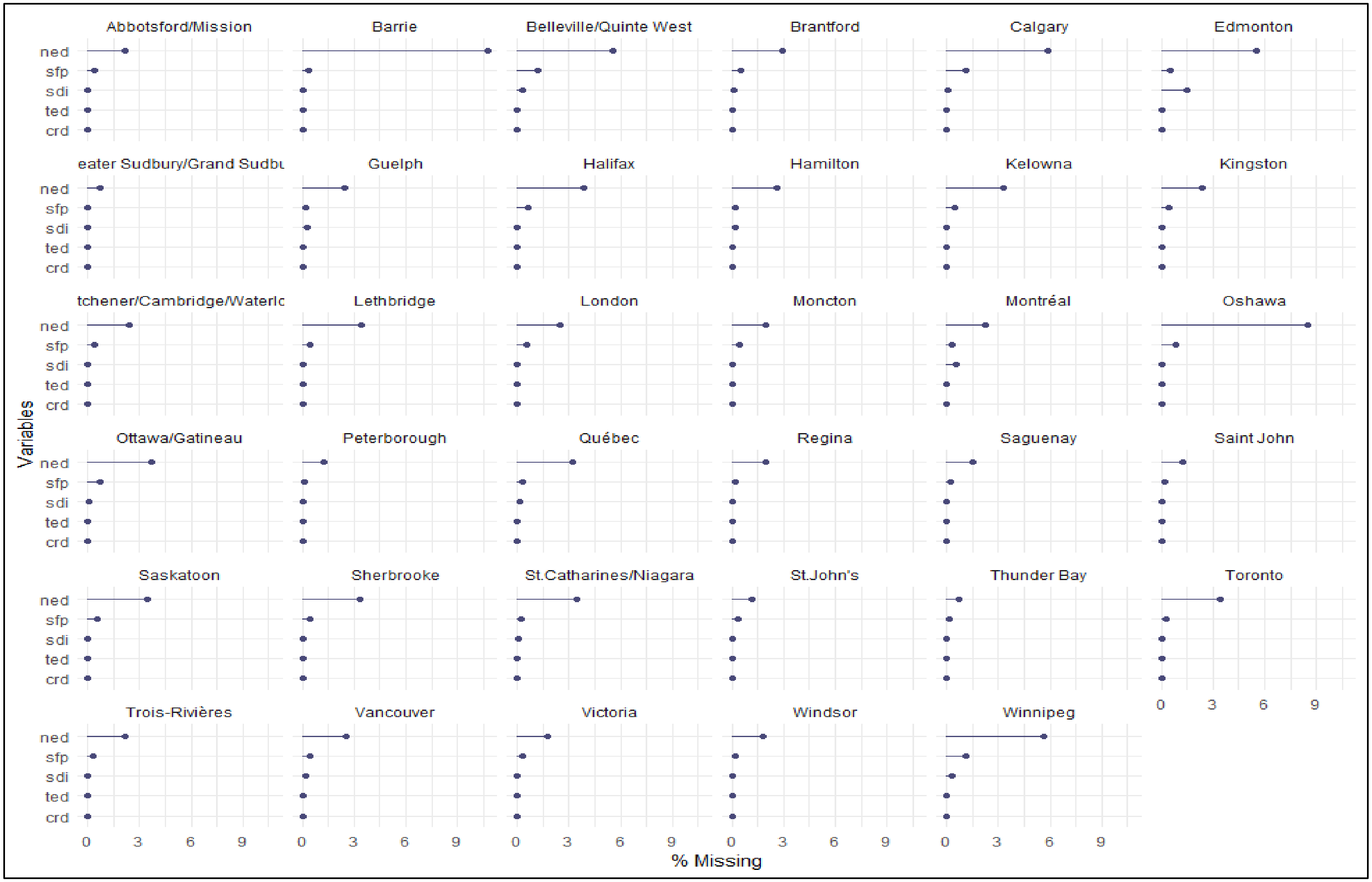

*ned : natural environments, sfp: space for play, sdi: social environment, ted: traffic environment, crd: child-relevant destinations

Figure C.1. Percent of postal codes missing data for playability domains within all study cities.

Table C.1. Characteristics of study cities

Census Metropolitan Area	Population	Land Area (km^2)	Percent population under 5	Percent population under 15	Proportion low income after tax**
Toronto	6202225	5902.75	4.67	15.56	10.2
Montreal	4291732	4670.10	5.10	16.82	12.0
Vancouver	2642825	2878.93	4.34	14.08	11.1
Ottawa - Gatineau	1488307	8046.99	4.98	16.89	8.8
Calgary	1481806	5098.68	5.71	18.50	8.6
Edmonton	1418118	9416.19	5.90	18.60	8.8
Quebec	839311	3499.46	4.85	15.92	8.9
Winnipeg	834678	5285.46	5.36	16.91	12.2
Hamilton	785184	1373.15	4.97	16.02	9.4
Kitchener/Cambridge/Waterloo	575847	1092.33	5.34	17.12	9.3
London	543551	2661.48	4.97	16.51	12.4
Halifax	465703	7276.22	4.52	14.89	12.0
St. Catharines - Niagara	433604	1397.09	4.51	14.48	11
Windsor	422630	1803.17	4.82	16.28	11.3
Oshawa	415311	903.25	5.66	18.50	7.3
Victoria	397237	695.29	3.81	12.71	8.9
Saskatoon	317480	5864.48	6.08	19.10	11.0
Regina	249217	4323.66	5.97	18.99	10.2
Sherbrooke	227398	1458.10	4.73	15.84	13.6
Kelowna	222162	2902.45	4.04	13.78	9.5
Barrie	212856	897.26	5.09	17.01	7.7
St. John's	212579	931.56	4.30	14.85	11.3
Abbotsford - Mission	195726	606.72	5.33	17.66	8.3
Kingston	172546	1919.17	4.42	14.38	10.1
Greater / Grand Sudbury	170605	4187.40	4.54	15.44	9.6
Guelph	165588	595.08	5.04	16.27	7.7
Saguenay	161567	3133.53	4.69	15.20	11.4
Trois-Rivieres	161489	1038.64	4.46	14.56	14.3
Moncton	157717	2562.47	4.45	15.52	12.5
Brantford	144162	1074.00	5.27	17.12	9.4
Saint John	130613	3505.66	4.49	15.47	13.2

Census Metropolitan Area	Population	Land Area (km^2)	Percent population under 5	Percent population under 15	Proportion low income after tax**
Peterborough	128624	1508.44	4.44	14.72	10.8
Lethbridge	123847	2958.96	6.02	19.01	9.6
Thunder Bay	123258	2550.79	4.50	14.33	11.0
Belleville - Quinte West	111184	1337.50	4.78	15.53	10.5

*based on 2021 census [113]

**calculated according to the low-income thresholds depending on household size (e.g. after tax income of $26,503 CDN for one person, $45,904 CDN for three persons in 2021) [114]

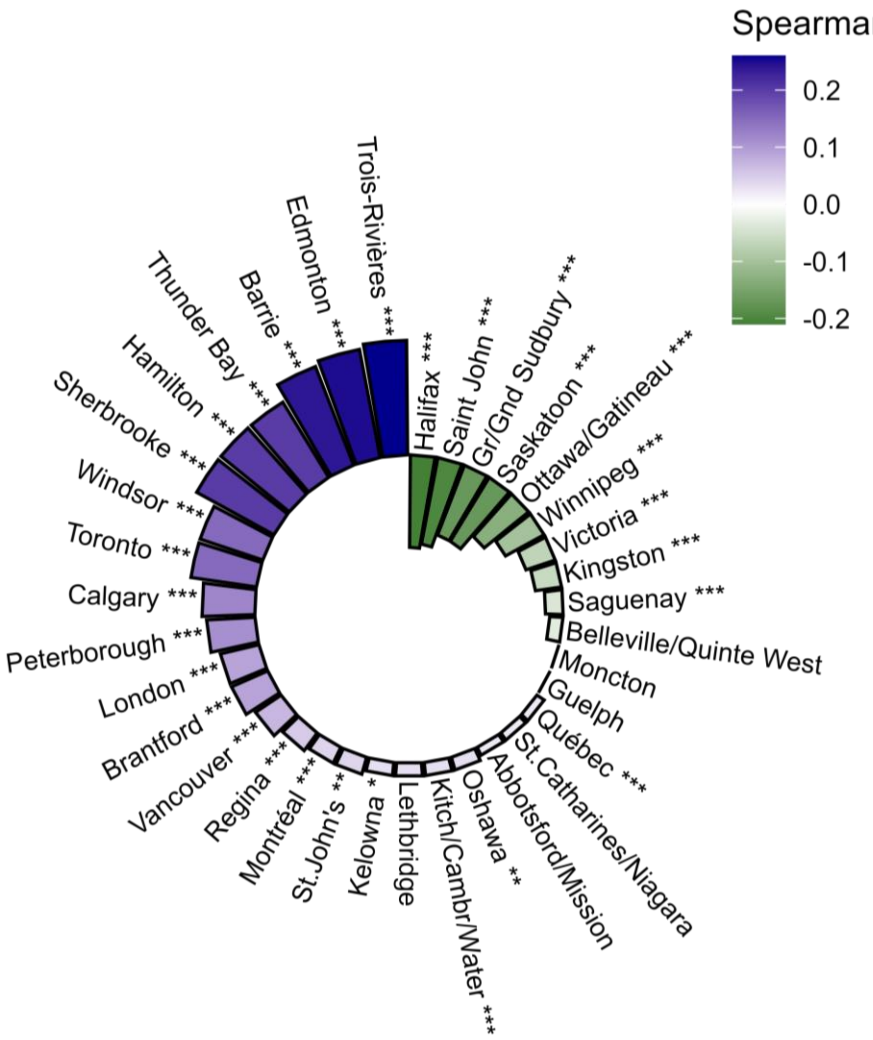

Material advantage is the inverse of the material deprivation index, a widely used metric for assessing material deprivation at the dissemination area in Canada [105]. The 2016 MDI and 2016 dissemination area level geography were used as the 2021 MDI was not available at the time of analysis.

Figure C.2. Within-city correlations between playability scores and small-area level material advantage.

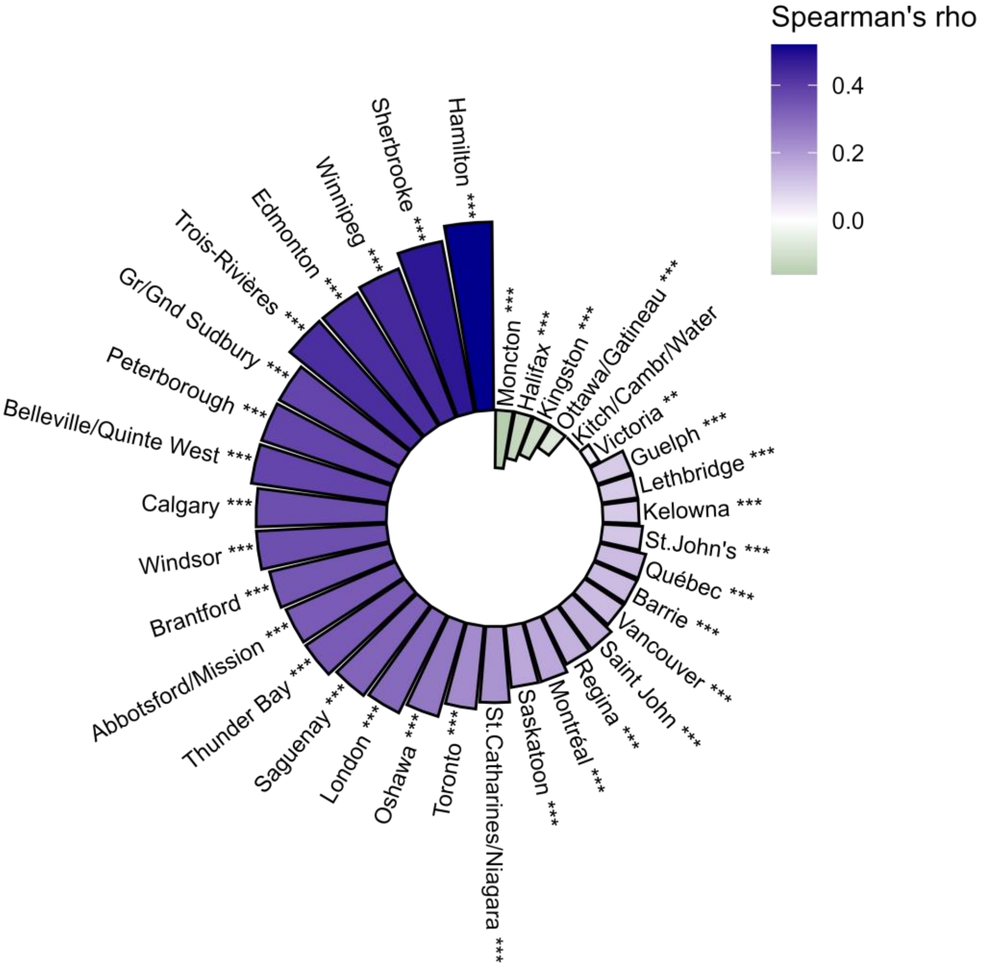

[a] Material advantage is the inverse of the material deprivation index, a widely used metric for assessing material deprivation at the dissemination area in Canada [105]. The 2016 MDI and 2016 dissemination area level geography were used as the 2021 MDI was not available at the time of analysis.

Figure C.3. Within-city correlations between natural environment domain scores and small-area level material advantage.[a]

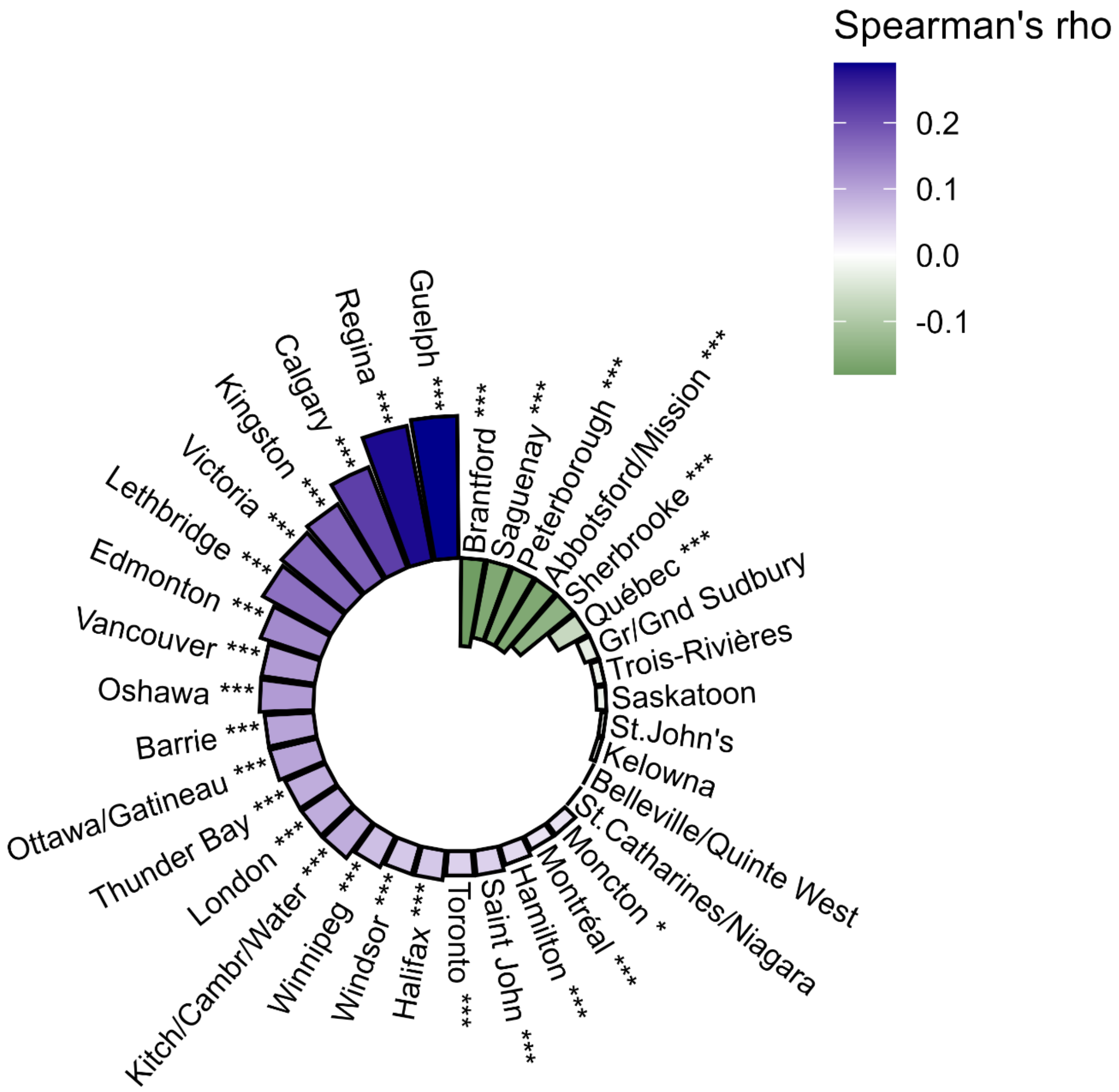

[a]Material advantage is the inverse of the material deprivation index, a widely used metric for assessing material deprivation at the dissemination area in Canada [105]. The 2016 MDI and 2016 dissemination area level geography were used as the 2021 MDI was not available at the time of analysis.

Figure C.4. Within-city correlations between traffic/pedestrian environment scores and small-area level material advantage.[a]

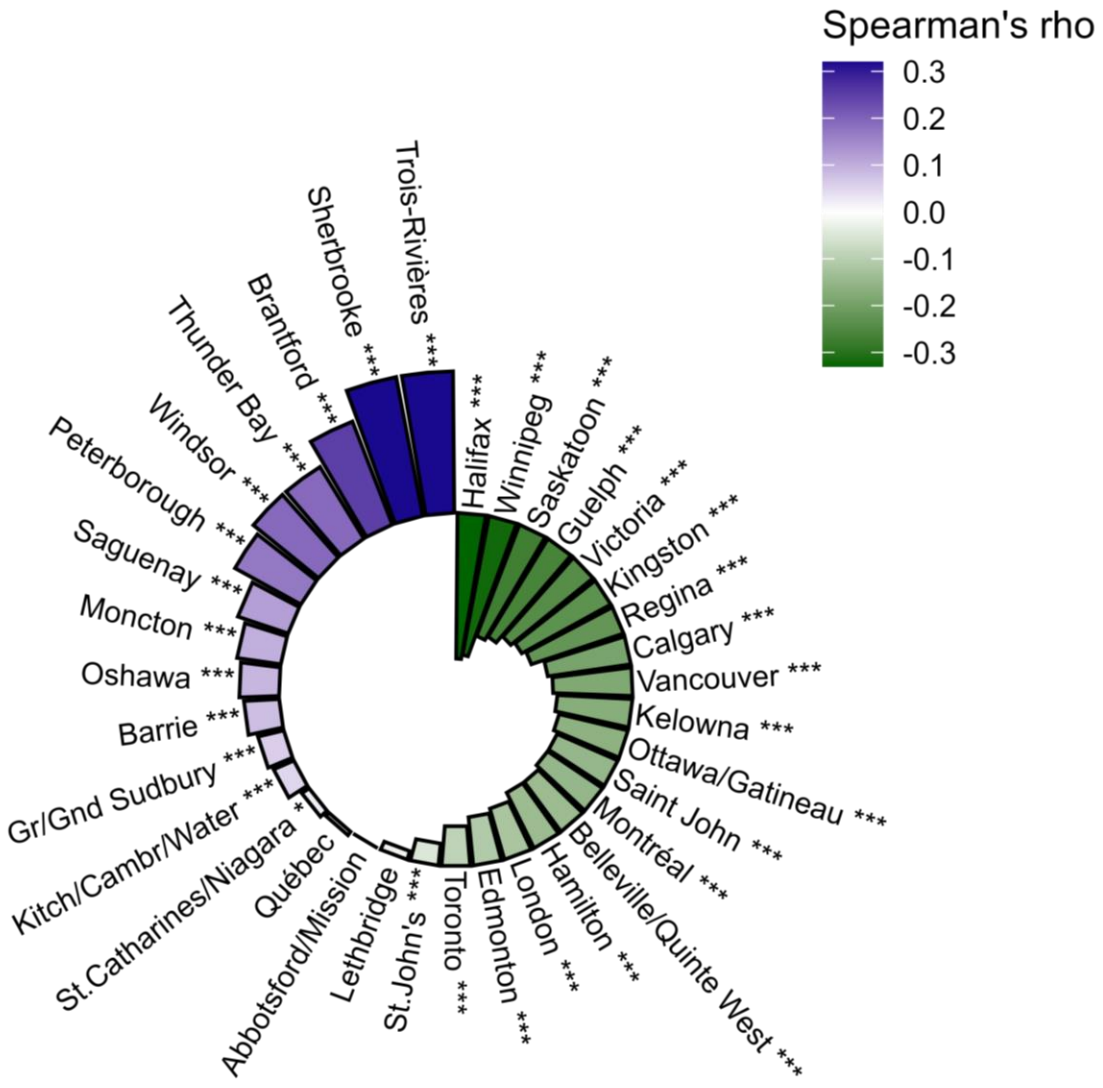

[a] Material advantage is the inverse of the material deprivation index, a widely used metric for assessing material deprivation at the dissemination area in Canada [105]. The 2016 MDI and 2016 dissemination area level geography were used as the 2021 MDI was not available at the time of analysis.

Figure C.5. Within-city correlations between social environment scores and small-area level material advantage.[a]

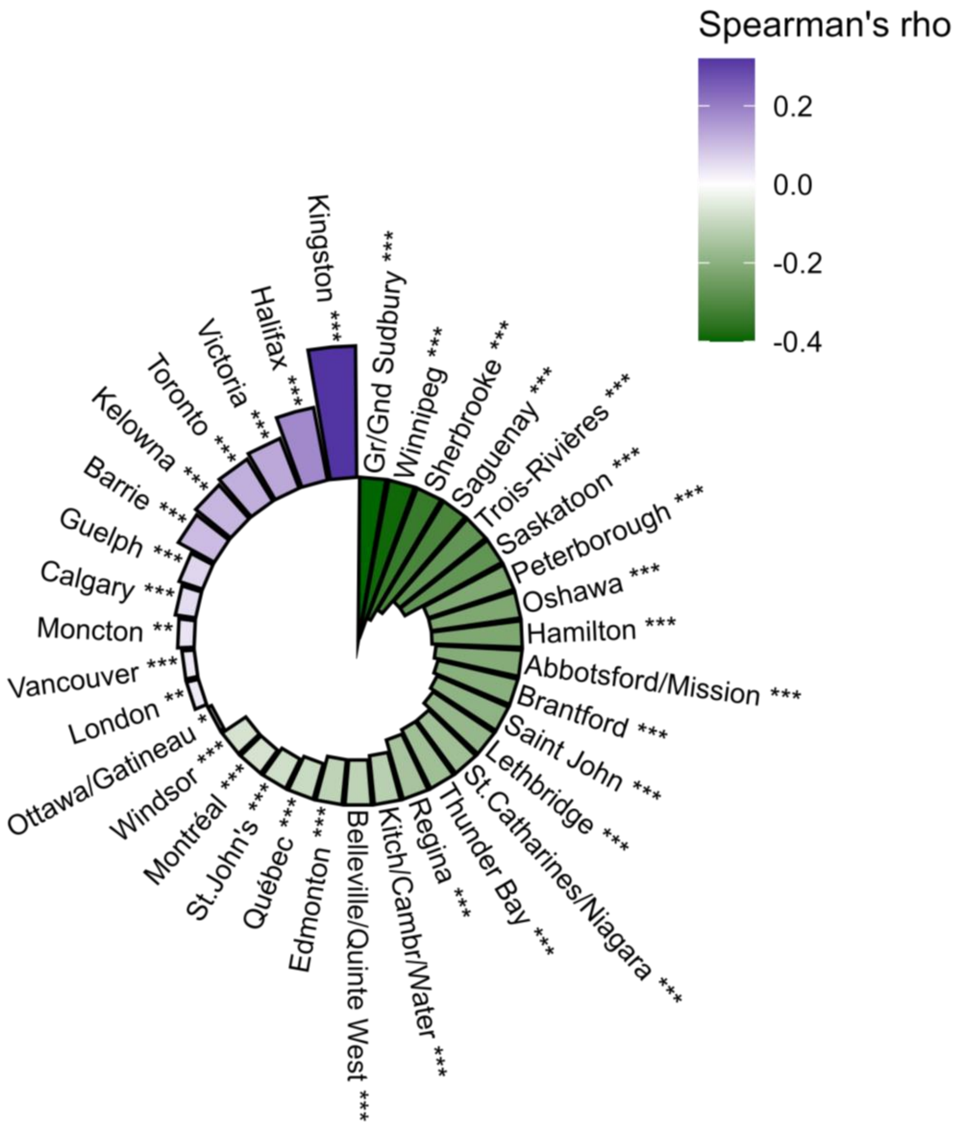

[a.] Material advantage is the inverse of the material deprivation index, a widely used metric for assessing material deprivation at the dissemination area in Canada [105]. The 2016 MDI and 2016 dissemination area level geography were used as the 2021 MDI was not available at the time of analysis.

Figure C.6. Within-city correlations between child-relevant destination domain scores and small-area level material advantage.[a]

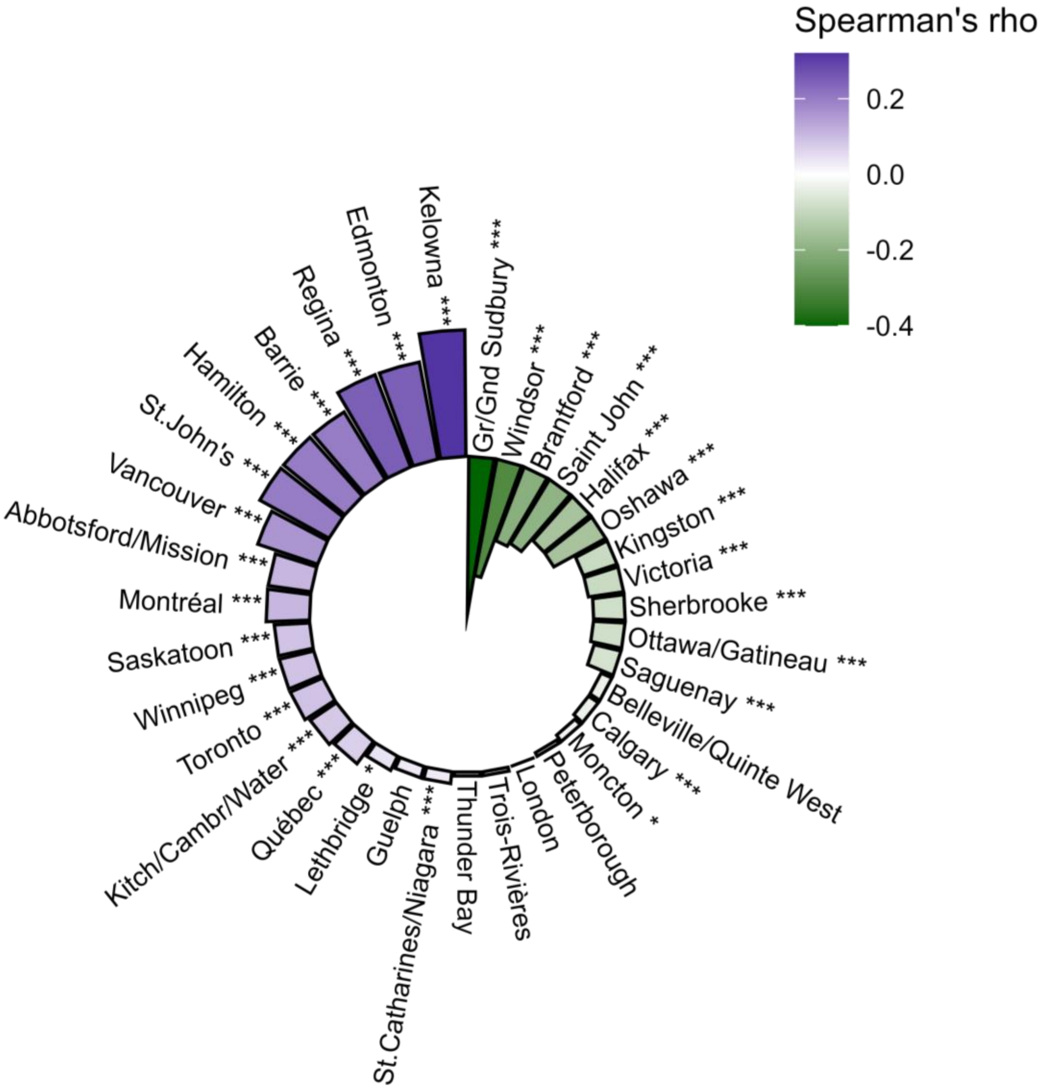

[a] Material advantage is the inverse of the material deprivation index, a widely used metric for assessing material deprivation at the dissemination area in Canada [105]. The 2016 MDI and 2016 dissemination area level geography were used as the 2021 MDI was not available at the time of analysis.

Figure C.7. Within-city correlations between space for play domain scores and small-area level material advantage.[a]

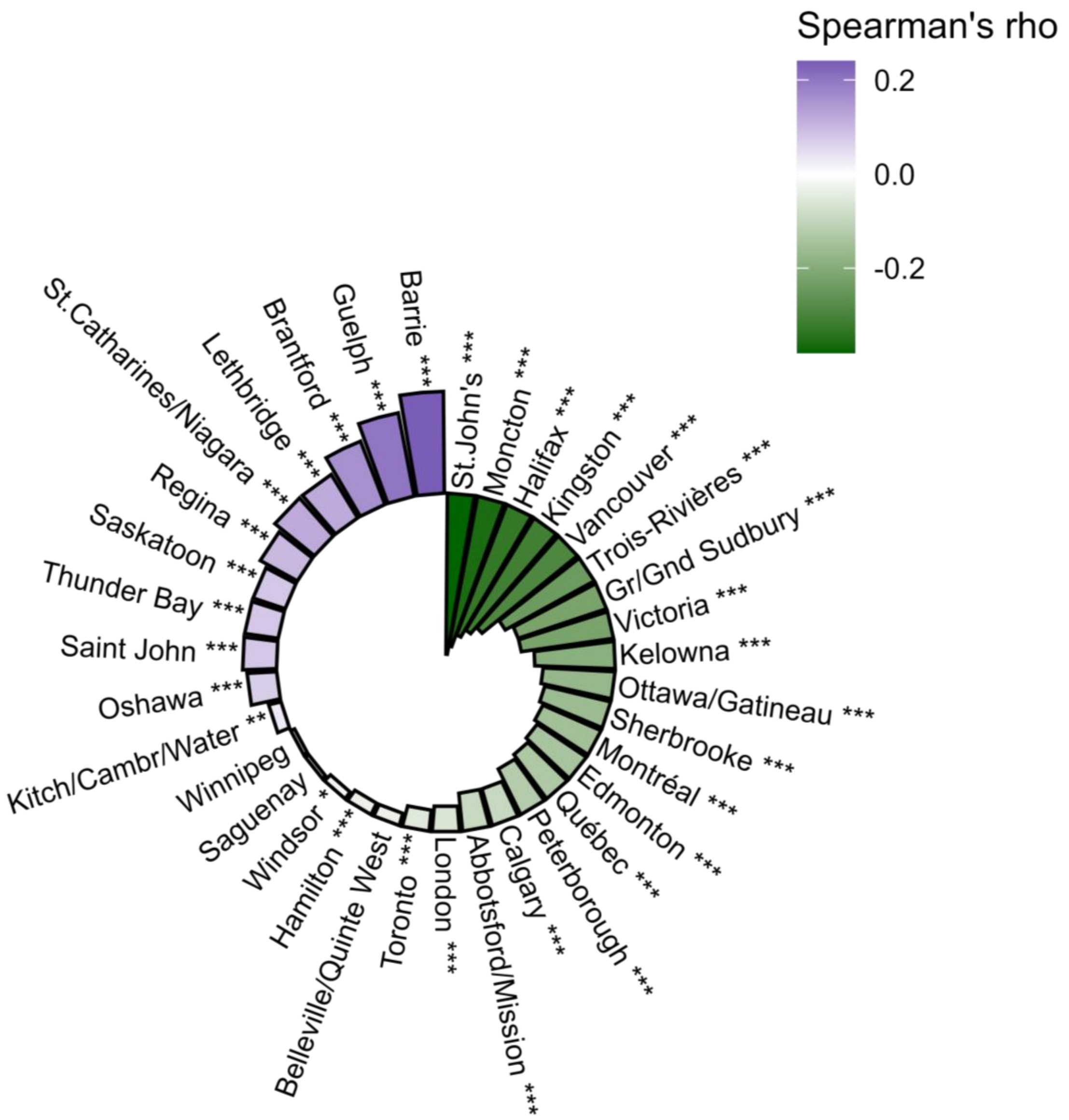

Population density based on 2021 census and dissemination area geography [113]
Significance levels: * = $p < 0.05$; ** = $p < 0.01$; *** = $p < 0.001$

Figure C.8. Within-city correlations between playability and small-area level population density (persons/km2)

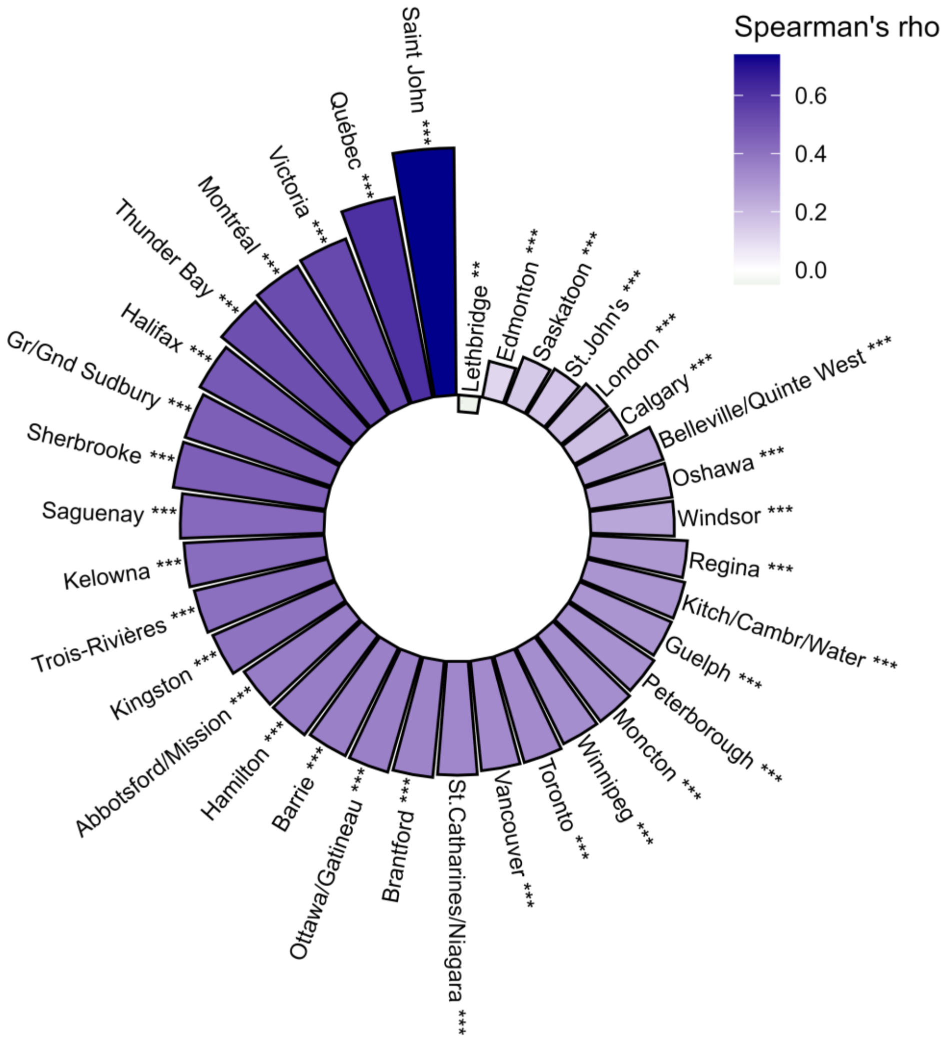

Population density based on 2021 census and dissemination area geography [113]
Significance levels: * = p < 0.05; ** = p < 0.01; *** = p < 0.001

Figure C.9. Within-city correlations between child-relevant destination domain scores and small-area level population density. (persons/km2)

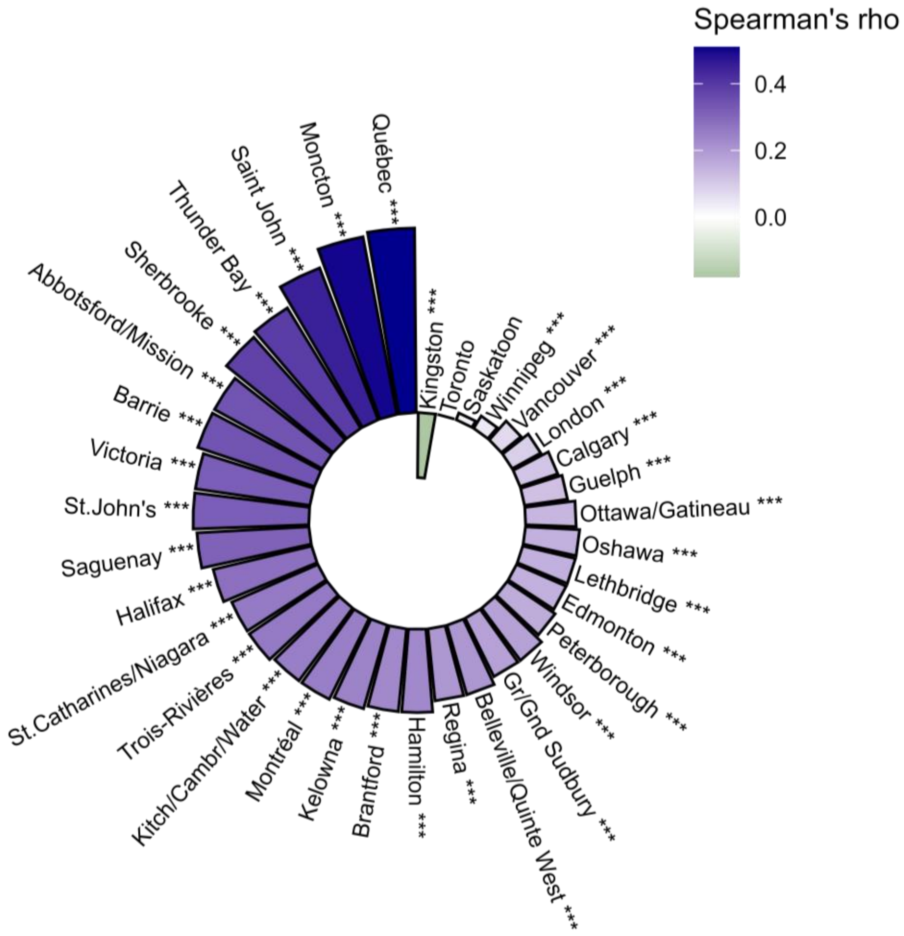

Population density based on 2021 census and dissemination area geography [113]
Significance levels: * = p < 0.05; ** = p < 0.01; *** = p < 0.001

Figure C.10. Within-city correlations between traffic/pedestrian environment domain scores and population density (persons/km2)

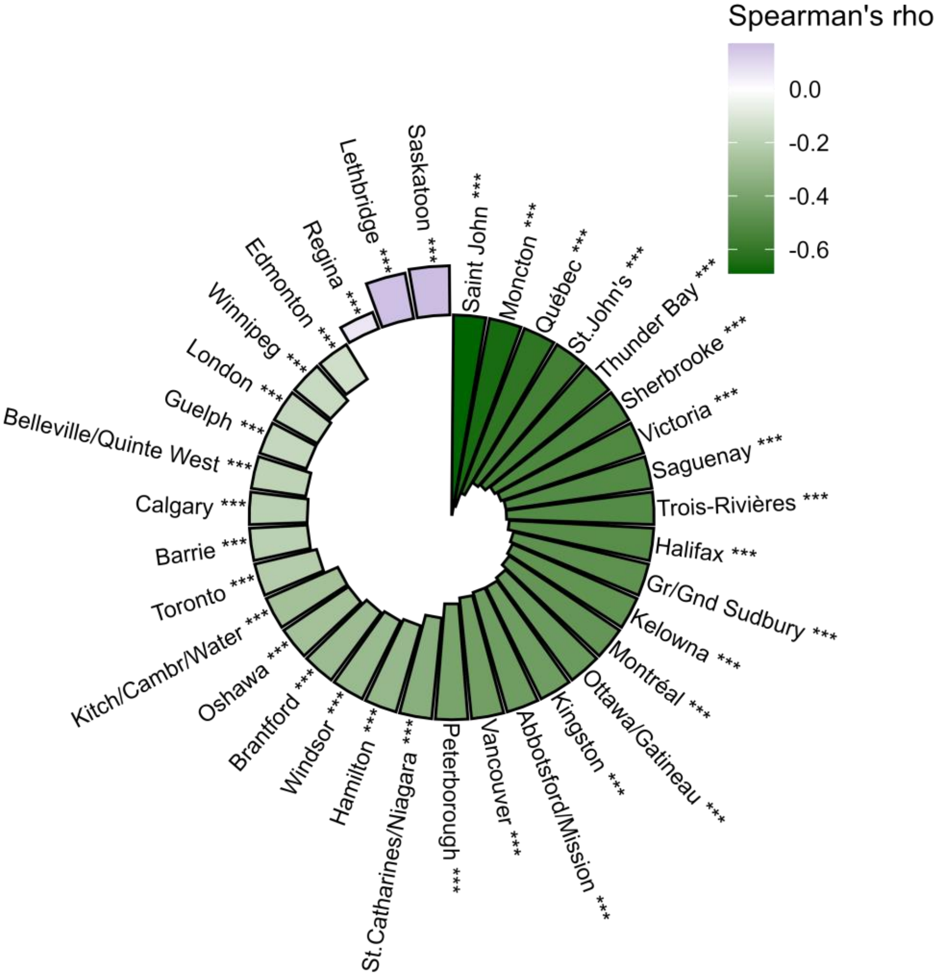

Population density based on 2021 census and dissemination area geography [113]
Significance levels: * = p < 0.05; ** = p < 0.01; *** = p < 0.001

Figure C.11. Within-city correlations between natural environment domain scores and small-area level population density. (persons/km2)

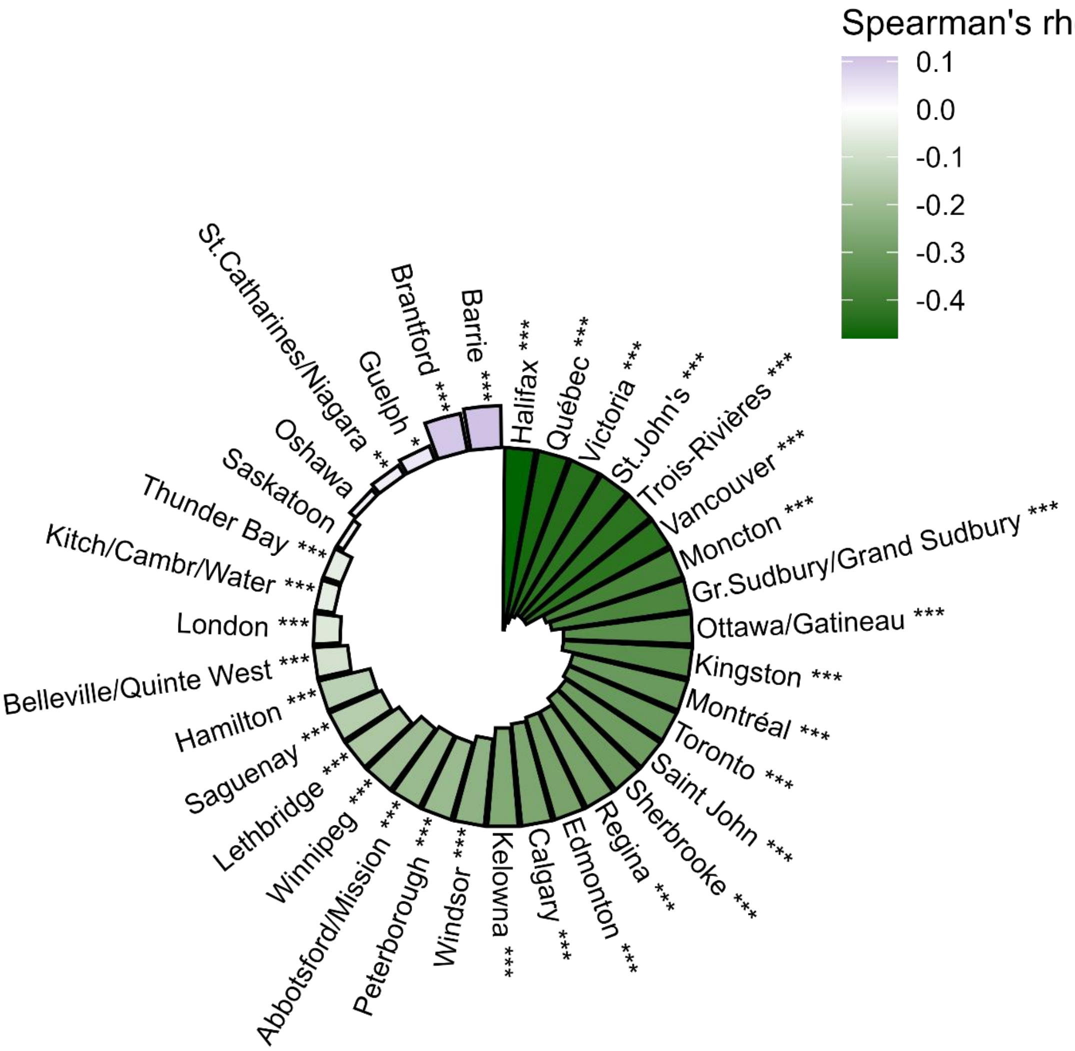

Population density based on 2021 census and dissemination area geography [113]
Significance levels: * = p < 0.05; ** = p < 0.01; *** = p < 0.001

Figure C.12. Within-city correlations between social environment domain scores and small-area level population density. (persons/km2)

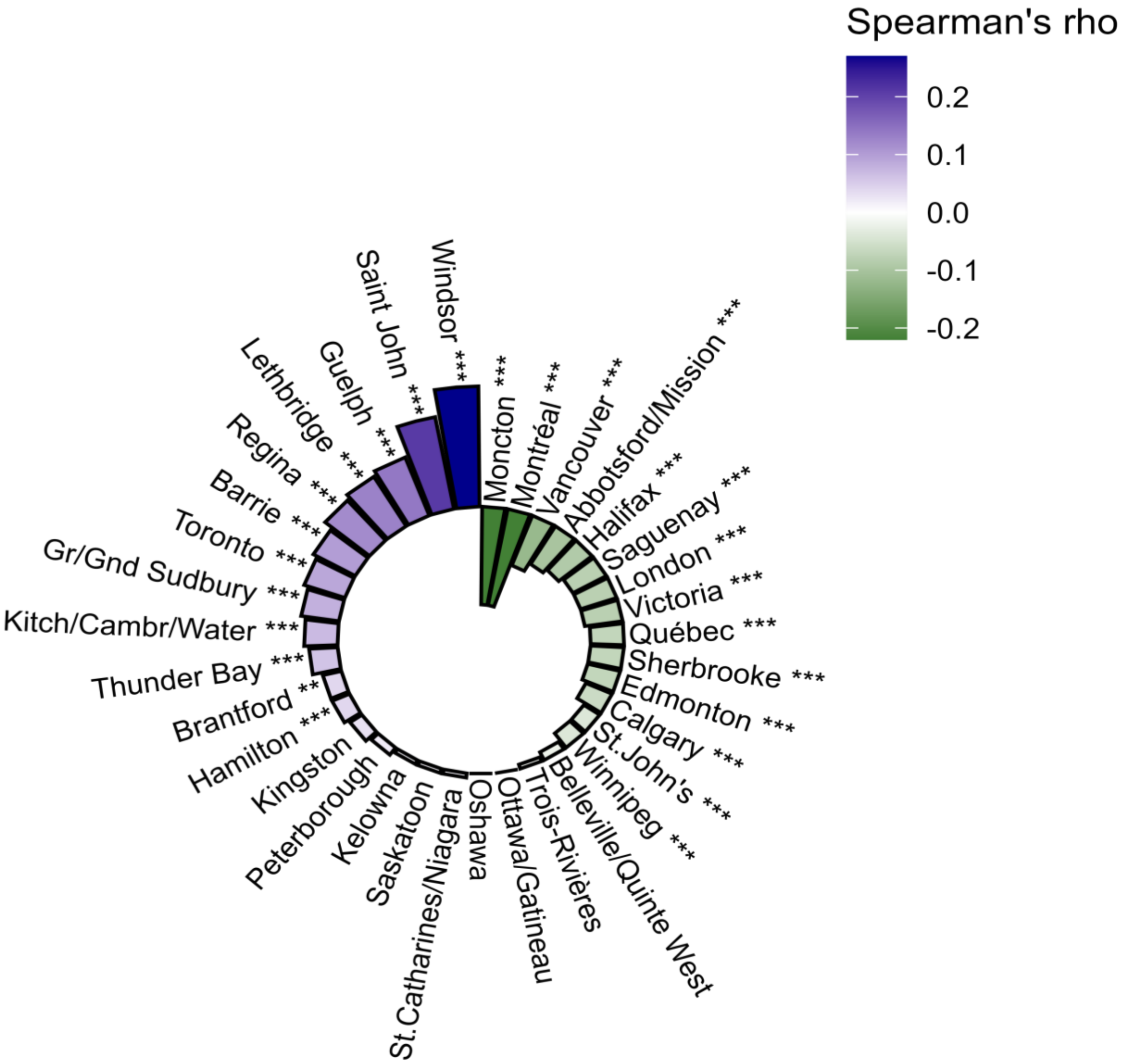

Population density based on 2021 census and dissemination area geography [113]
Significance levels: * = p < 0.05; ** = p < 0.01; *** = p < 0.001

Figure C.13. Within-city correlations between space for play domain scores and small-area level population density (persons/km).

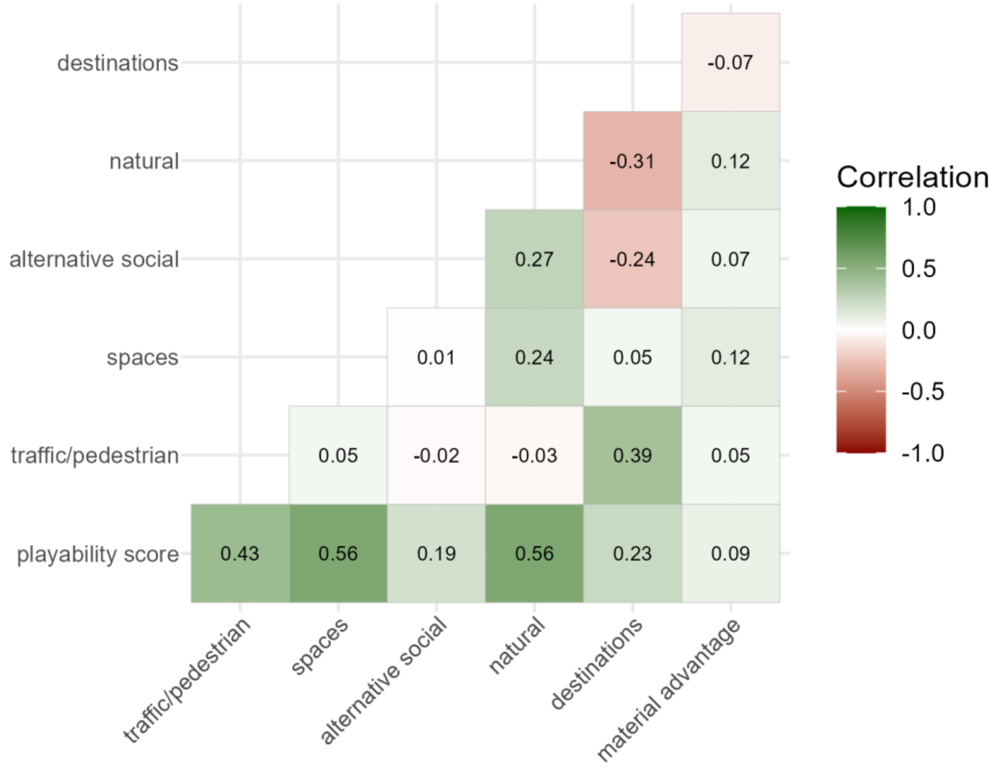

a. All correlations significant at $p < 0.001$

Figure C.14. Correlations between across-city playability and domain scores with alternative measure of social environment, and small-area level material advantage.[a]

Section D. This section presents visualizations for linear and non-linear relationships between DA-level density or material advantage, and postal code-level playability and domain scores for the three largest and smallest cities by population (Figures D.1. – D.24.) and land area (Figures D.25. – D.48.)

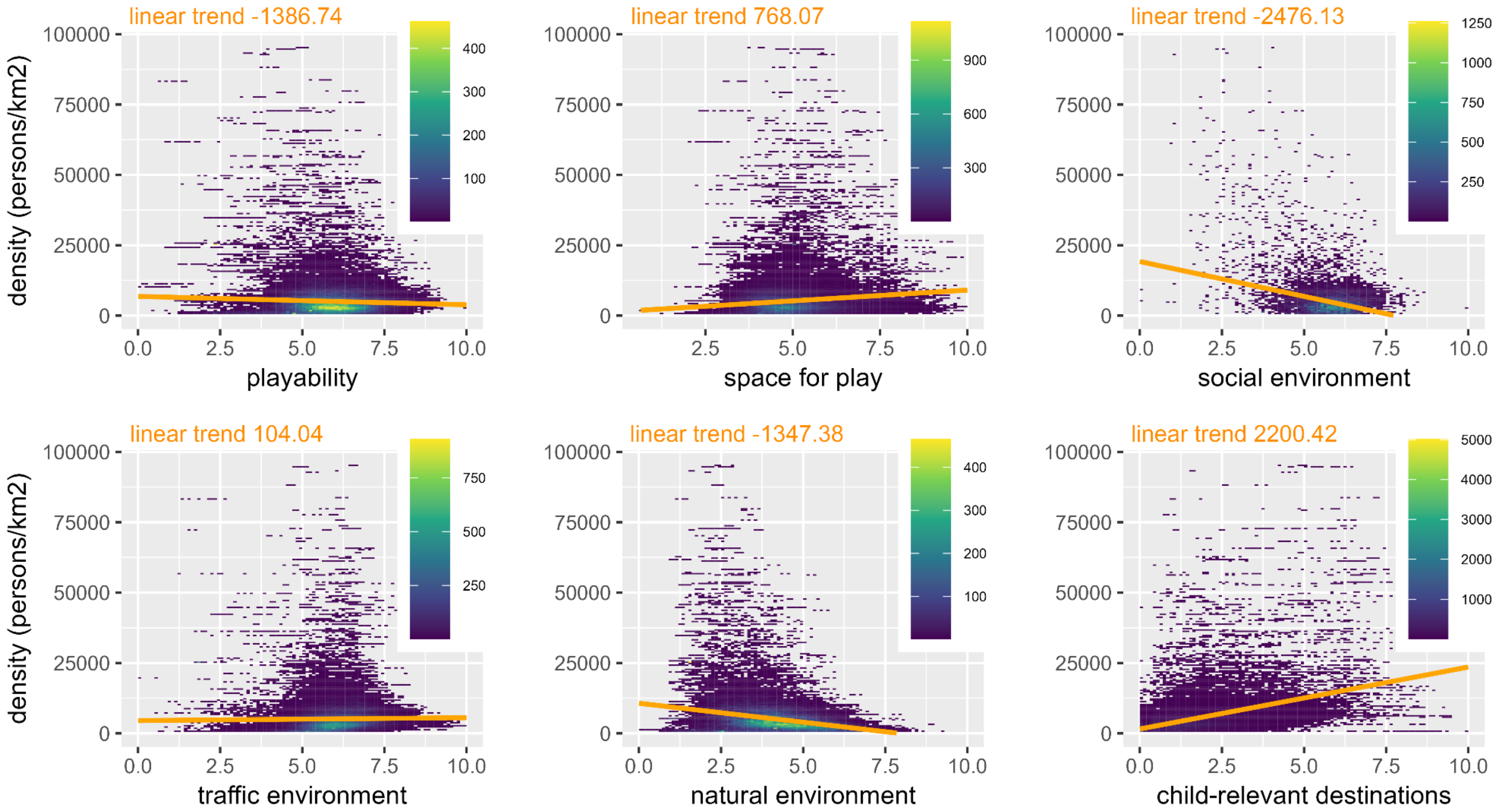

*replaced extreme outliers (values above the 0.05th and 99.95th percentile with 0.05th and 99.95th percentile values to enable visualization.

Figure D.1. Toronto density plots and linear trendlines for relationship between postal code-level playability, domains and population density.*

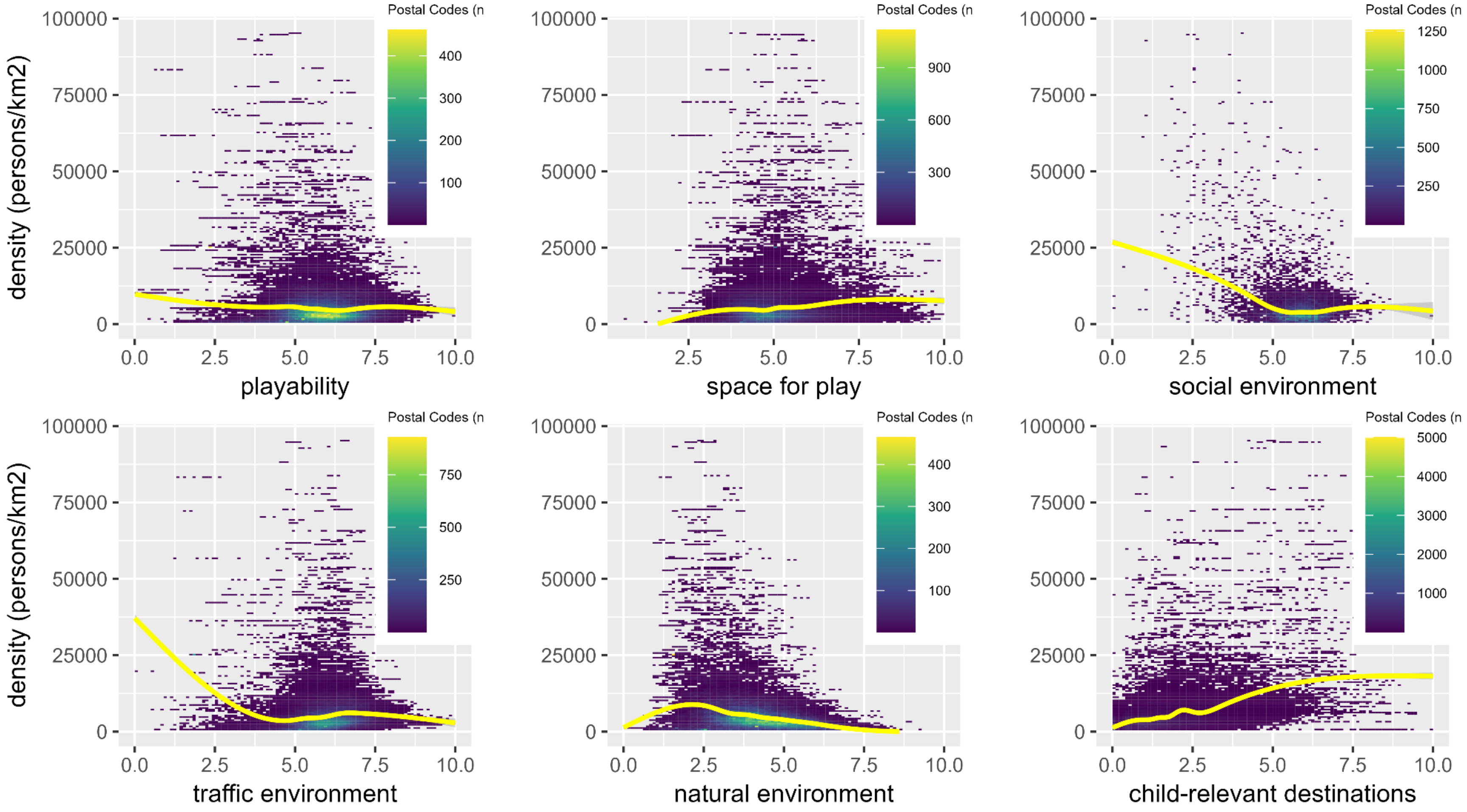

*replaced extreme outliers (replaced values above the 0.05th and 99.95th percentile with 0.05th and 99.95th percentile values to enable visualization

Generalized additive models can be used to obtain a smoothed response function for the relationship between two variables. In this instance, a piecewise cubic function (spline) is used to approximate the relationship between playability, domains and population density.

Figure D.2 Toronto smoothed trendlines (generalized additive models*) for relationships between playability, domains and population density**

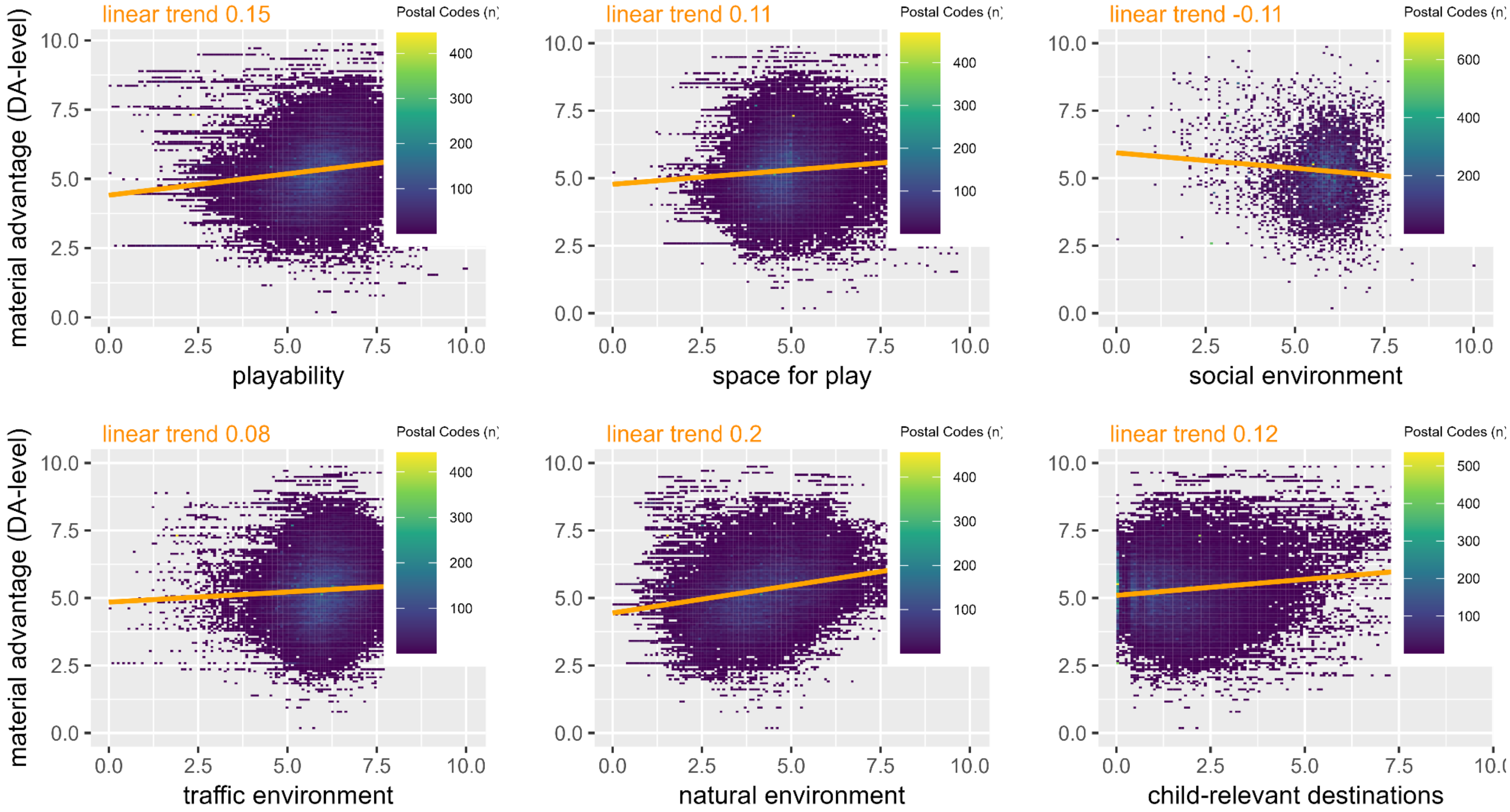

Figure D.3. Toronto density plots and linear trendlines for relationship between postal code-level playability, domains and material advantage.

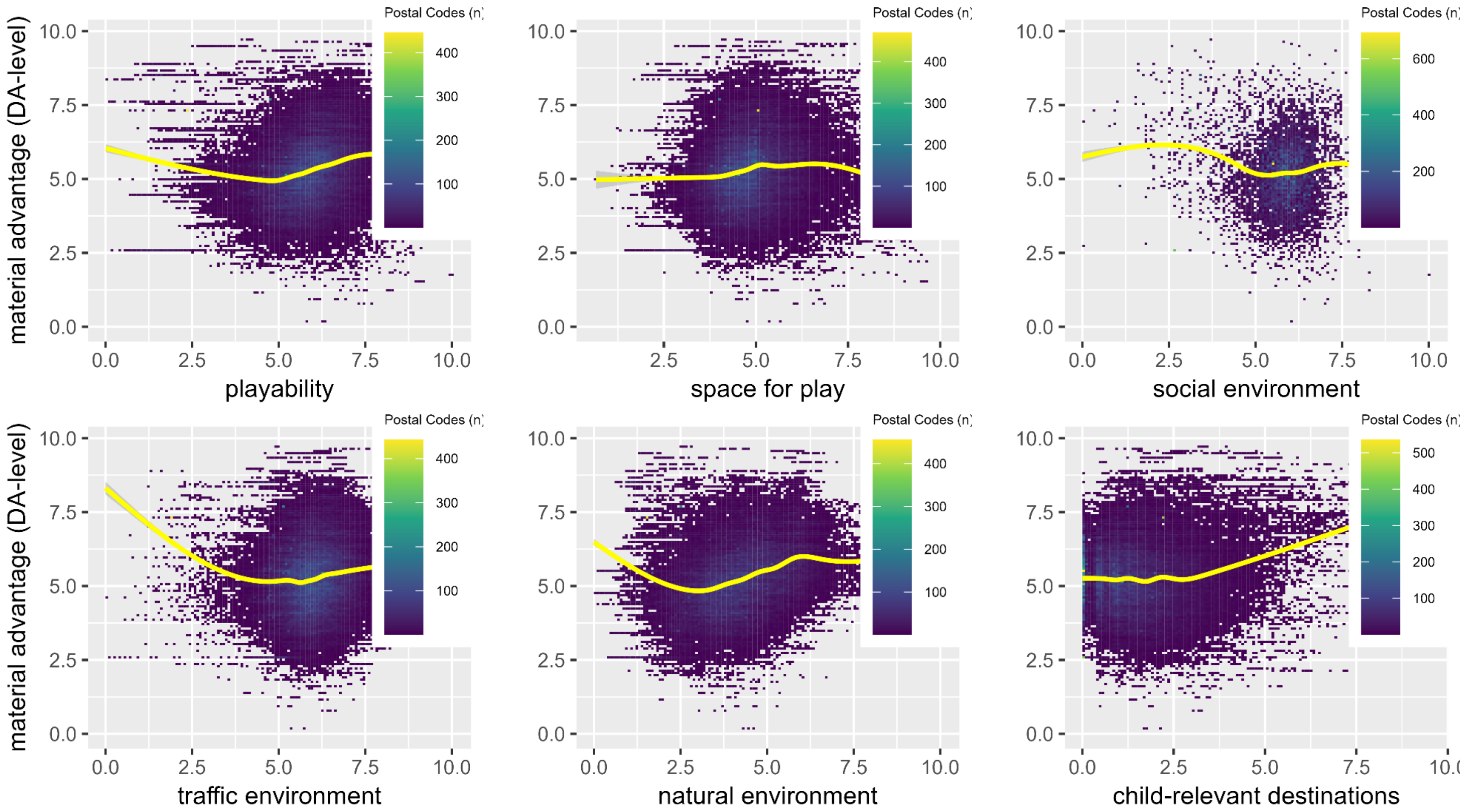

*replaced extreme outliers (values above the 0.05th and 99.95th percentile with 0.05th and 99.95th percentile values to enable visualization
Generalized additive models can be used to obtain a smoothed response function for the relationship between two variables. In this instance, a piecewise cubic function (spline) is used to approximate the relationship between playability, domains and population density.

Figure D.4. Toronto smoothed trendlines (generalized additive models*) for relationships between playability, domains and material advantage.

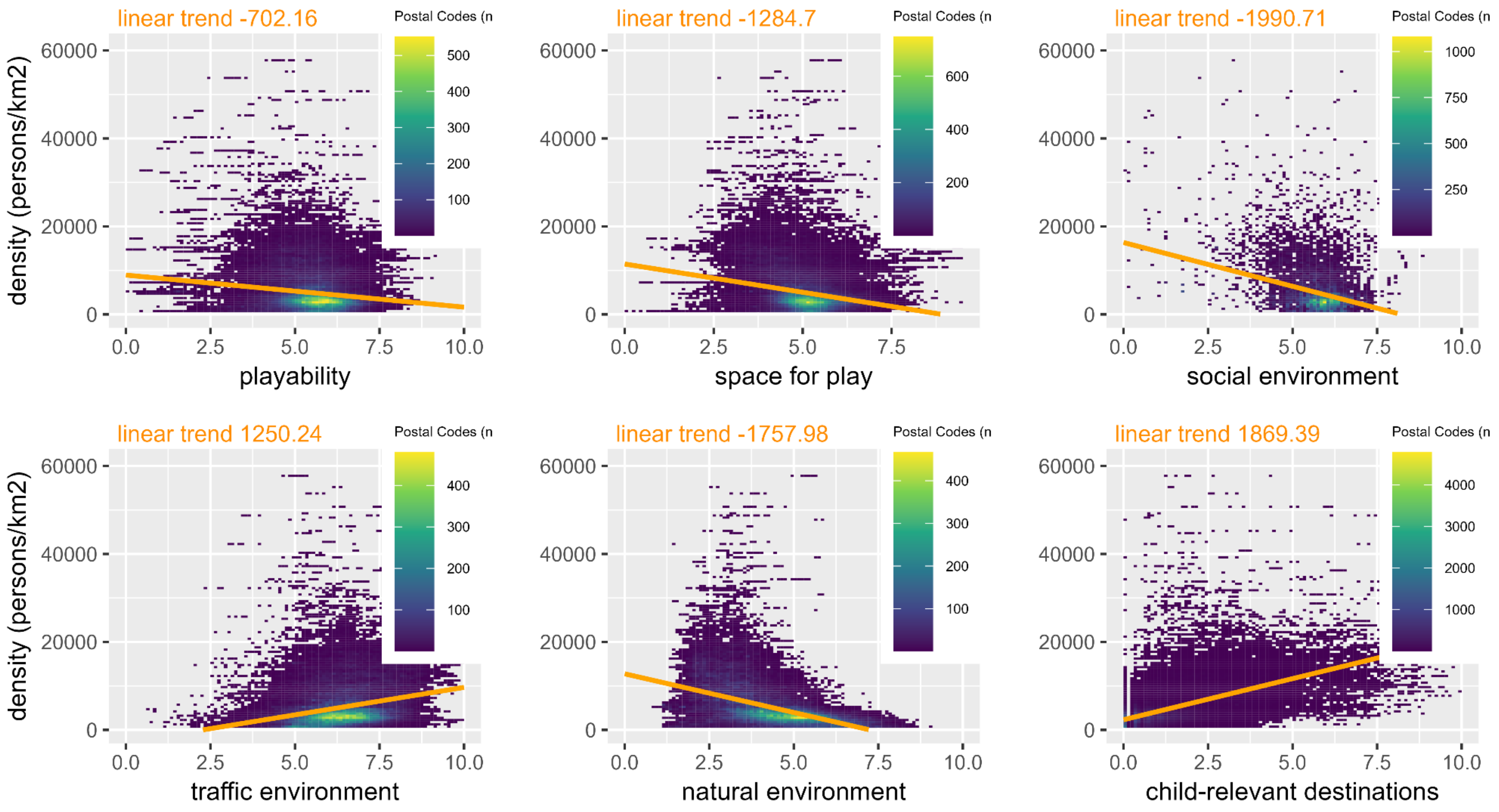

*replaced extreme outliers (values above the 0.05th and 99.95th percentile) with 0.05th and 99.95th percentile values to enable visualization

Figure D.5. Montreal density plots and linear trendlines for relationship between postal code-level playability, domains and population density.*

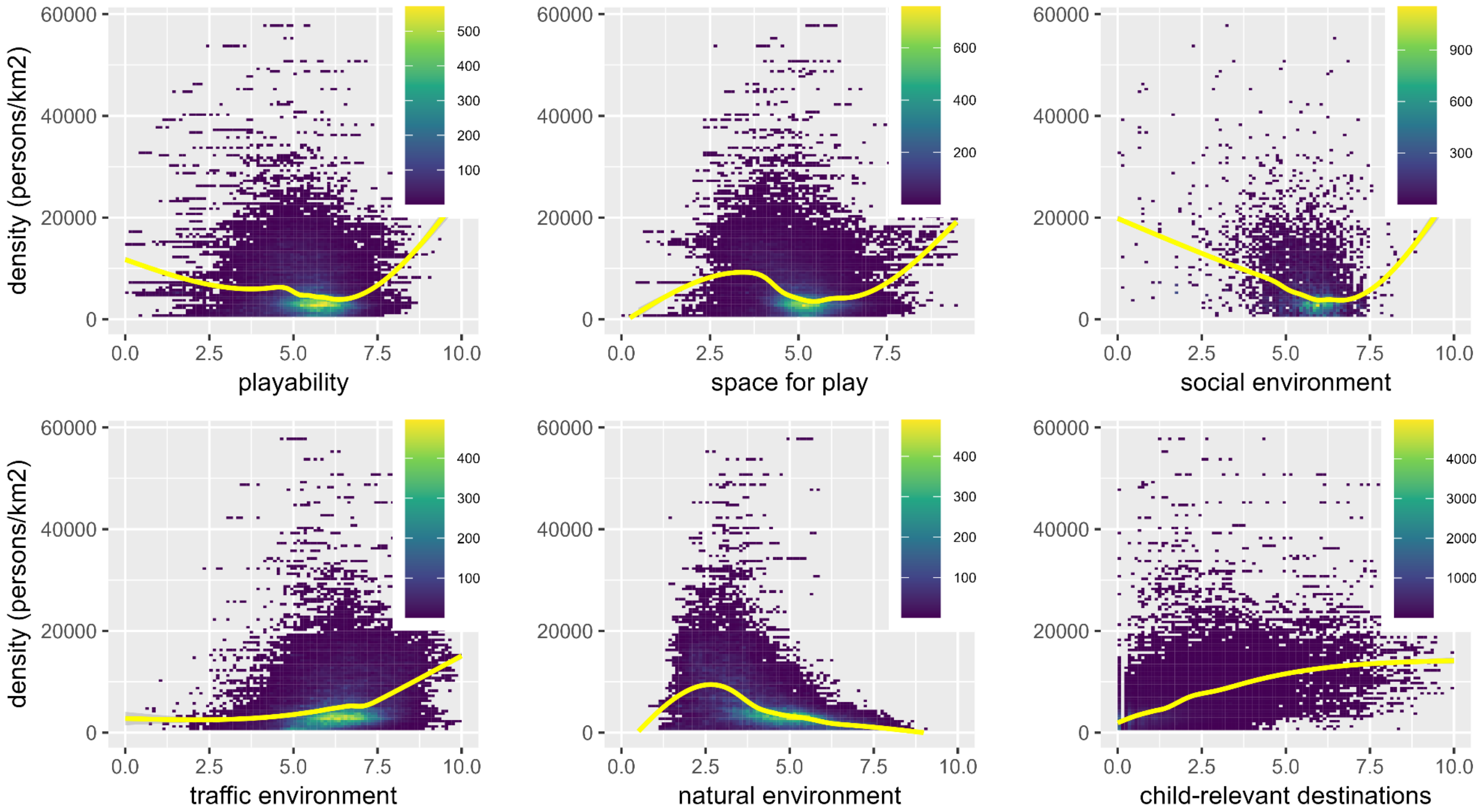

*Generalized additive models can be used to obtain a smoothed response function for the relationship between two variables. In this instance, a piecewise cubic function (spline) is used to approximate the relationship between playability, domains and population density.

**replaced extreme outliers (values above the 0.05th and 99.95th percentile) with 0.05th and 99.95th percentile values to enable visualization

Figure D.6. Montreal smoothed trendlines (generalized additive models) for relationship between postal code-level playability, domains and population density.*

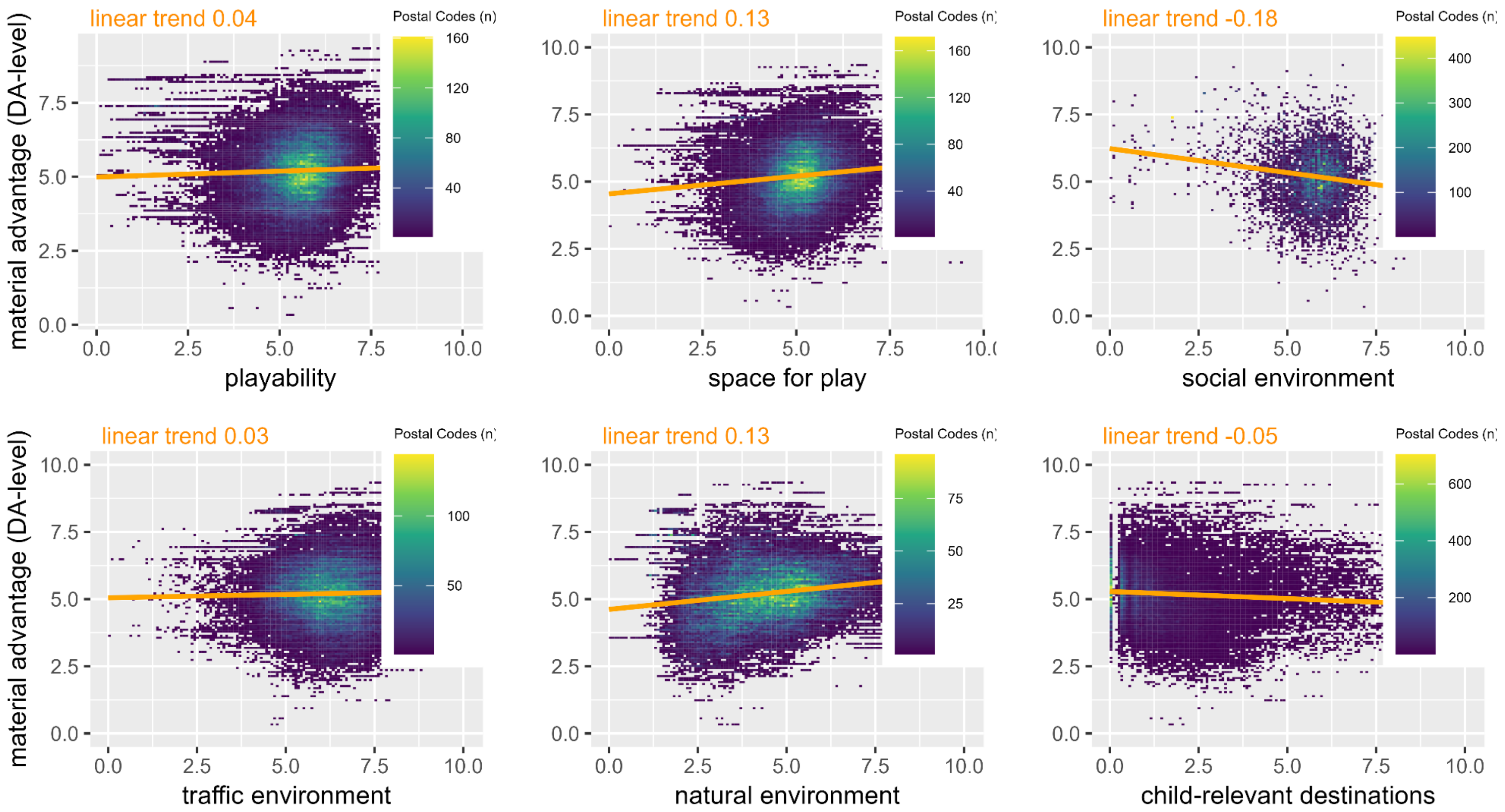

Figure D.7. Montreal density plots and linear trendlines for relationship between postal code-level playability, domains and material advantage.

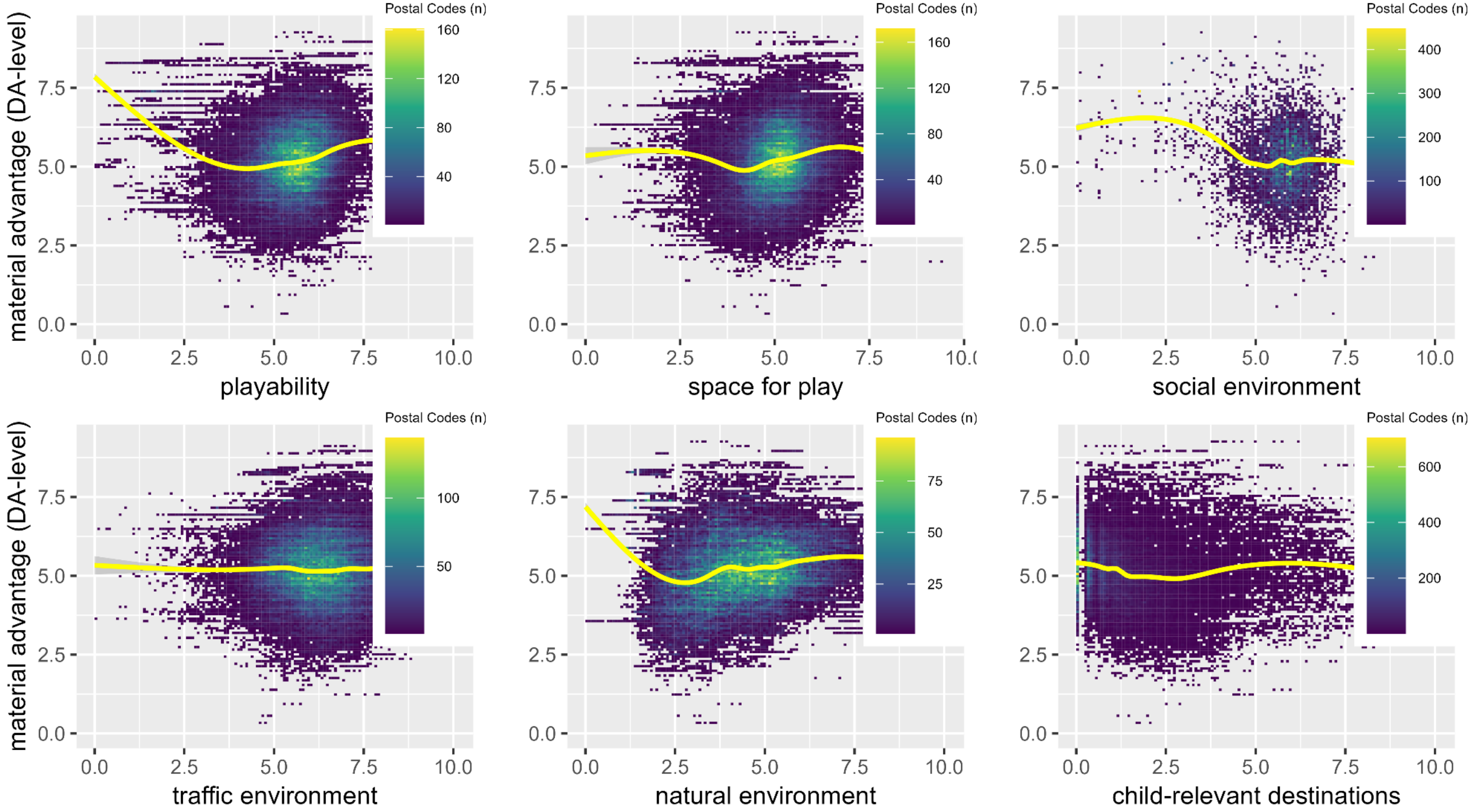

Generalized additive models can be used to obtain a smoothed response function for the relationship between two variables. In this instance, a piecewise cubic function (spline) is used to approximate the relationship between playability, domains and population density.

Figure D.8. Montreal smoothed trendlines (generalized additive models*) for relationships between playability, domains and material advantage.

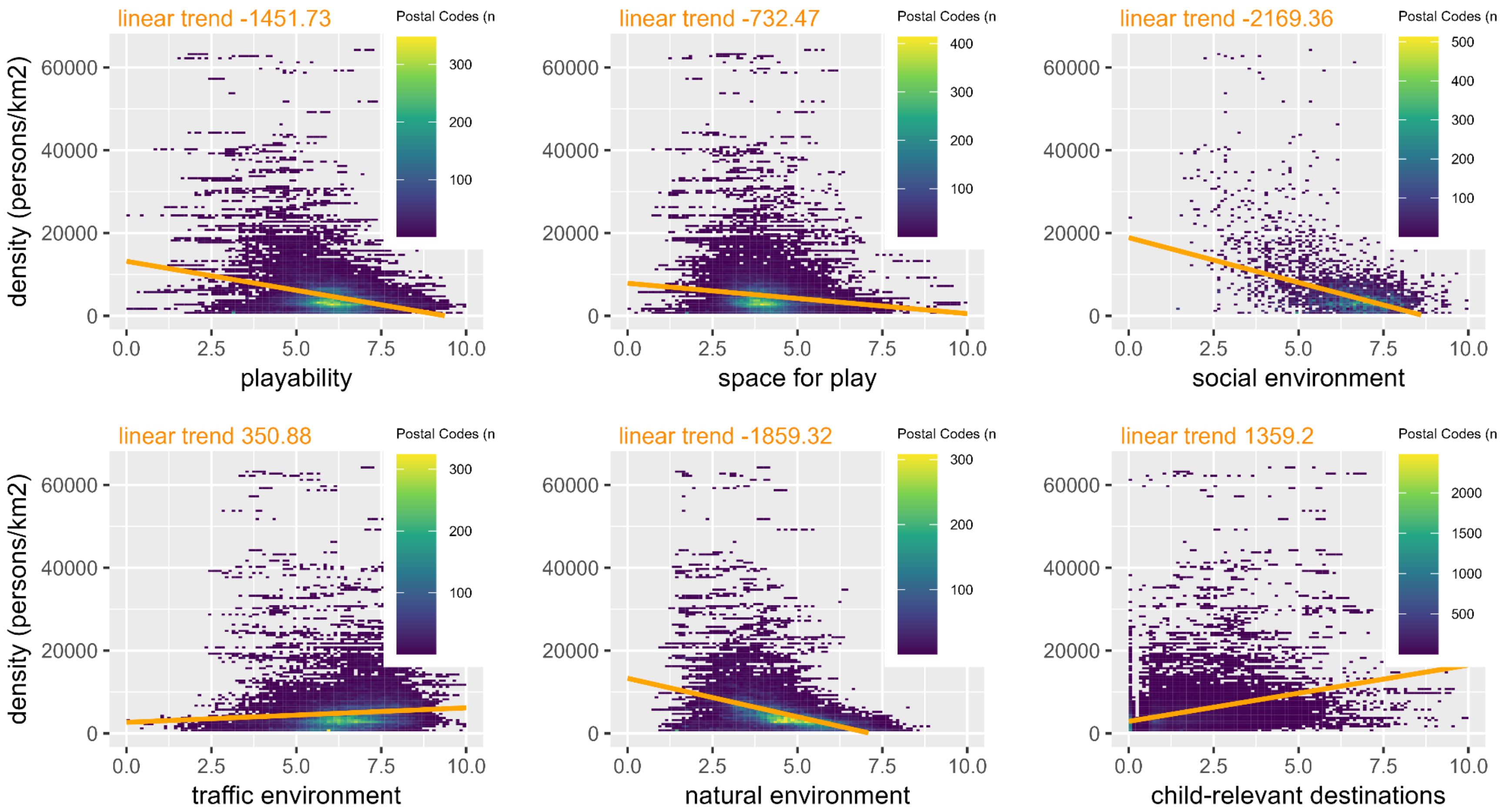

*replaced extreme outliers (values above the 0.05th and 99.95th percentile) with 0.05th and 99.95th percentile values to enable visualization

Figure D.9. Vancouver density plots and trendlines for relationship between postal code-level playability, domains and population density*

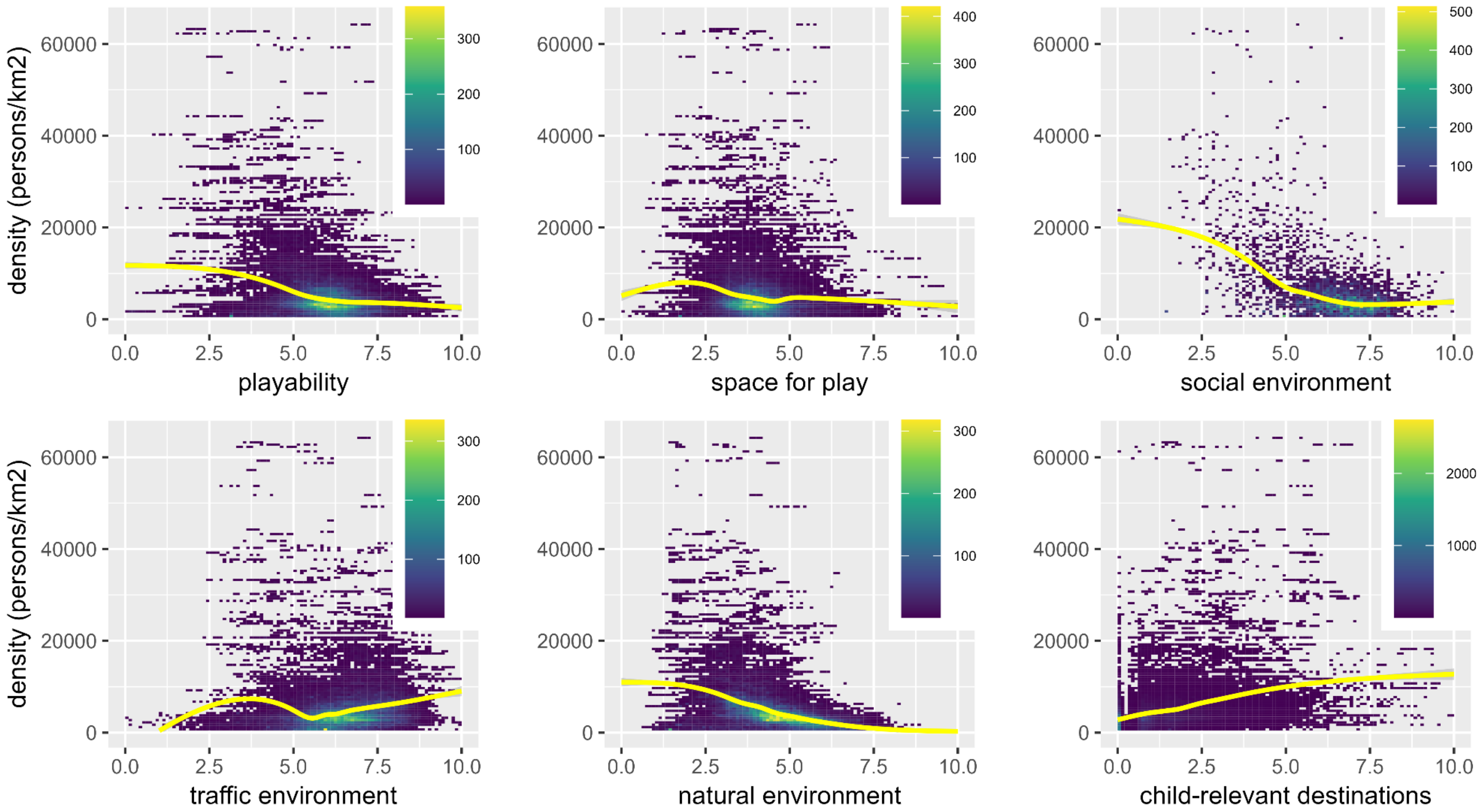

*Generalized additive models can be used to obtain a smoothed response function for the relationship between two variables. In this instance, a piecewise cubic function (spline) is used to approximate the relationship between playability, domains and population density.

**replaced extreme outliers (values above the 0.05th and 99.95th percentile) with 0.05th and 99.95th percentile values to enable visualization

Figure D.10. Vancouver smoothed trendlines (generalized additive models*) for relationship between postal code-level playability, domains and population density**

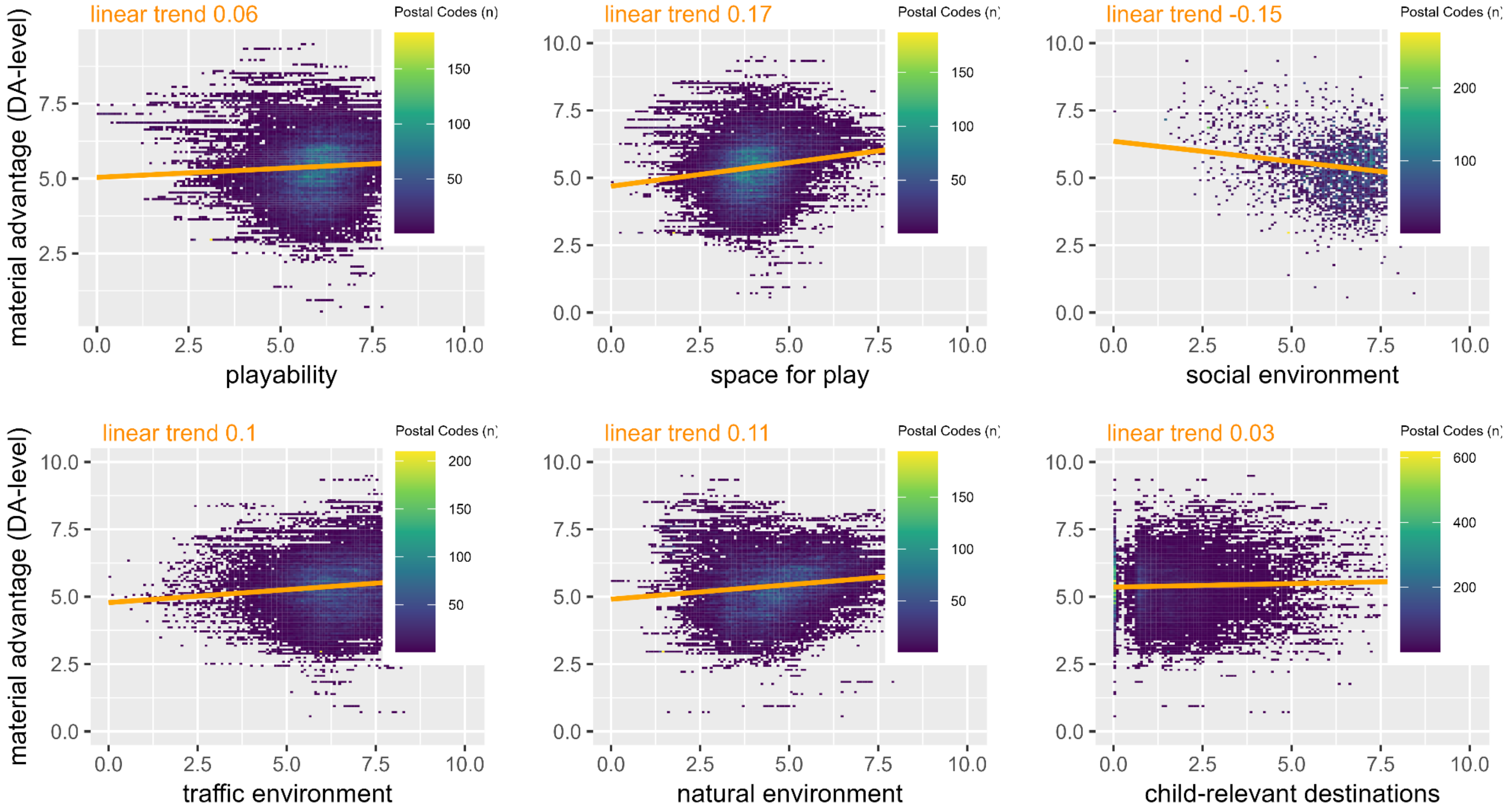

Figure D.11. Vancouver density plots and linear trendlines for relationship between postal code-level playability, domains and material advantage

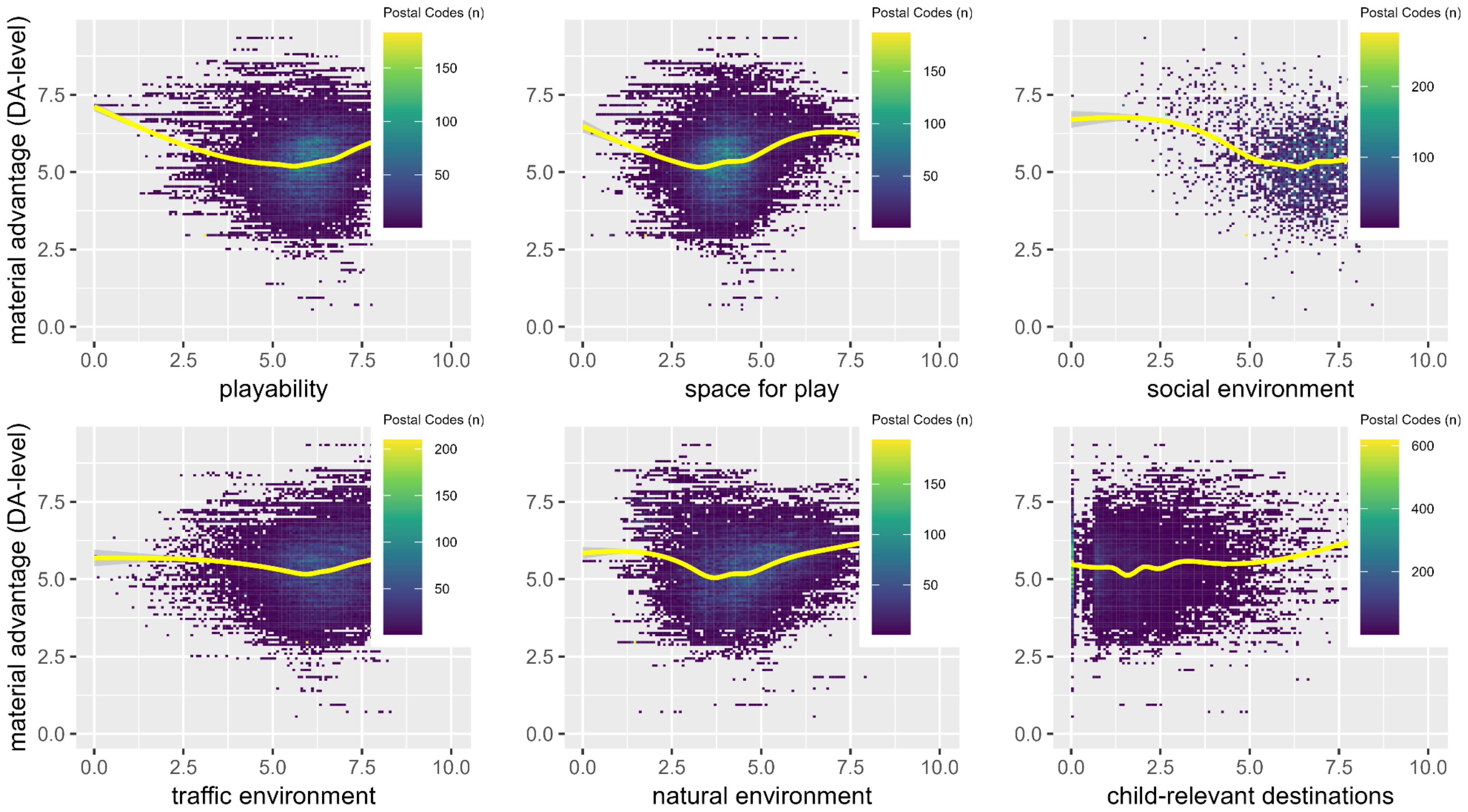

Generalized additive models can be used to obtain a smoothed response function for the relationship between two variables. In this instance, a piecewise cubic function (spline) is used to approximate the relationship between playability, domains and population density.

Figure D.12. Vancouver smoothed trendlines (generalized additive models*) for relationships between playability, domains and material advantage.

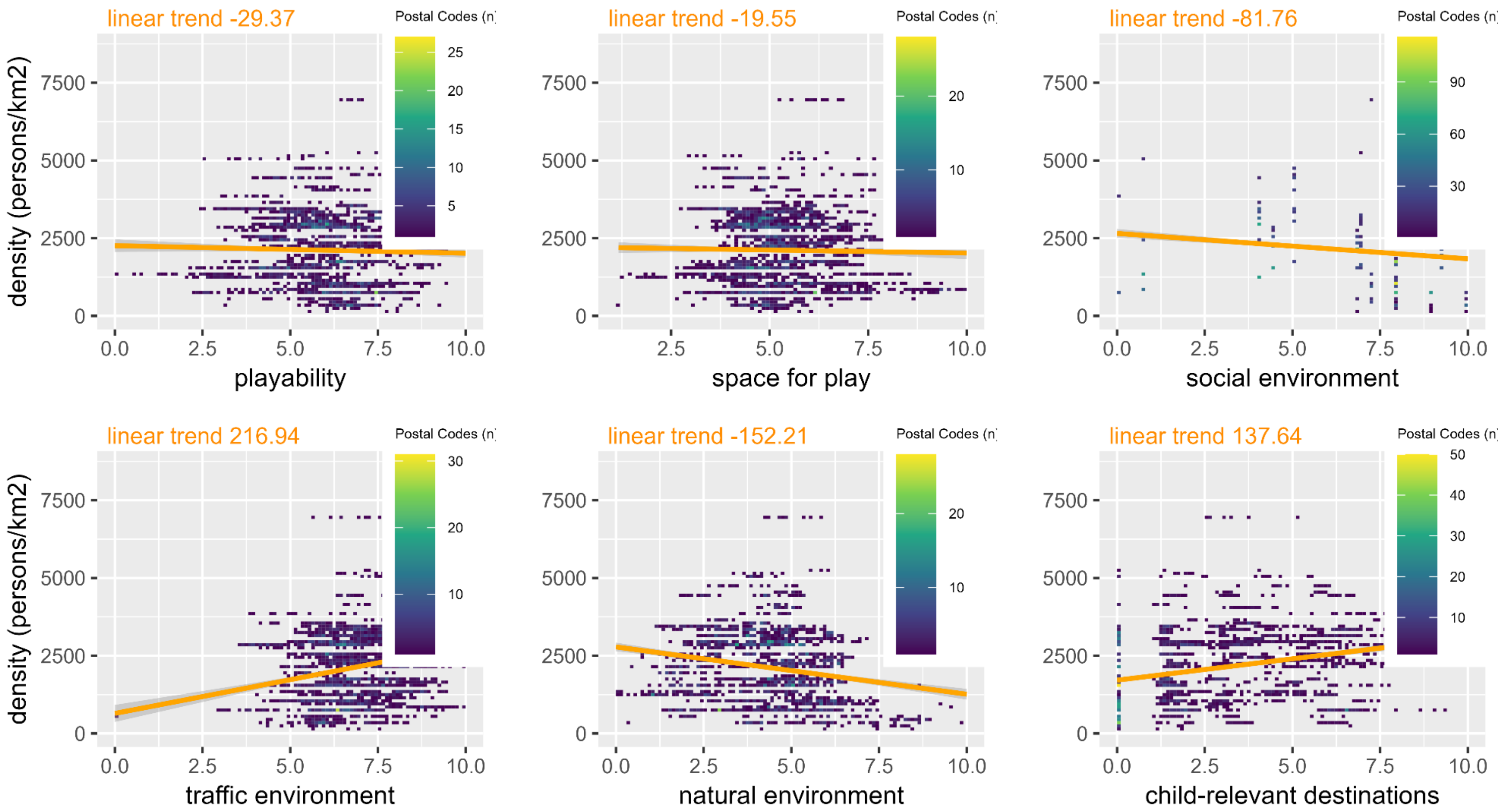

*replaced extreme outliers (values above the 0.05th and 99.95th percentile) with 0.05th and 99.95th percentile values to enable visualization

Figure D.13. Belleville - Quinte West density plots and trendlines for relationship between postal code-level playability, domains and population density*

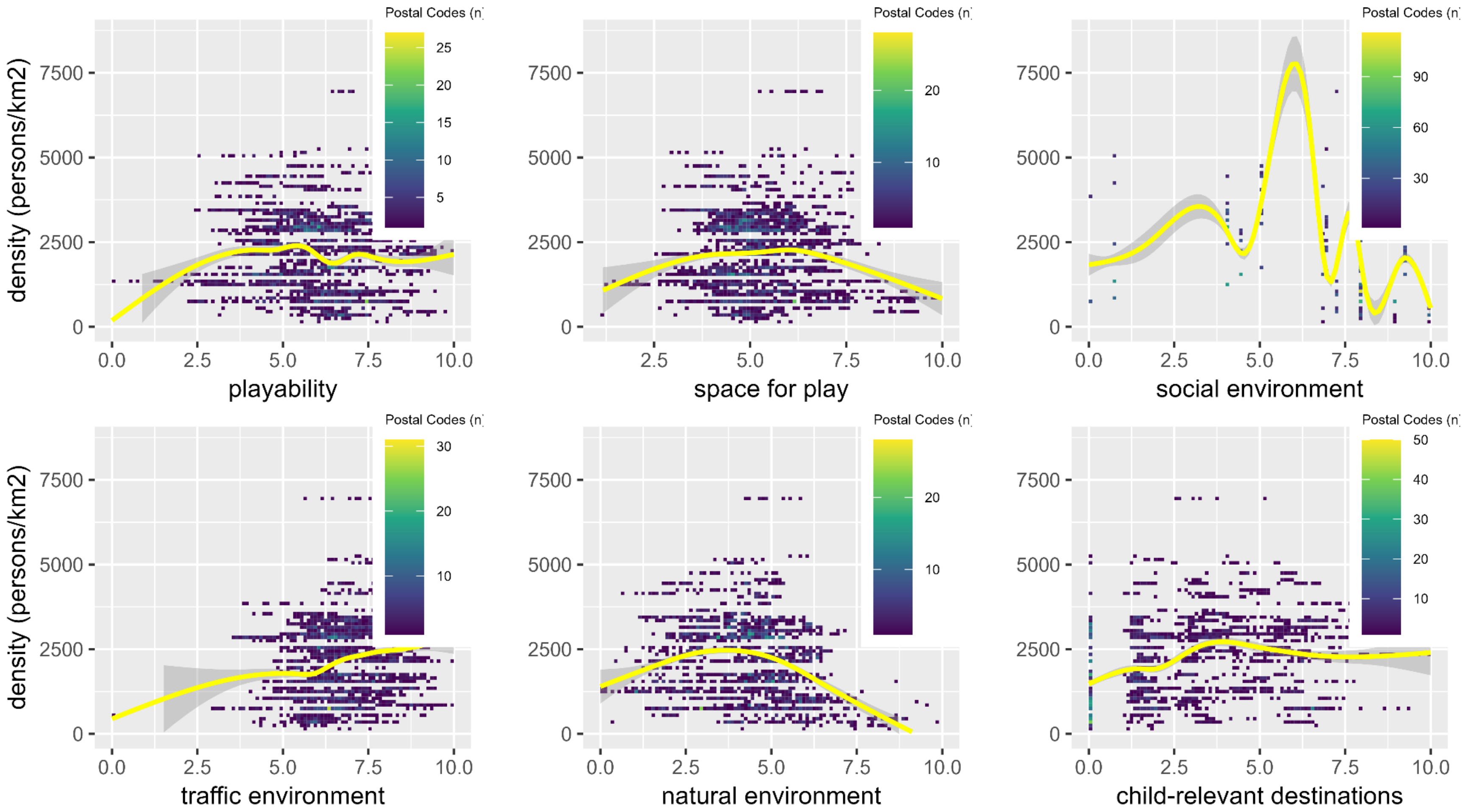

*Generalized additive models can be used to obtain a smoothed response function for the relationship between two variables. In this instance, a piecewise cubic function (spline) is used to approximate the relationship between playability, domains and population density.

**replaced extreme outliers (values above the 0.05th and 99.95th percentile) with 0.05th and 99.95th percentile values to enable visualization

Figure D.14. Belleville - Quinte West smoothed trendlines (generalized additive models*) for relationship between postal code-level playability, domains and population density**

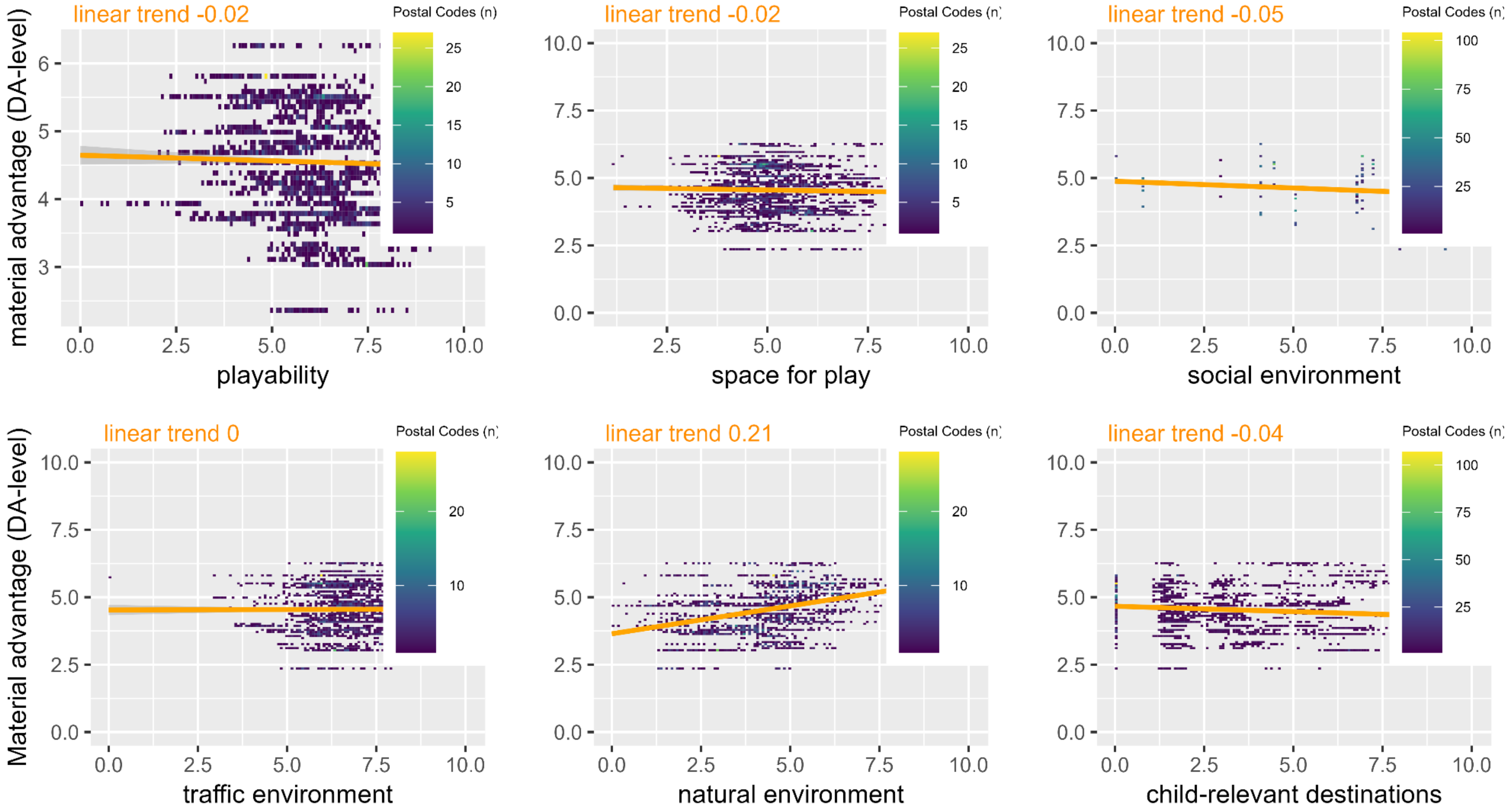

Figure D.15. Belleville – Quinte West density plots and linear trendlines for relationship between postal code-level playability, domains and material advantage.

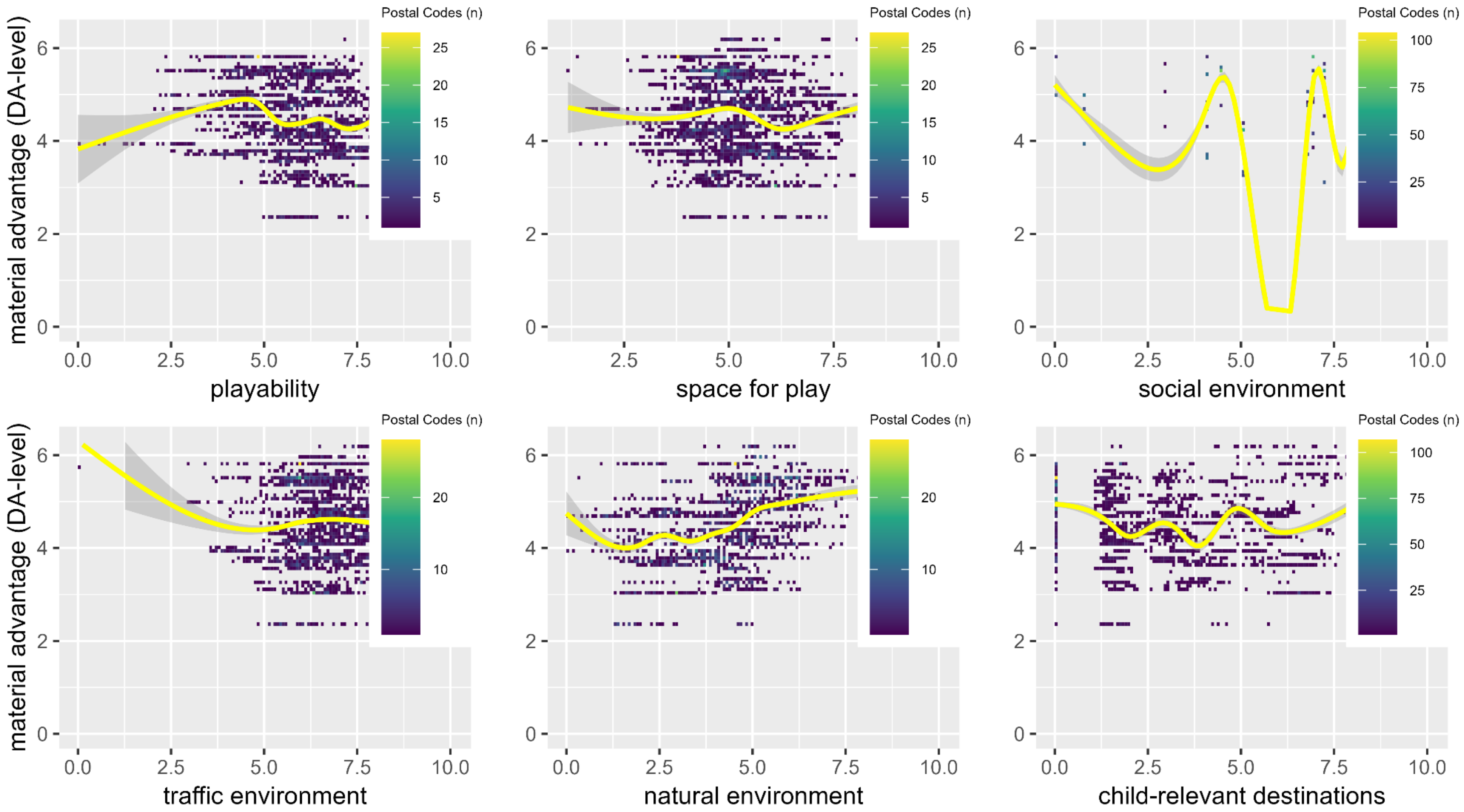

Generalized additive models can be used to obtain a smoothed response function for the relationship between two variables. In this instance, a piecewise cubic function (spline) is used to approximate the relationship between playability, domains and population density.

Figure D.16. Belleville – Quinte West smoothed trendlines (generalized additive models*) for relationships between playability, domains and material advantage.

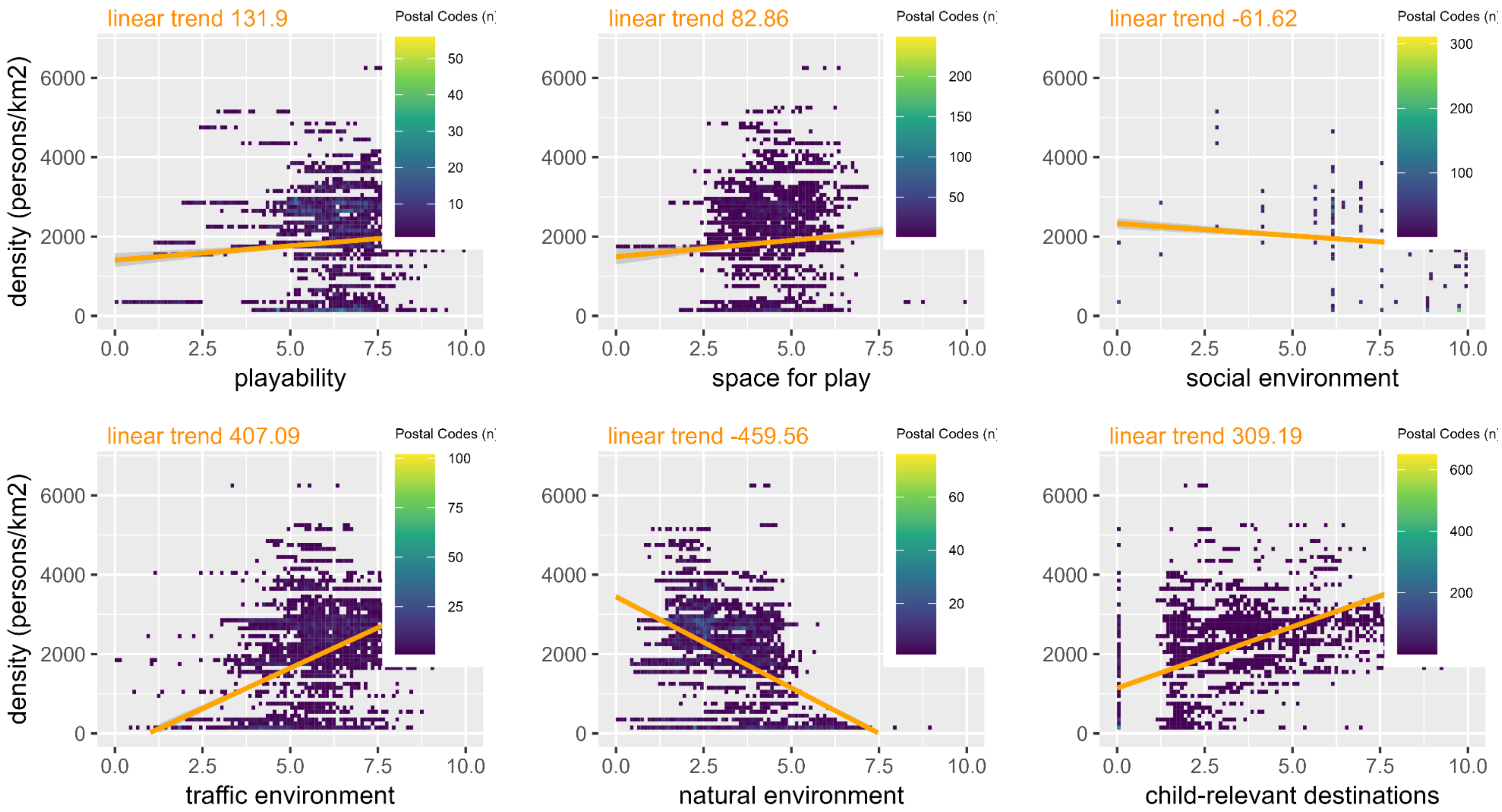

*replaced extreme outliers (values above the 0.05th and 99.95th percentile) with 0.05th and 99.95th percentile values to enable visualization

Figure D.17. Thunder Bay density plots and trendlines for relationship between postal code-level playability, domains and population density*

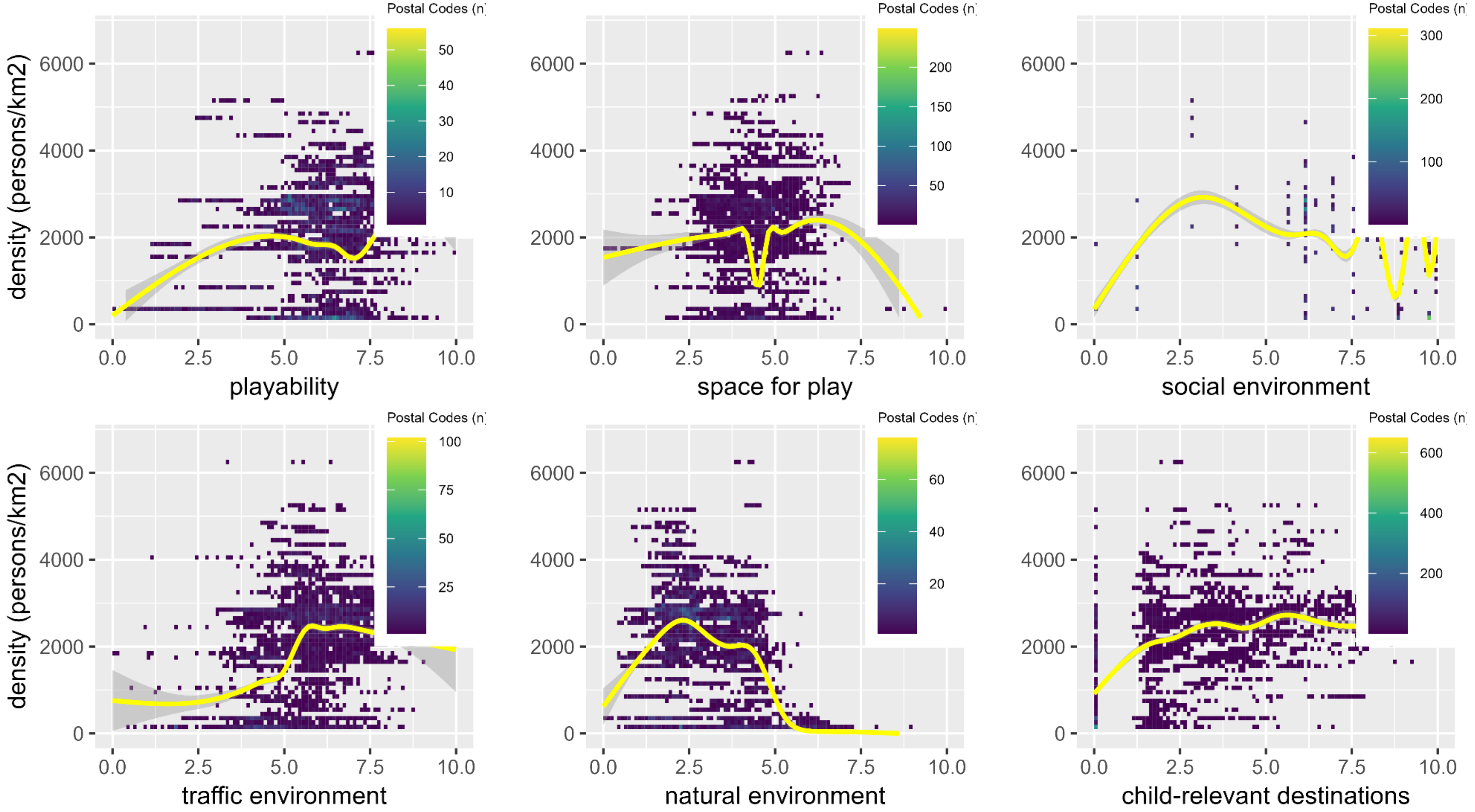

*Generalized additive models can be used to obtain a smoothed response function for the relationship between two variables. In this instance, a piecewise cubic function (spline) is used to approximate the relationship between playability, domains and population density.

**replaced extreme outliers (values above the 0.05th and 99.95th percentile) with 0.05th and 99.95th percentile values to enable visualization

Figure D.18. Thunder Bay smoothed trendlines (generalized additive models*) for relationship between postal code-level playability, domains and population density**

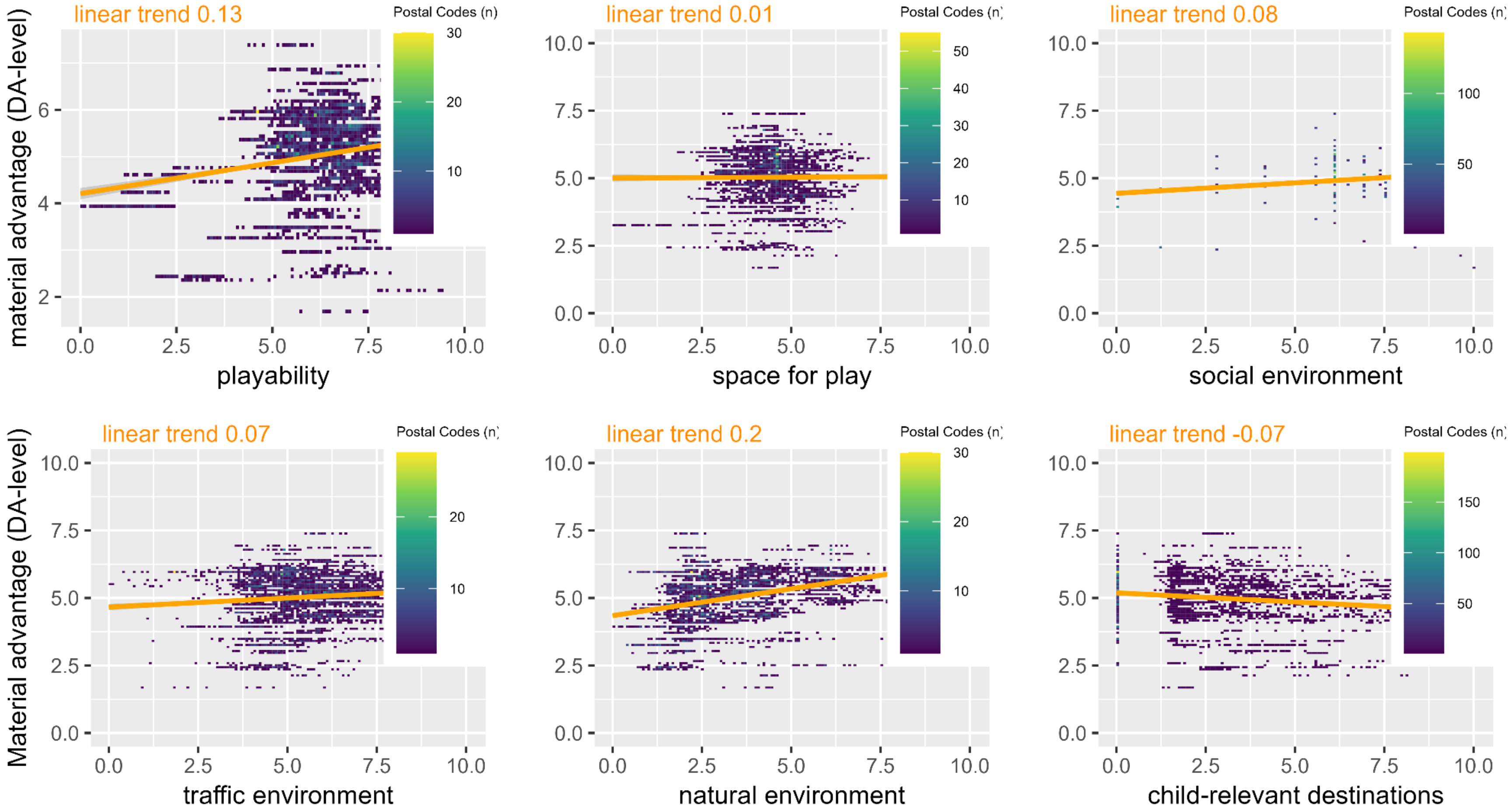

Figure D.19. Thunder Bay density plots and linear trendlines for relationship between postal code-level playability, domains and material advantage.

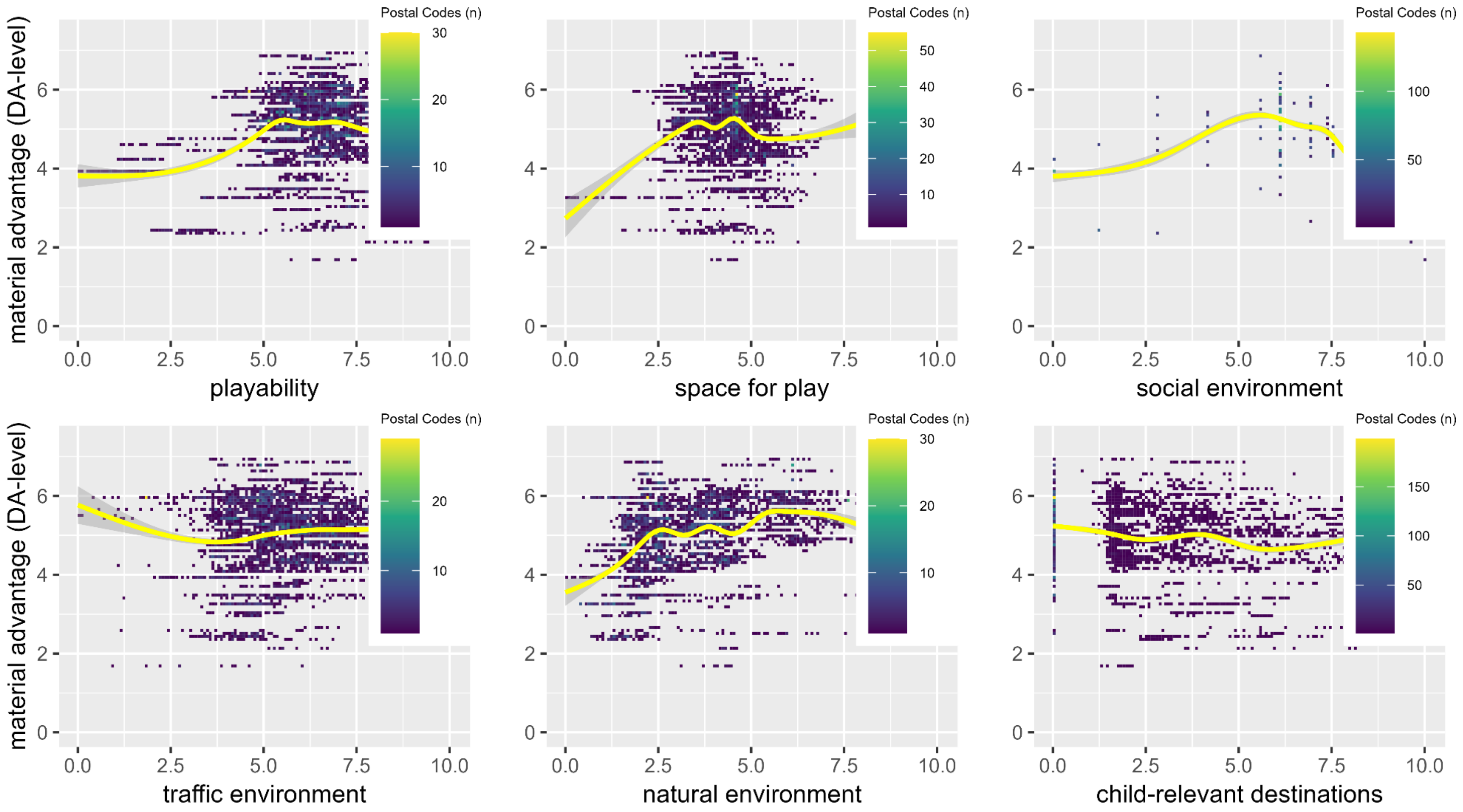

Generalized additive models can be used to obtain a smoothed response function for the relationship between two variables. In this instance, a piecewise cubic function (spline) is used to approximate the relationship between playability, domains and population density.

Figure D.20. Thunder Bay smoothed trendlines (generalized additive models*) for relationships between playability, domains and material advantage.

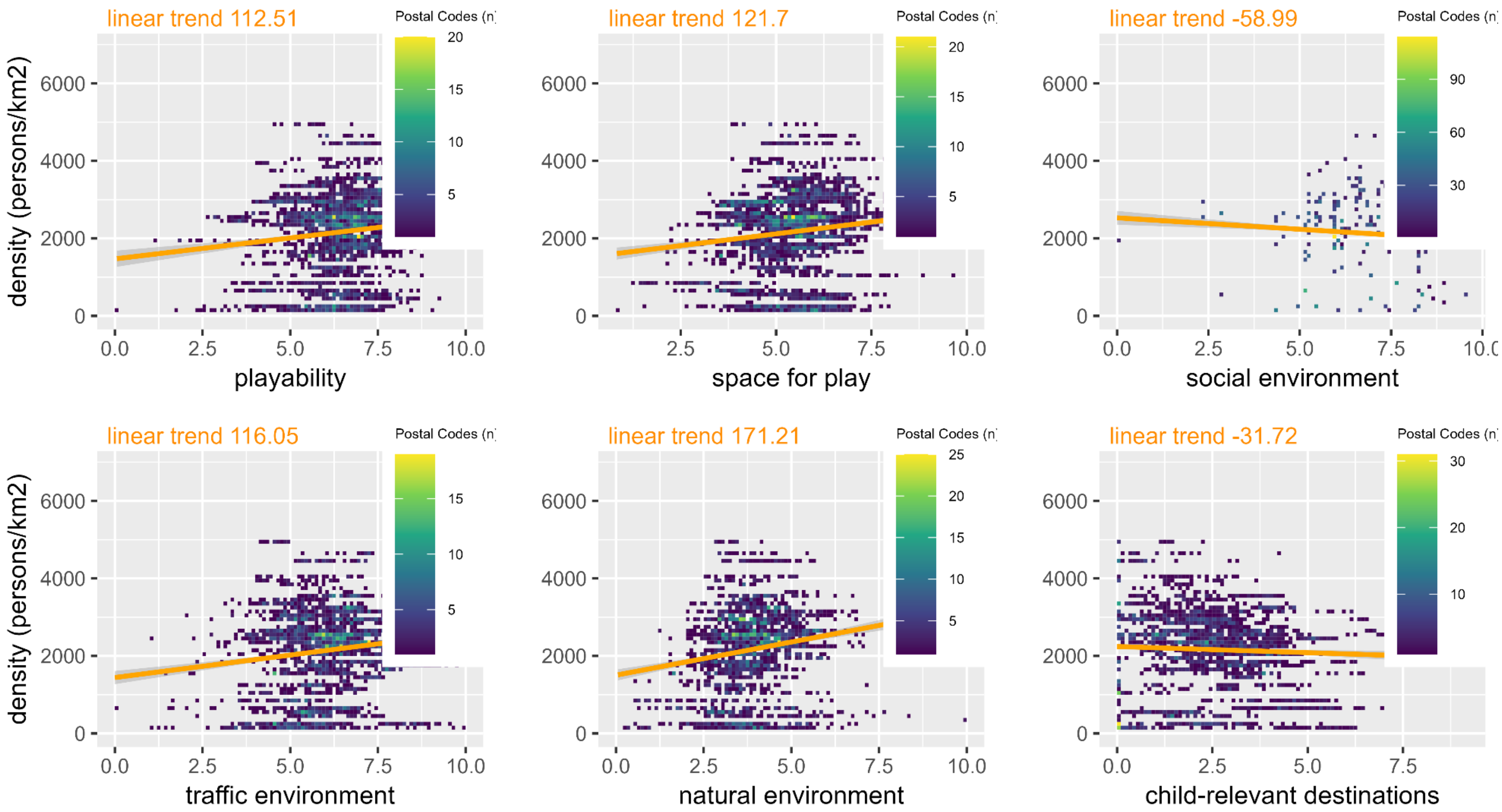

*replaced extreme outliers (values above the 0.05th and 99.95th percentile) with 0.05th and 99.95th percentile values to enable visualization

Figure D.21. Lethbridge density plots and trendlines for relationship between postal code-level playability, domains and population density*

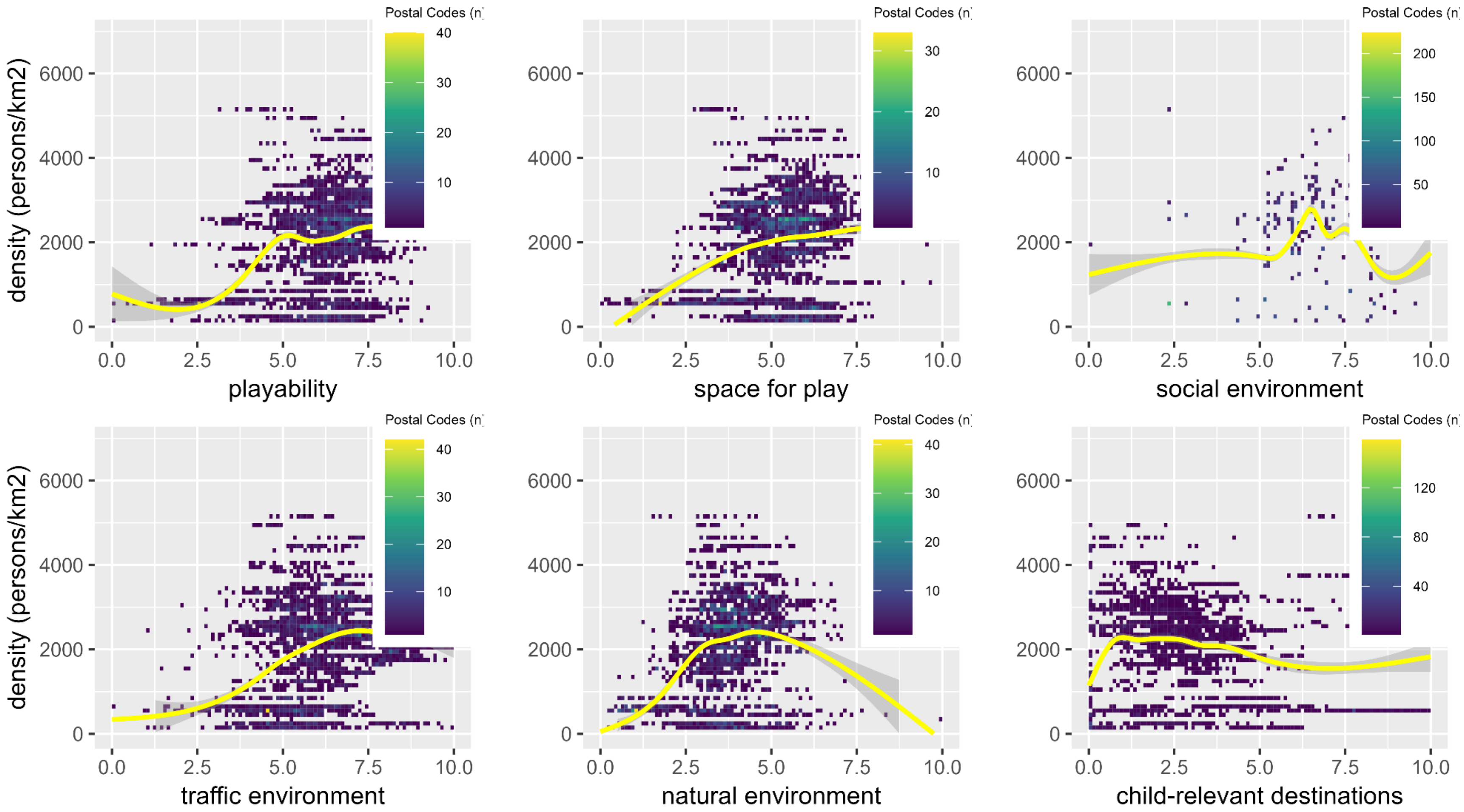

*Generalized additive models can be used to obtain a smoothed response function for the relationship between two variables. In this instance, a piecewise cubic function (spline) is used to approximate the relationship between playability, domains and population density.

**replaced extreme outliers (values above the 0.05th and 99.95th percentile) with 0.05th and 99.95th percentile values to enable visualization

Figure D.22. Lethbridge smoothed trendlines (generalized additive models*) for relationship between postal code-level playability, domains and population density**

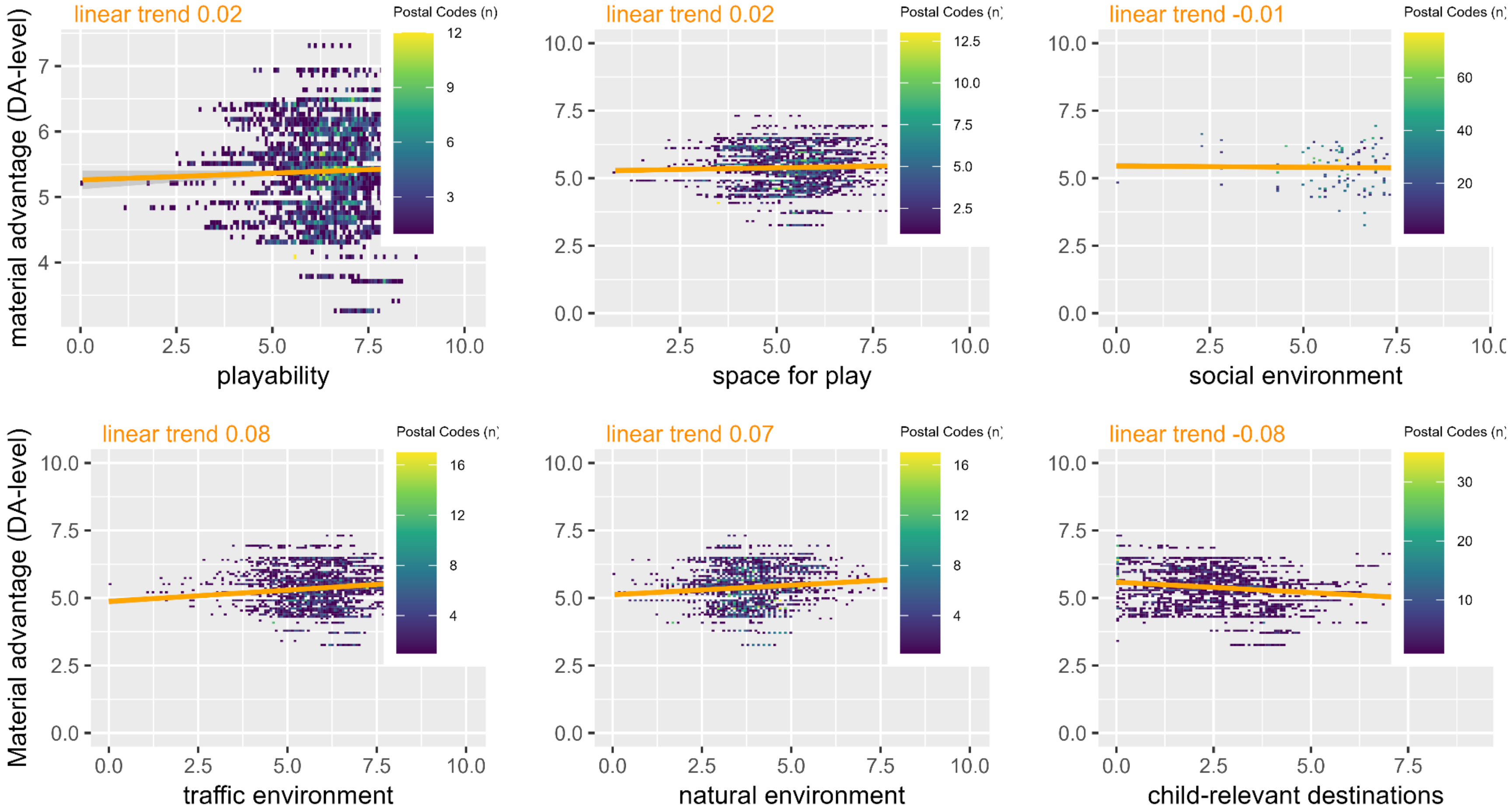

Figure D.23. Lethbridge density plots and linear trendlines for relationship between postal code-level playability, domains and material advantage

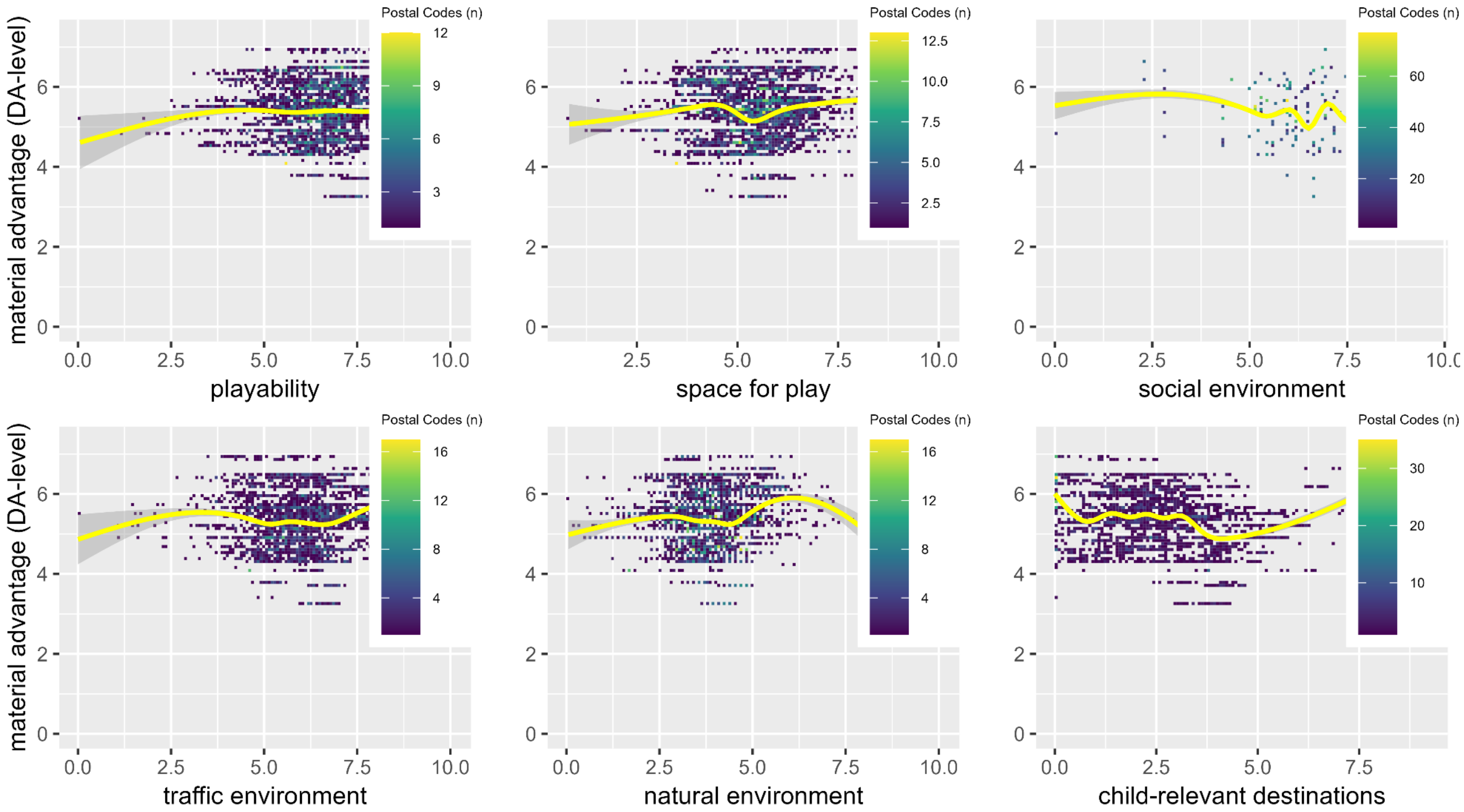

Generalized additive models can be used to obtain a smoothed response function for the relationship between two variables. In this instance, a piecewise cubic function (spline) is used to approximate the relationship between playability, domains and population density.

Figure D.24. Lethbridge smoothed trendlines (generalized additive models*) for relationships between playability, domains and material advantage.

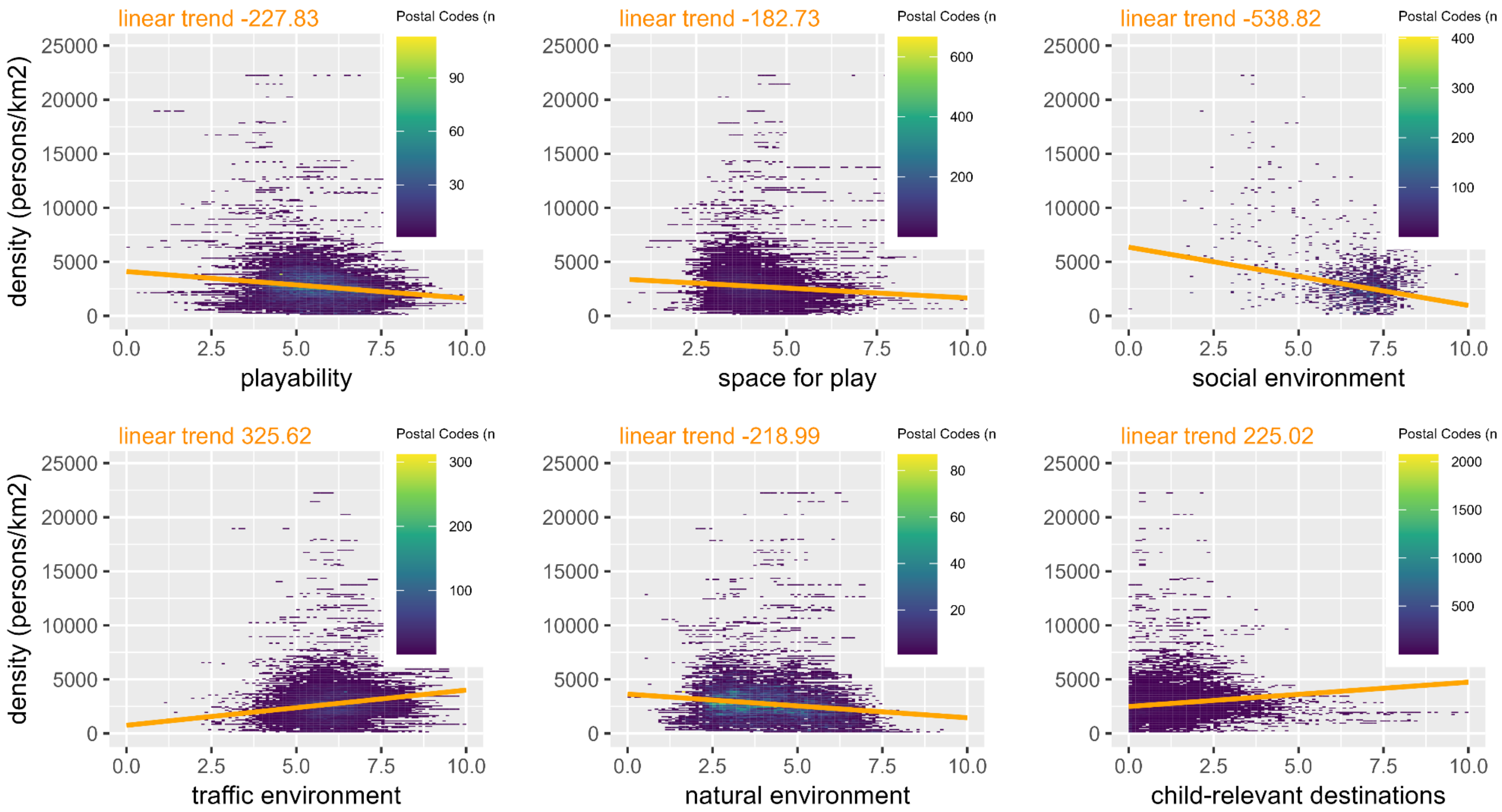

*replaced extreme outliers (values above the 0.05th and 99.95th percentile) with 0.05th and 99.95th percentile values to enable visualization

Figure D.25. Edmonton density plots and trendlines for relationship between postal code-level playability, domains and population density*

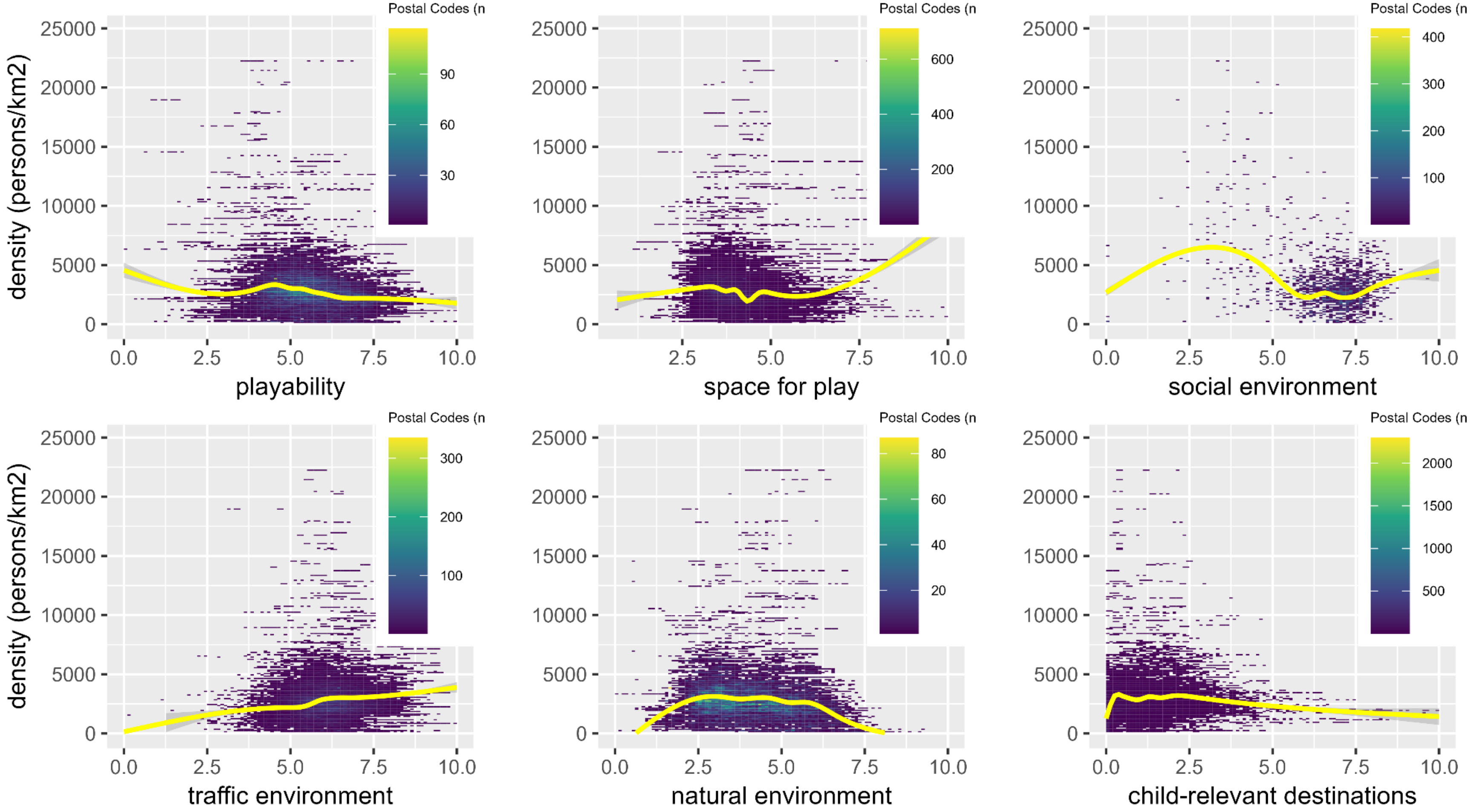

*Generalized additive models can be used to obtain a smoothed response function for the relationship between two variables. In this instance, a piecewise cubic function (spline) is used to approximate the relationship between playability, domains and population density.

**replaced extreme outliers (values above the 0.05th and 99.95th percentile) with 0.05th and 99.95th percentile values to enable visualization

Figure D.26. Edmonton smoothed trendlines (generalized additive models*) for relationship between postal code-level playability, domains and population density**

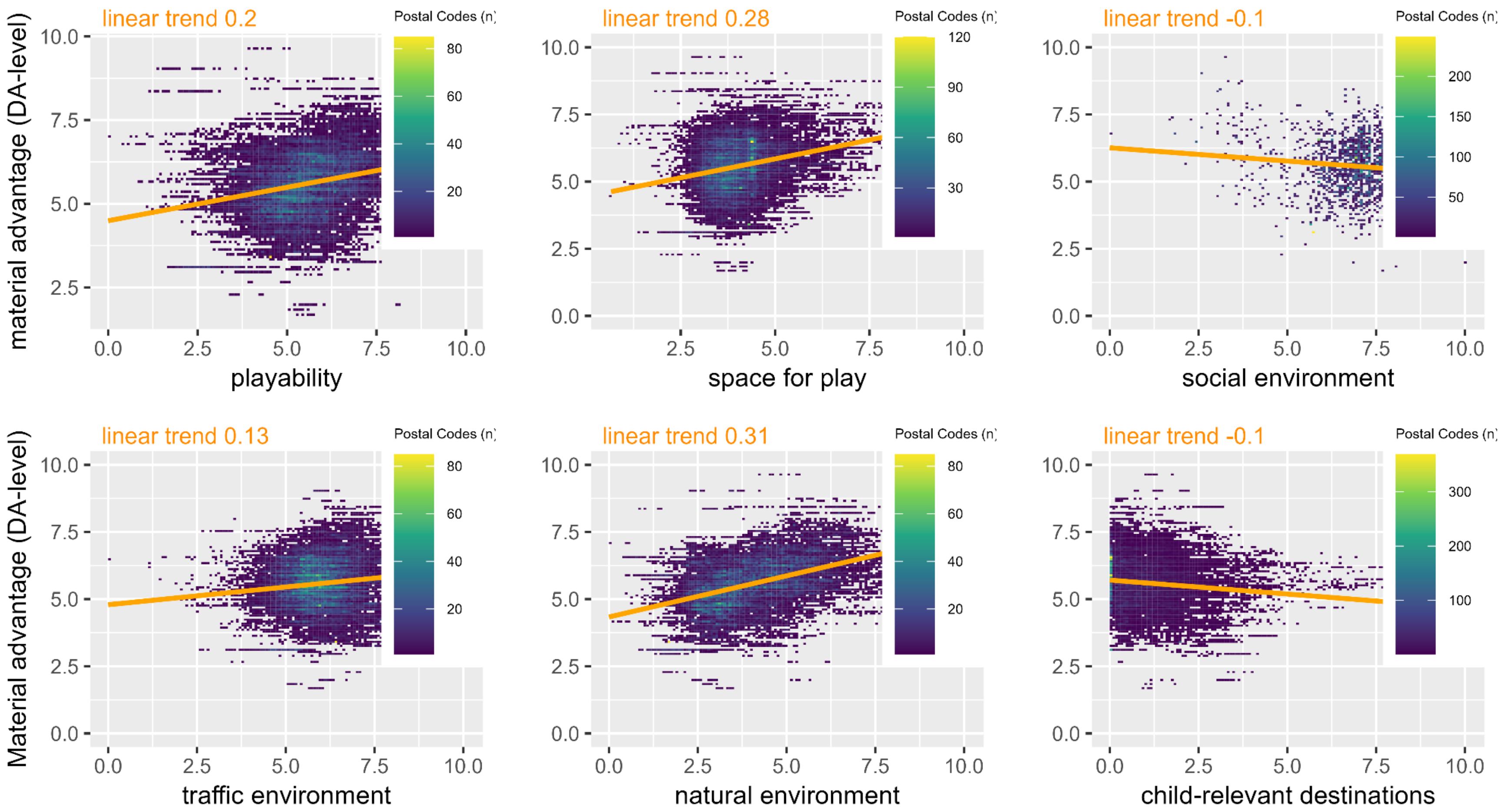

Figure D.27. Edmonton density plots and linear trendlines for relationship between postal code-level playability, domains and material advantage.

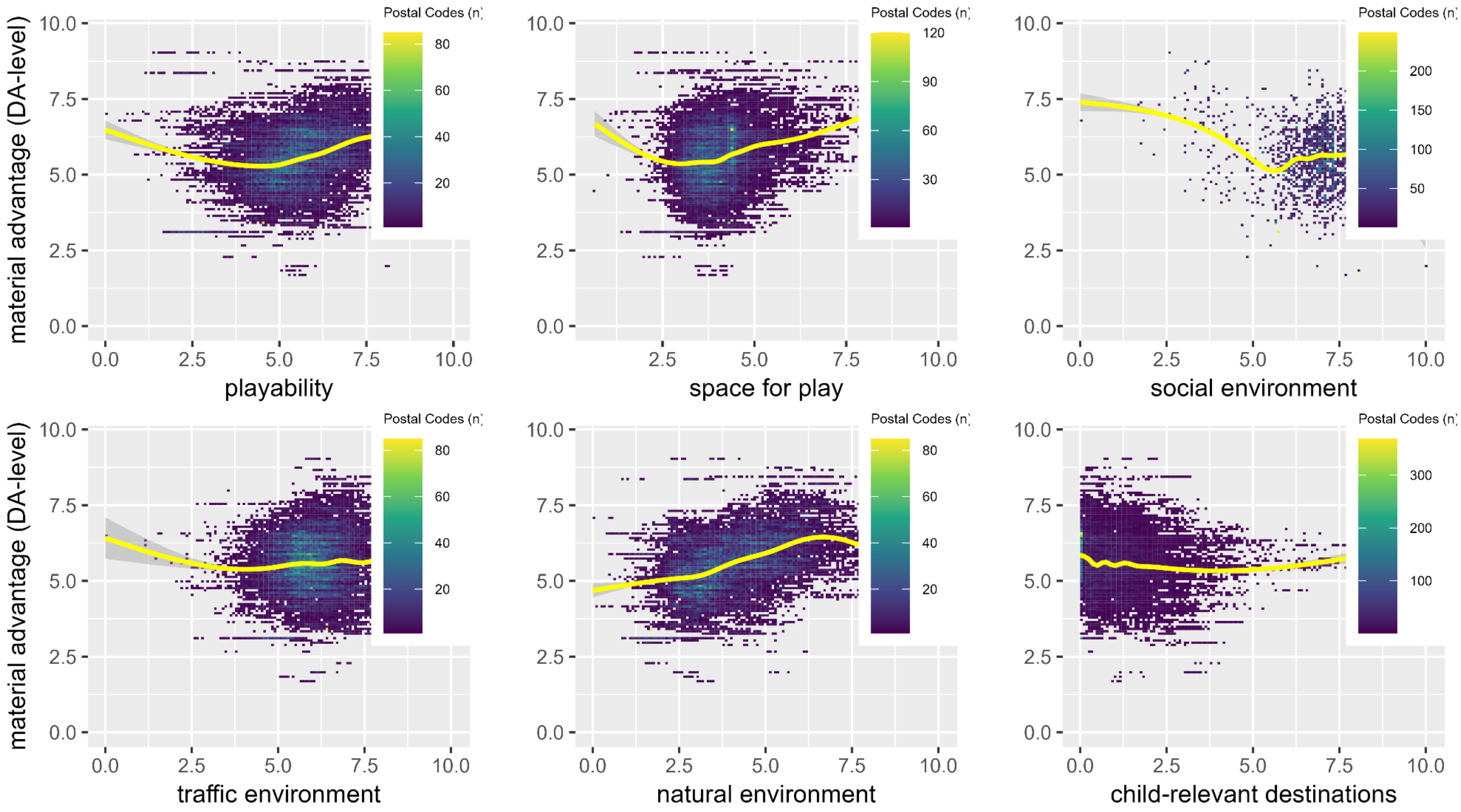

Generalized additive models can be used to obtain a smoothed response function for the relationship between two variables. In this instance, a piecewise cubic function (spline) is used to approximate the relationship between playability, domains and population density.

Figure D.28. Edmonton smoothed trendlines (generalized additive models*) for relationships between playability, domains and material advantage.

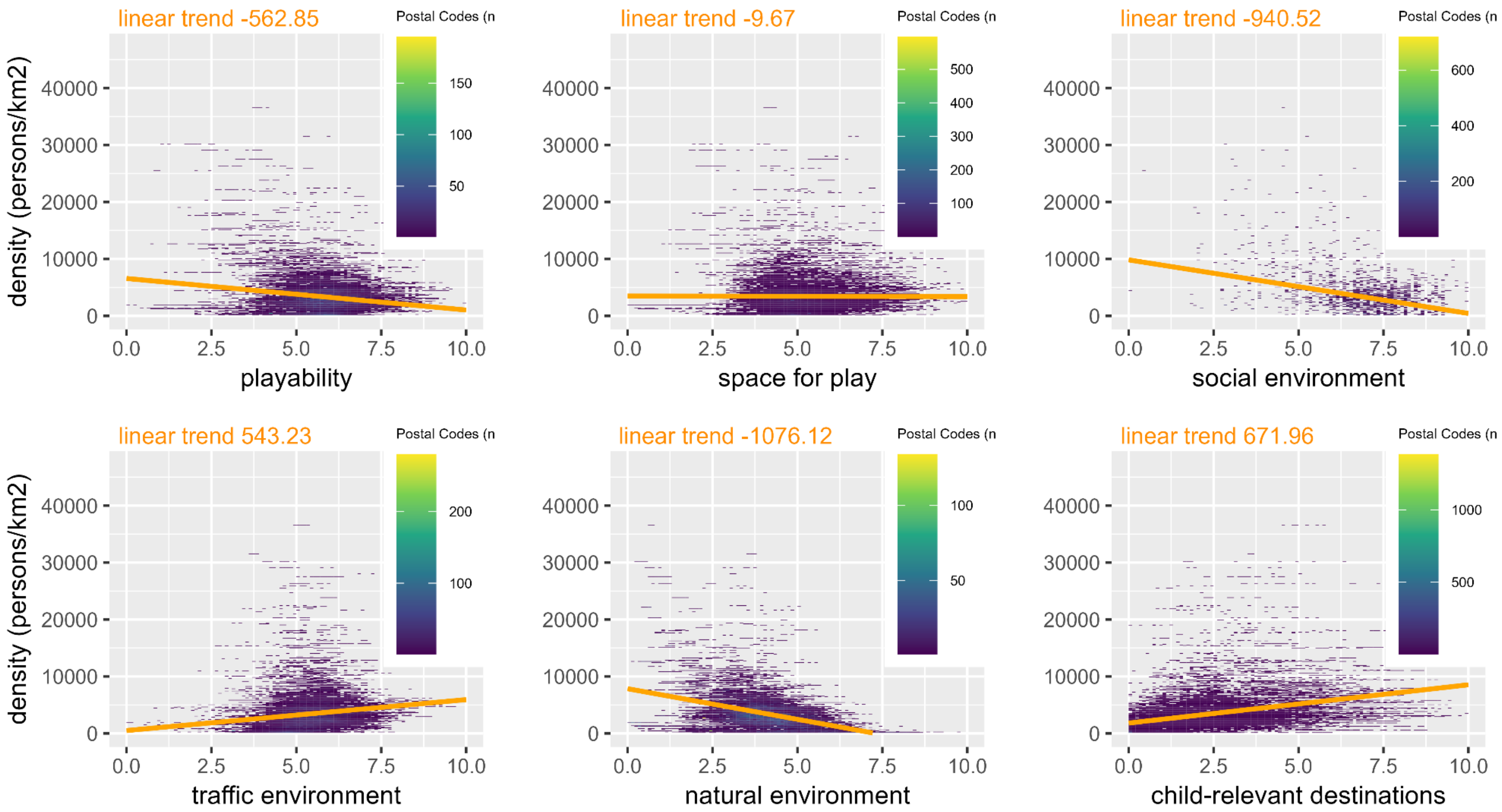

*replaced extreme outliers (values above the 0.05th and 99.95th percentile) with 0.05th and 99.95th percentile values to enable visualization

Figure D.29. Ottawa - Gatineau density plots and trendlines for relationship between postal code-level playability, domains and population density*

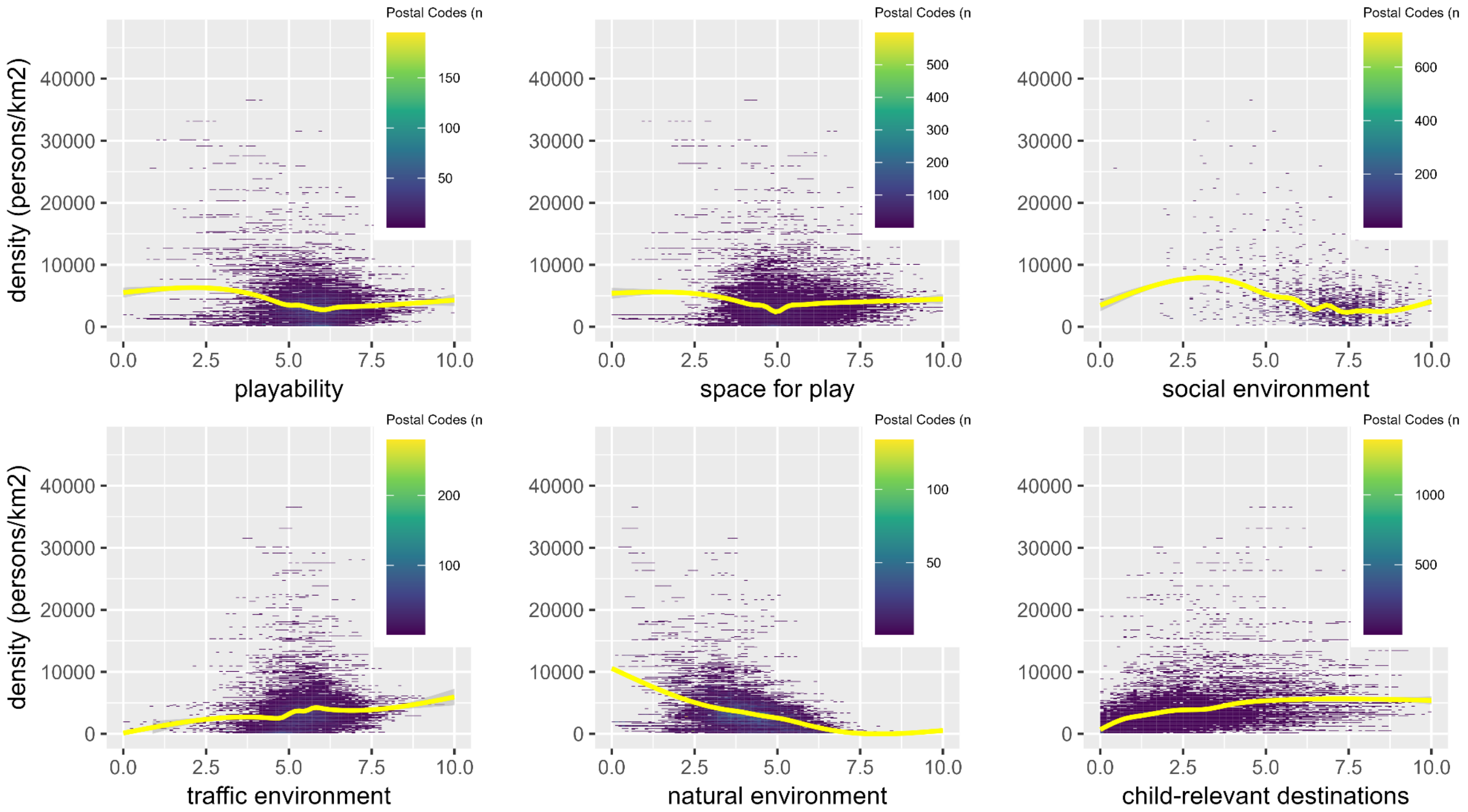

*Generalized additive models can be used to obtain a smoothed response function for the relationship between two variables. In this instance, a piecewise cubic function (spline) is used to approximate the relationship between playability, domains and population density.

**replaced extreme outliers (values above the 0.05th and 99.95th percentile) with 0.05th and 99.95th percentile values to enable visualization

Figure D.30. Ottawa - Gatineau smoothed trendlines (generalized additive models*) for relationship between postal code-level playability, domains and population density**

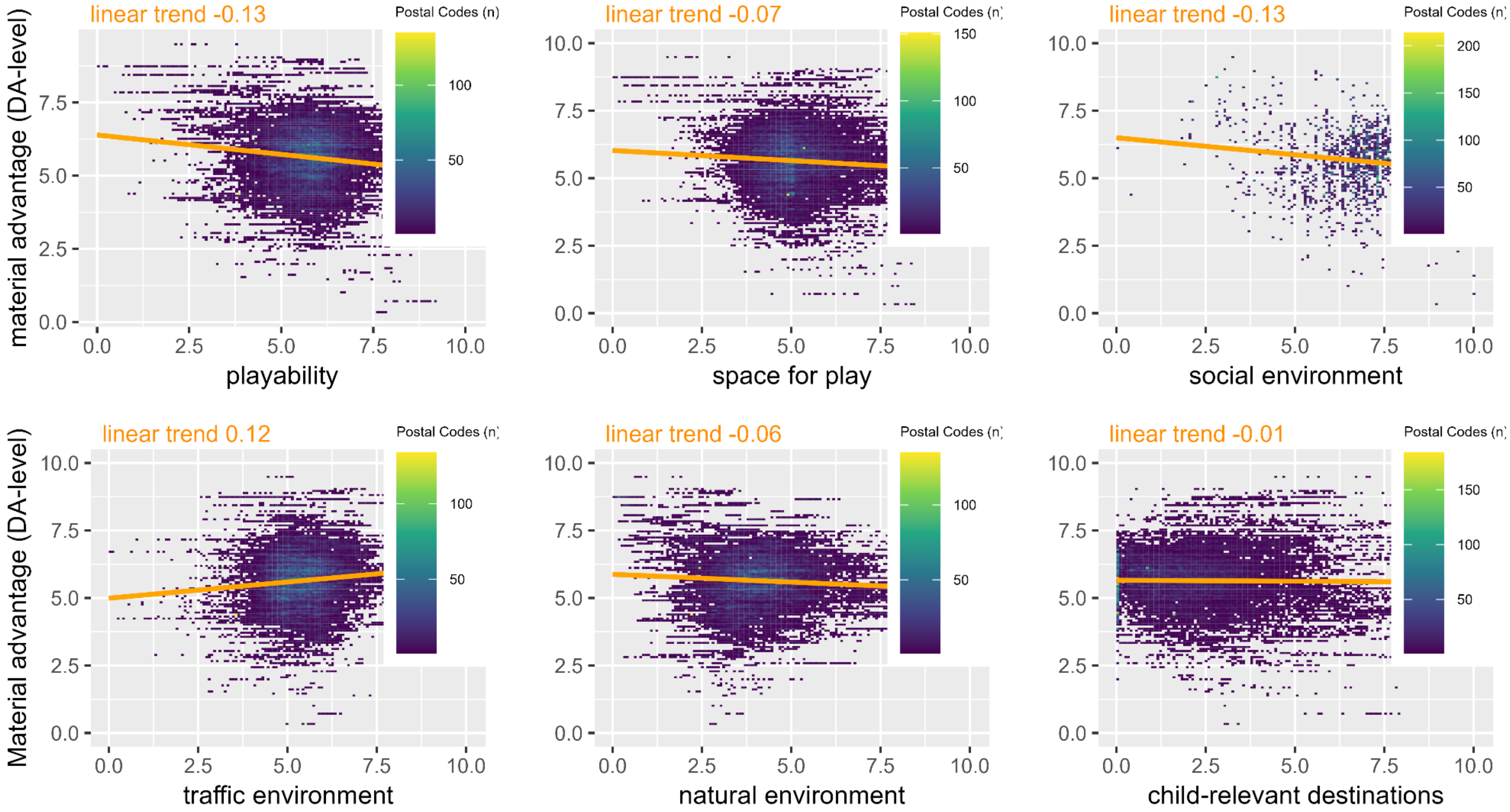

Figure D.31. Ottawa - Gatineau density plots and linear trendlines for relationship between postal code-level playability, domains and material advantage.

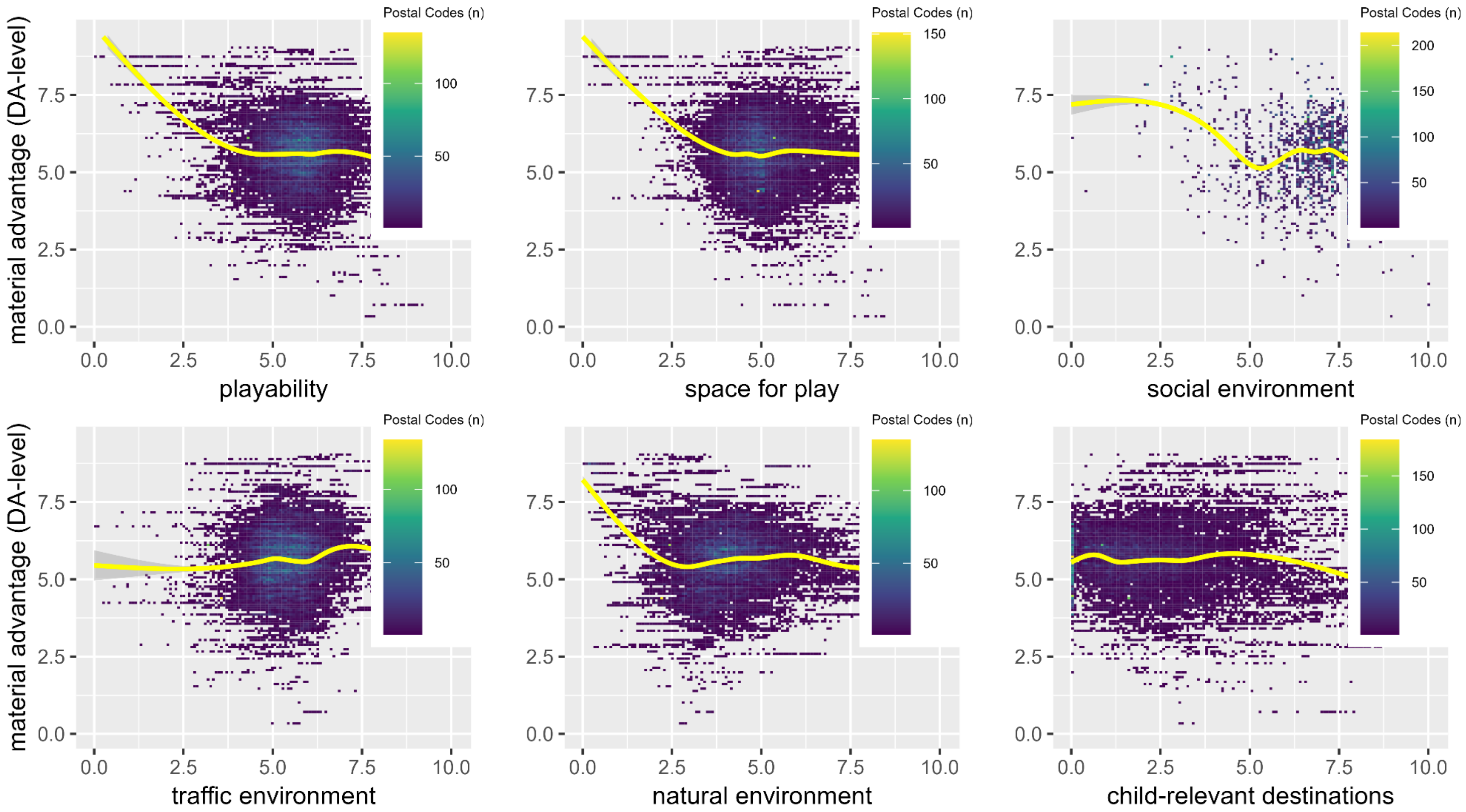

Generalized additive models can be used to obtain a smoothed response function for the relationship between two variables. In this instance, a piecewise cubic function (spline) is used to approximate the relationship between playability, domains and population density.

Figure D.32 Ottawa - Gatineau smoothed trendlines (generalized additive models*) for relationships between playability, domains and material advantage.

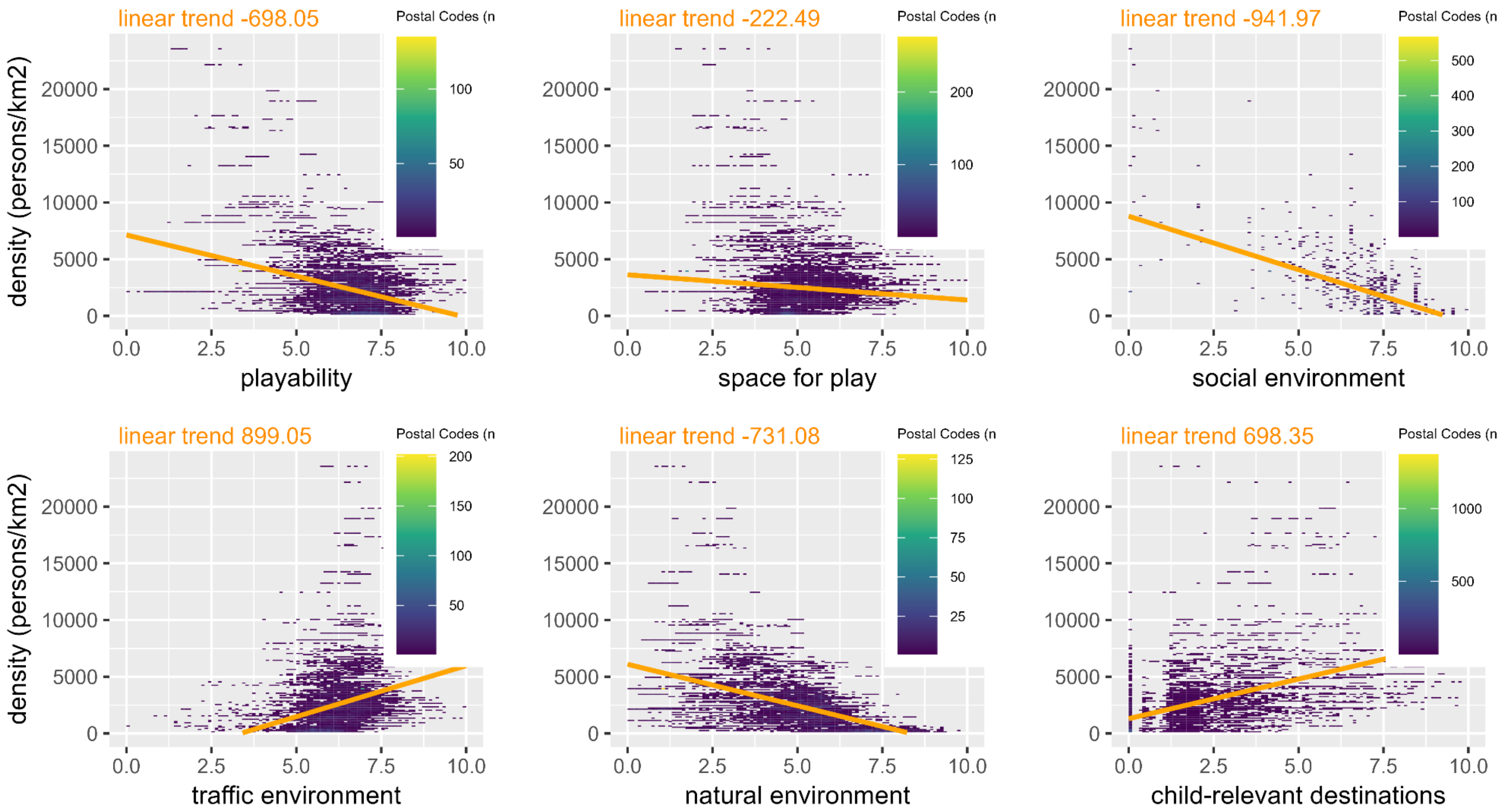

*replaced extreme outliers (values above the 0.05th and 99.95th percentile) with 0.05th and 99.95th percentile values to enable visualization

Figure D.33. Halifax density plots and trendlines for relationship between postal code-level playability, domains and population density*

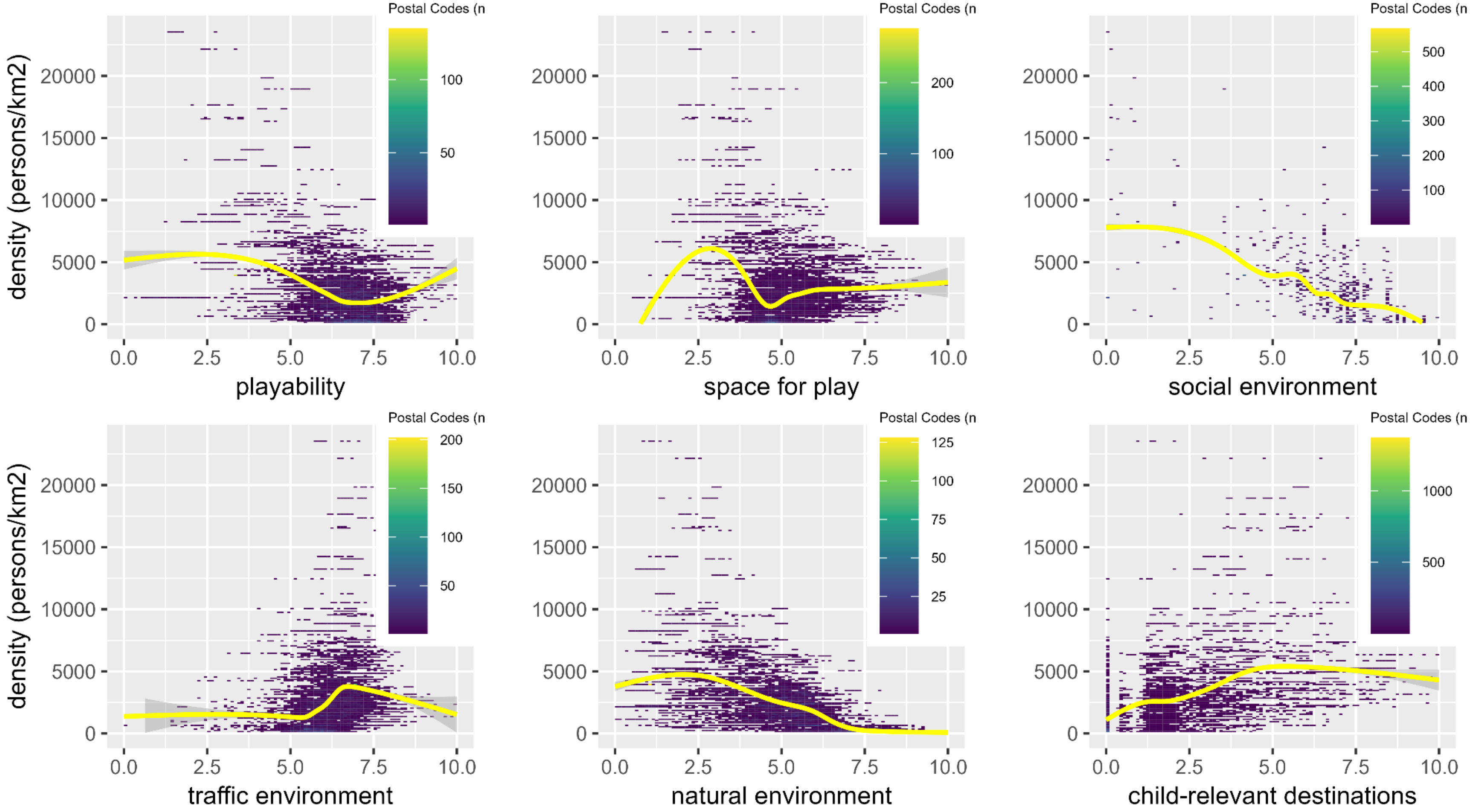

*Generalized additive models can be used to obtain a smoothed response function for the relationship between two variables. In this instance, a piecewise cubic function (spline) is used to approximate the relationship between playability, domains and population density.

**replaced extreme outliers (values above the 0.05th and 99.95th percentile) with 0.05th and 99.95th percentile values to enable visualization

Figure D.34. Halifax smoothed trendlines (generalized additive models*) for relationship between postal code-level playability, domains and population density**

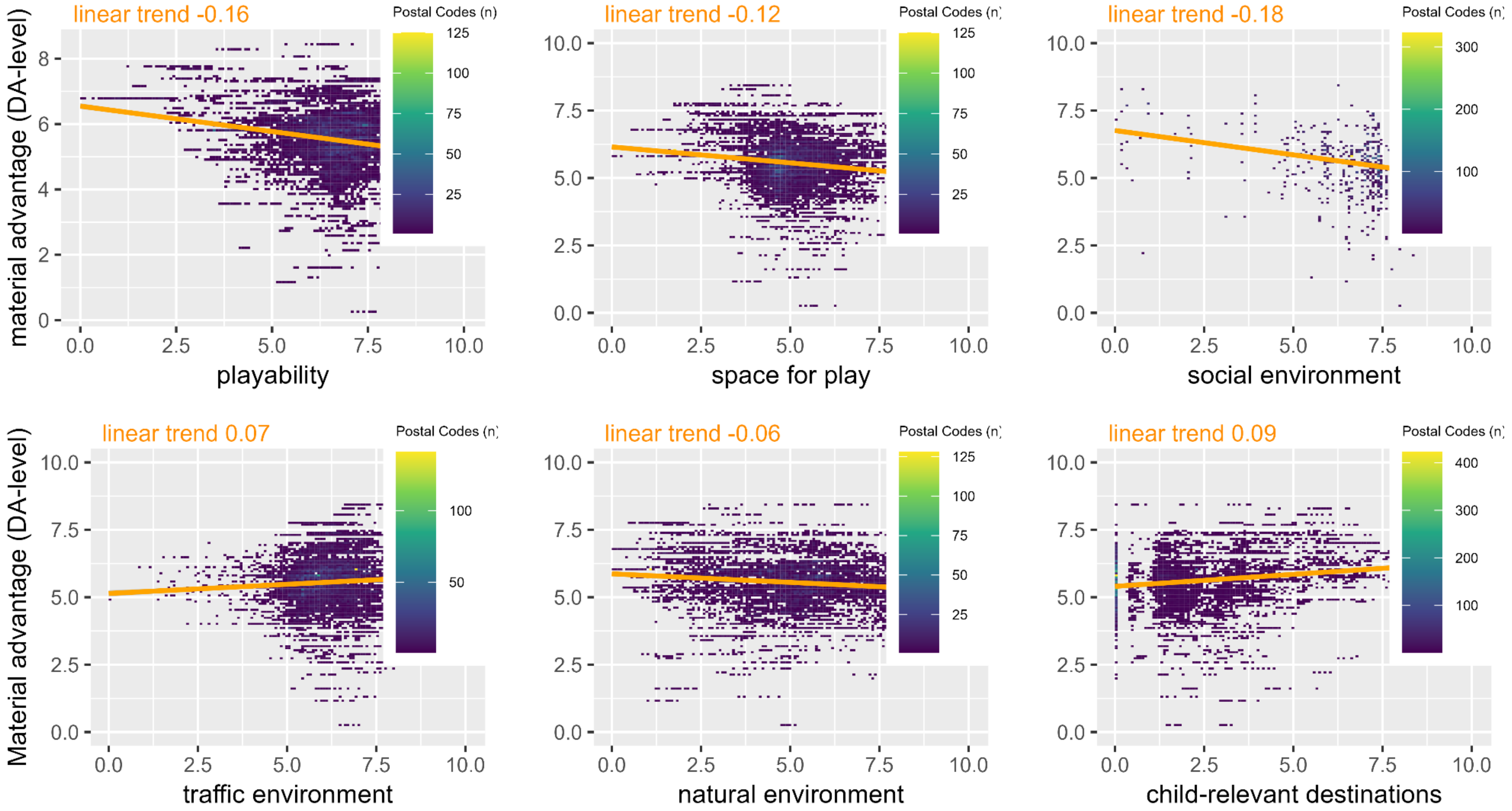

Figure D.35. Halifax density plots and linear trendlines for relationship between postal code-level playability, domains and material advantage.

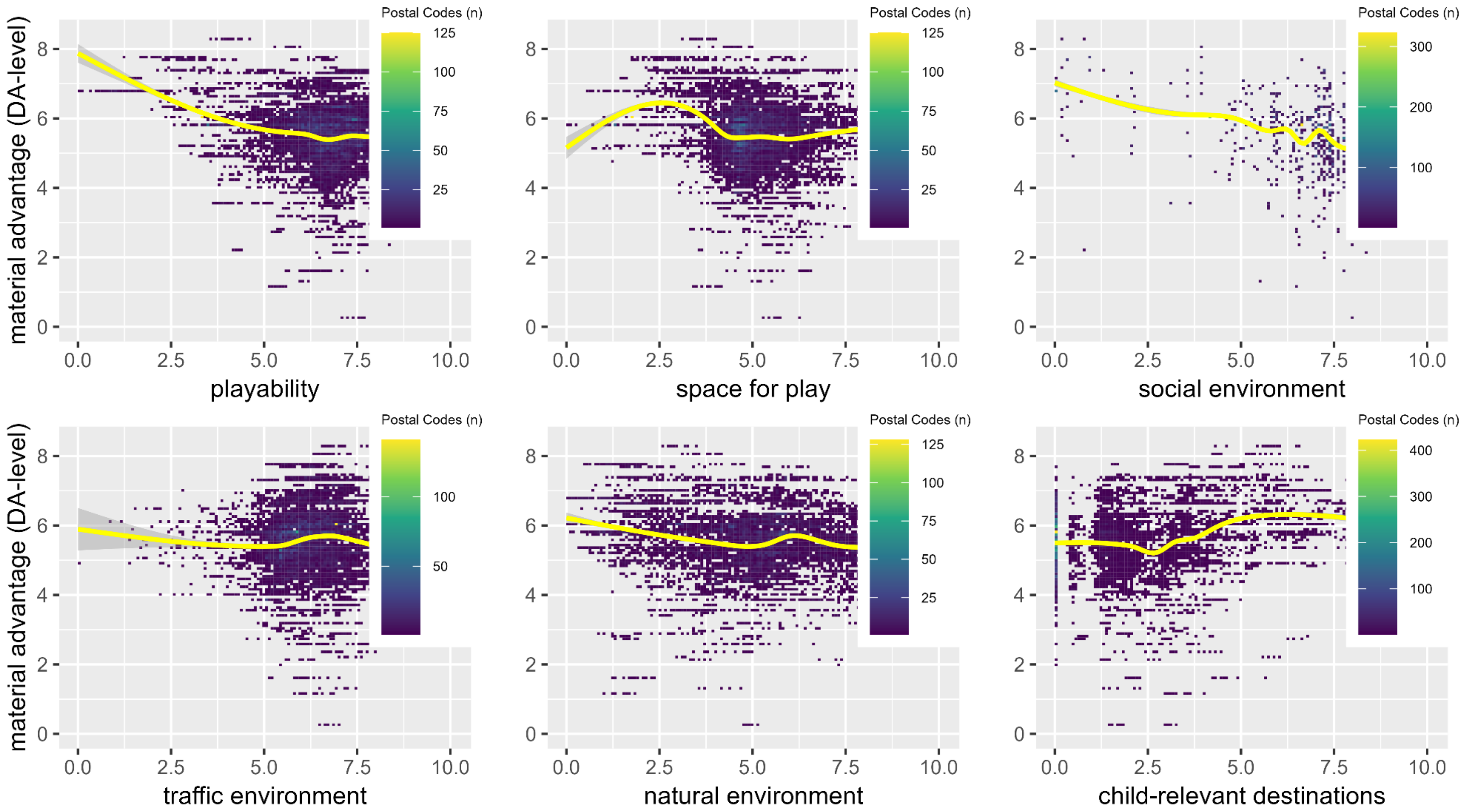

Generalized additive models can be used to obtain a smoothed response function for the relationship between two variables. In this instance, a piecewise cubic function (spline) is used to approximate the relationship between playability, domains and population density.

Figure D.36 Halifax smoothed trendlines (generalized additive models*) for relationships between playability, domains and material advantage.

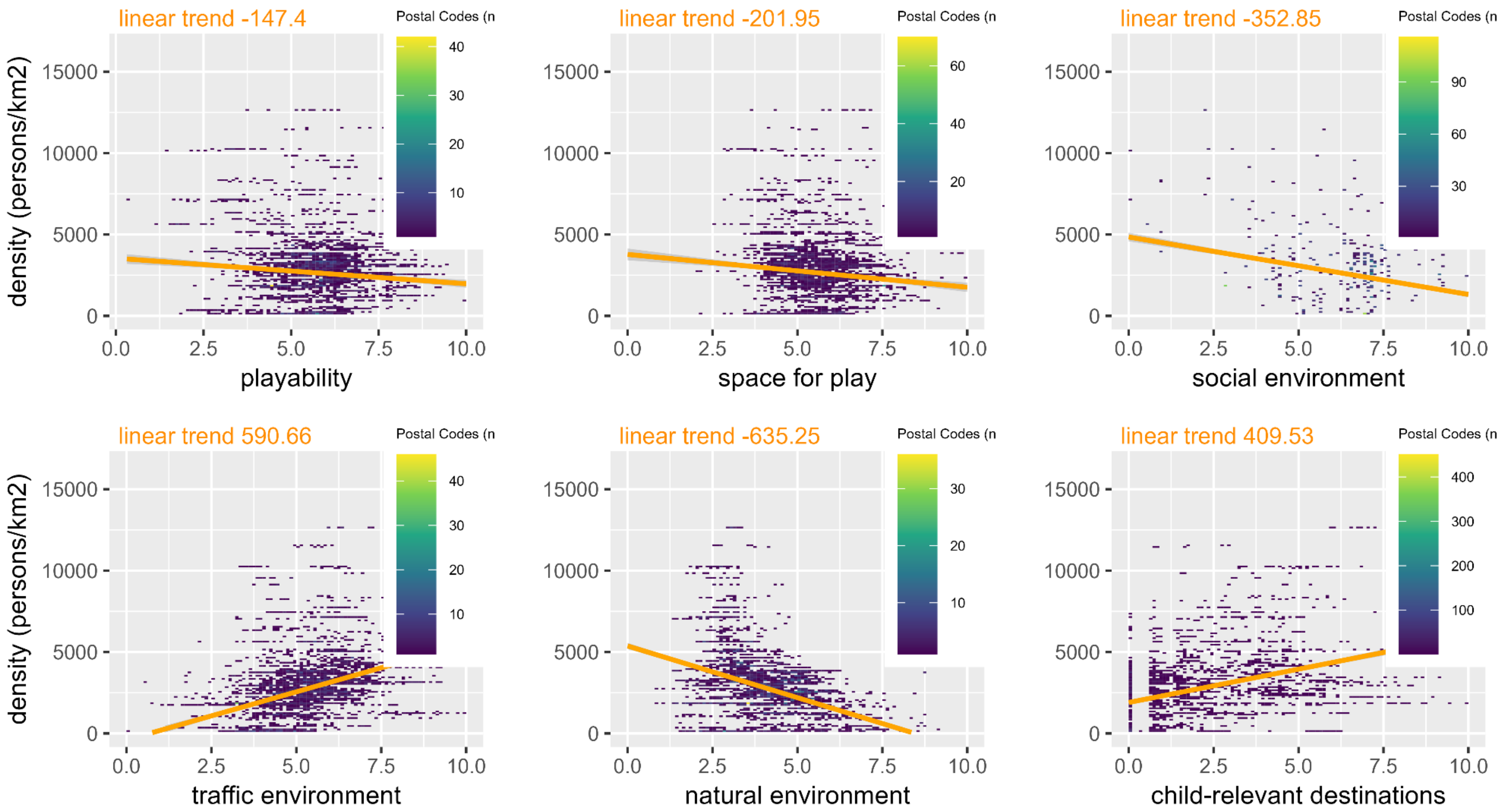

*replaced extreme outliers (values above the 0.05th and 99.95th percentile) with 0.05th and 99.95th percentile values to enable visualization

Figure D.37. Abbotsford density plots and trendlines for relationship between postal code-level playability, domains and population density*

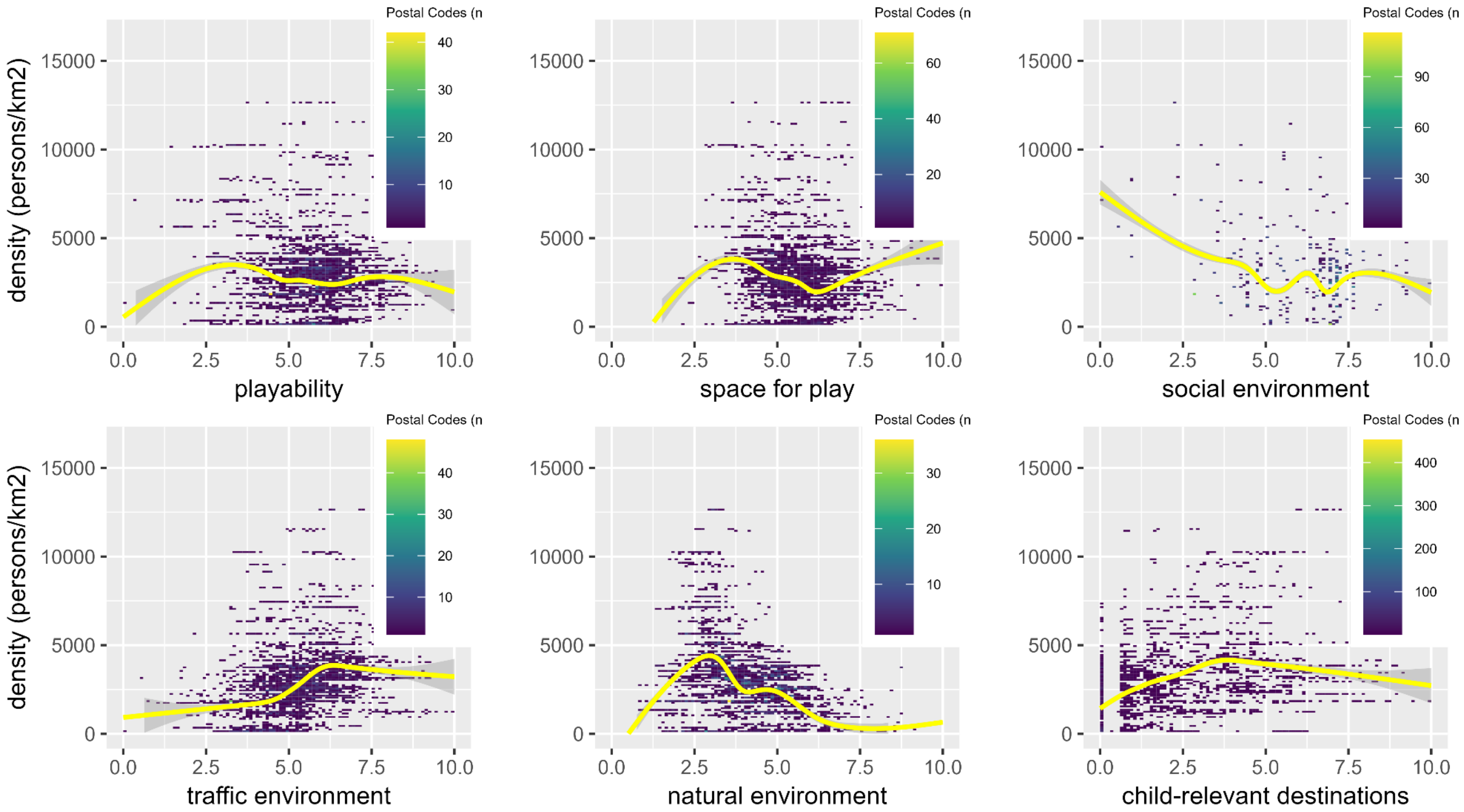

*Generalized additive models can be used to obtain a smoothed response function for the relationship between two variables. In this instance, a piecewise cubic function (spline) is used to approximate the relationship between playability, domains and population density.

**replaced extreme outliers (values above the 0.05th and 99.95th percentile) with 0.05th and 99.95th percentile values to enable visualization

Figure D.38. Abbotsford smoothed trendlines (generalized additive models*) for relationship between postal code-level playability, domains and population density**

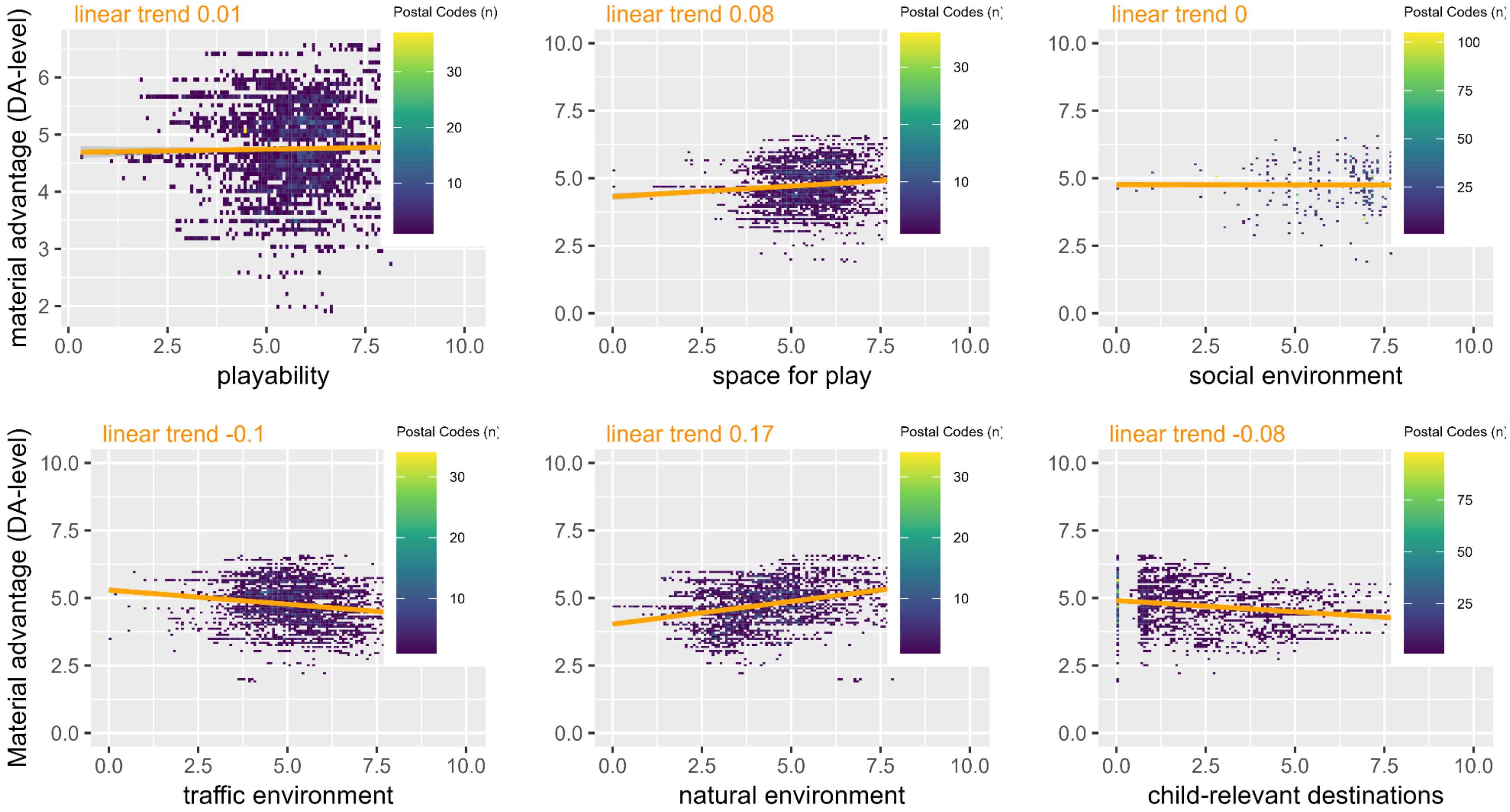

Figure D.39. Abbotsford density plots and trendlines for relationship between postal code-level playability, domains and material advantage.

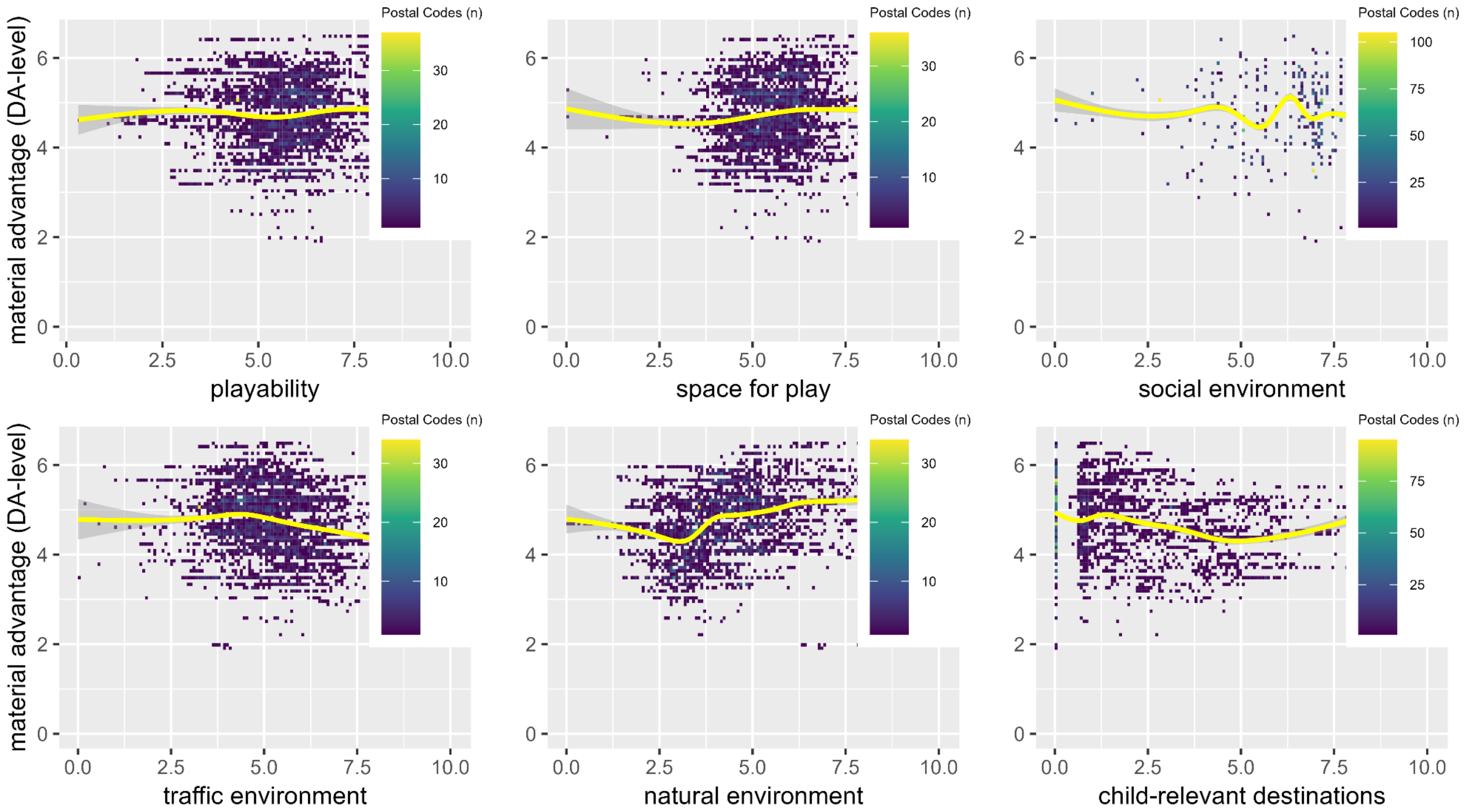

Generalized additive models can be used to obtain a smoothed response function for the relationship between two variables. In this instance, a piecewise cubic function (spline) is used to approximate the relationship between playability, domains and population density.

Figure D.40. Abbotsford smoothed trendlines (generalized additive models*) for relationships between playability, domains and material advantage.

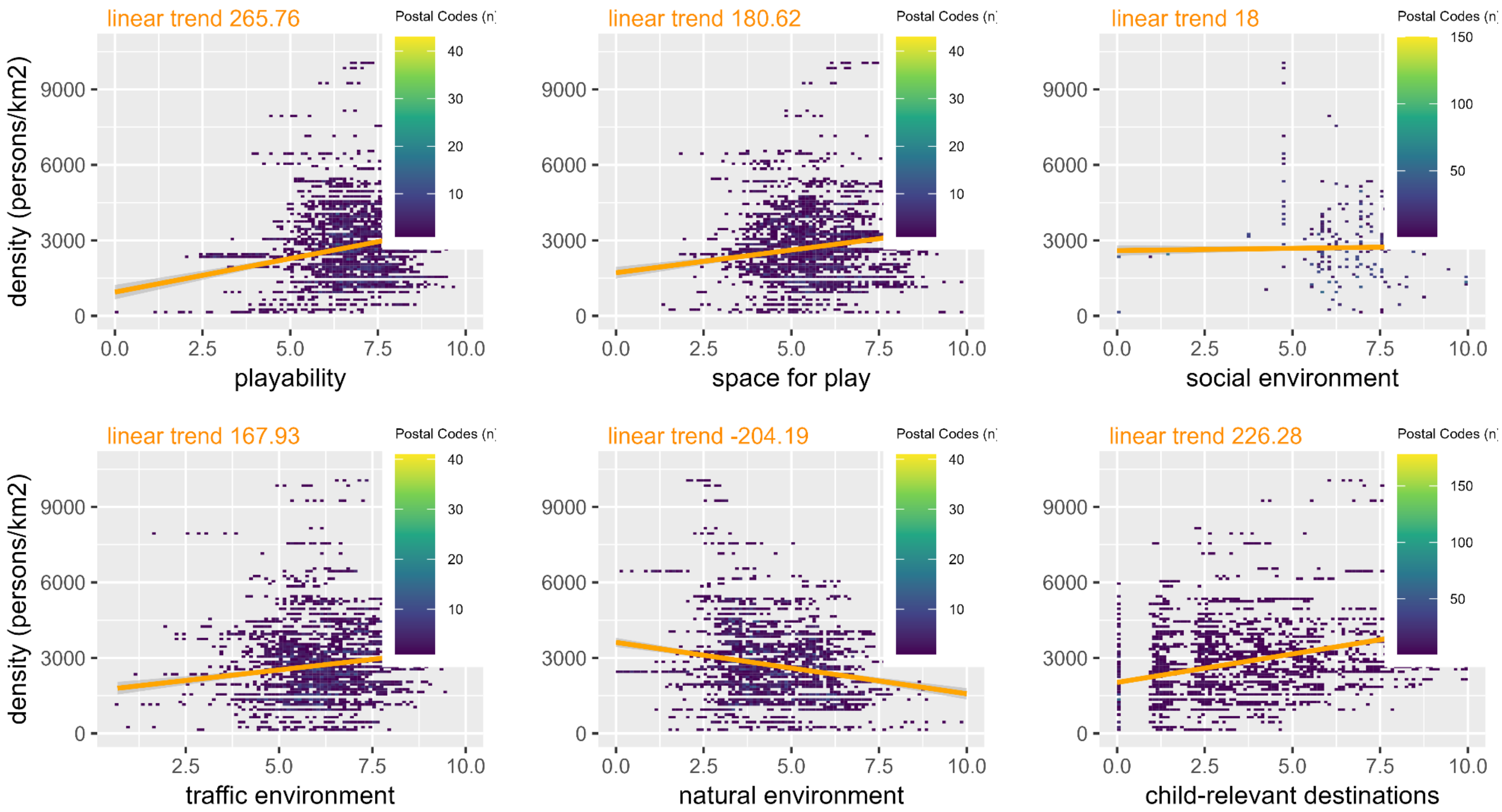

*replaced extreme outliers (values above the 0.05th and 99.95th percentile) with 0.05th and 99.95th percentile values to enable visualization

Figure D.41. Guelph density plots and trendlines for relationship between postal code-level playability, domains and population density*

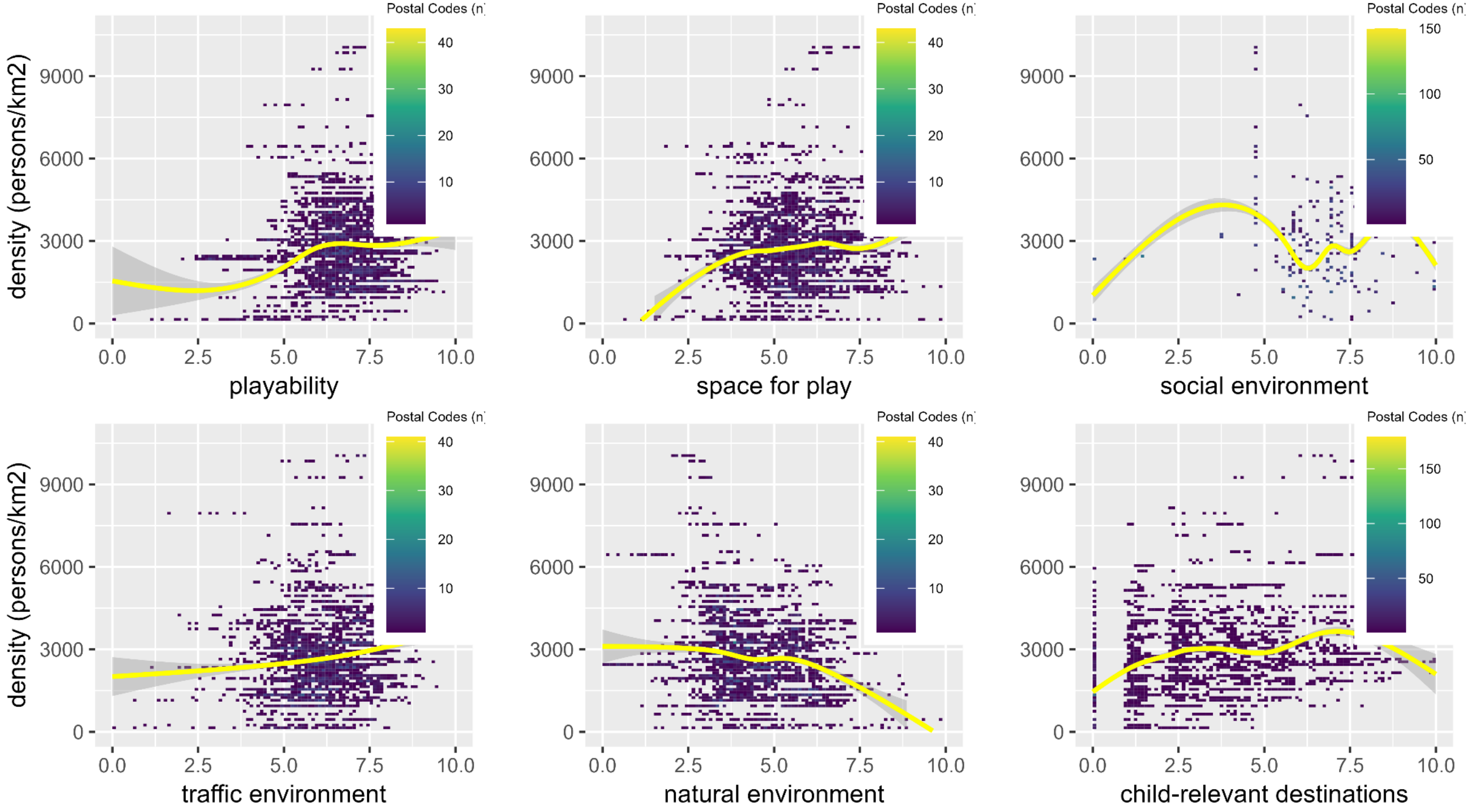

*Generalized additive models can be used to obtain a smoothed response function for the relationship between two variables. In this instance, a piecewise cubic function (spline) is used to approximate the relationship between playability, domains and population density.

**replaced extreme outliers (values above the 0.05th and 99.95th percentile) with 0.05th and 99.95th percentile values to enable visualization

Figure D.42. Guelph smoothed trendlines (generalized additive models*) for relationship between postal code-level playability, domains and population density**

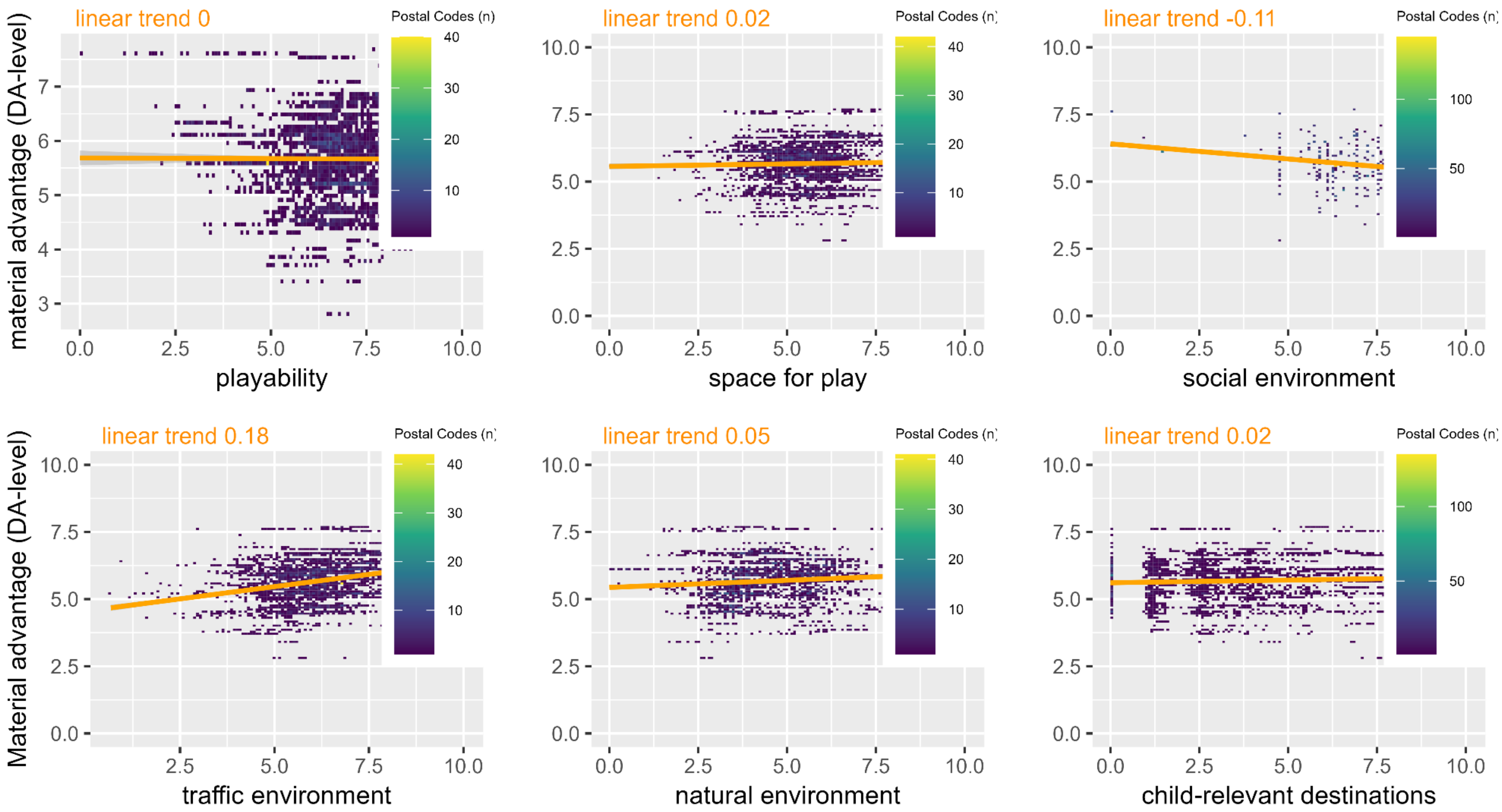

Figure D.43. Guelph density plots and linear trendlines for relationship between postal code-level playability, domains and material advantage

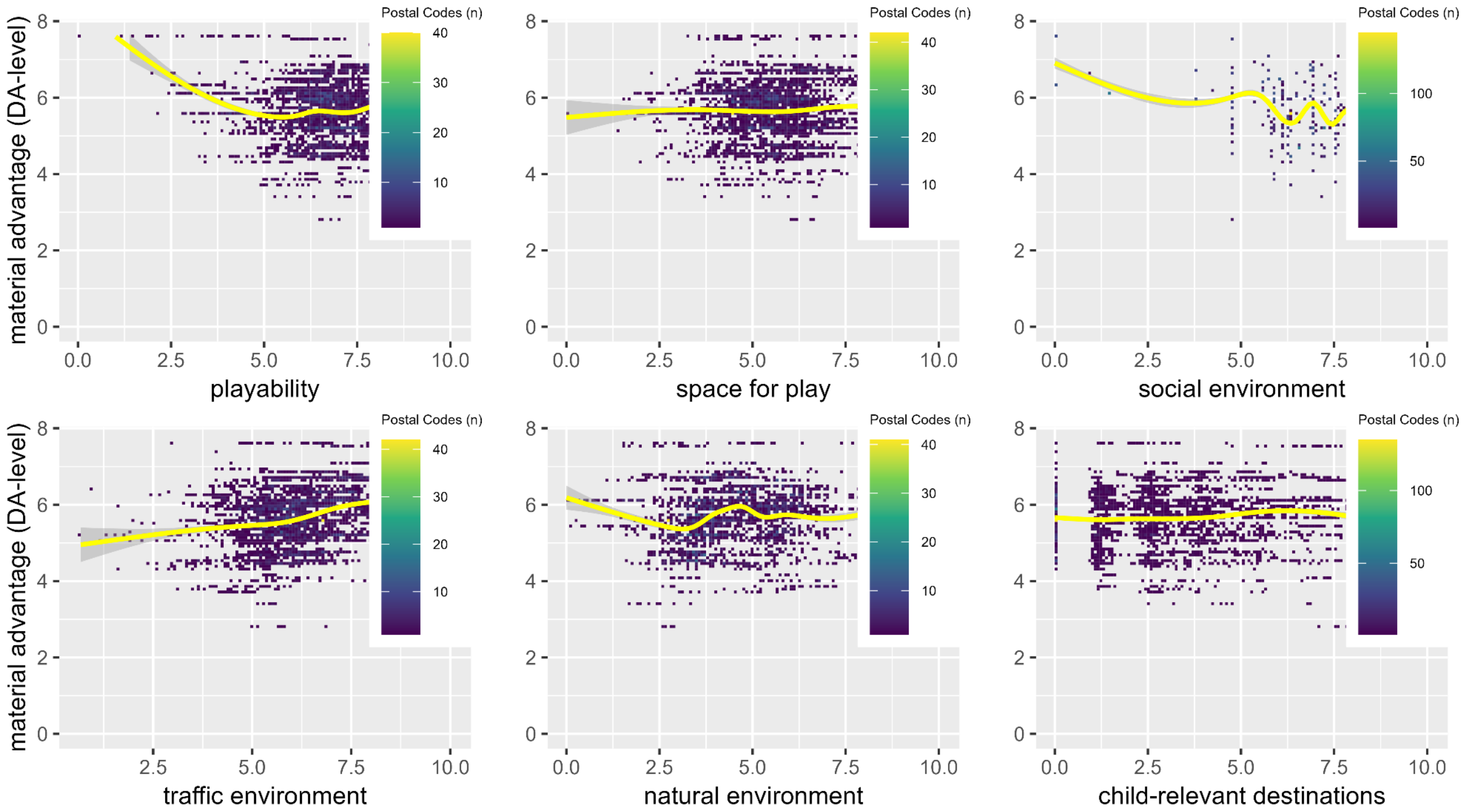

*Generalized additive models can be used to obtain a smoothed response function for the relationship between two variables. In this instance, a piecewise cubic function (spline) is used to approximate the relationship between playability, domains and population density.

Figure D.44. Guelph smoothed trendlines (generalized additive models*) for relationship between postal code-level playability, domains and material advantage.

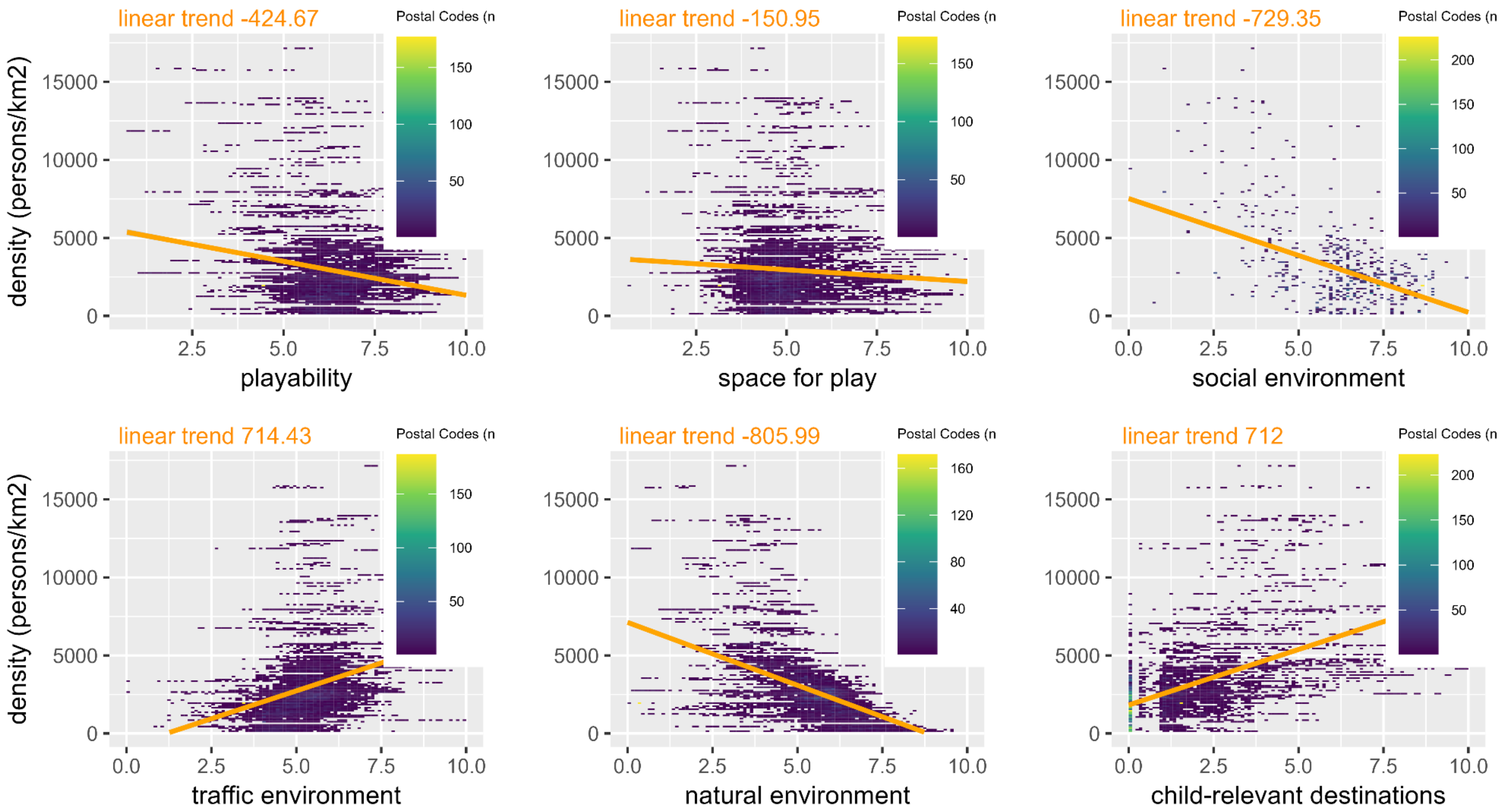

*replaced extreme outliers (values above the 0.05th and 99.95th percentile) with 0.05th and 99.95th percentile values to enable visualization

Figure D.45. Victoria density plots and trendlines for relationship between postal code-level playability, domains and population density*

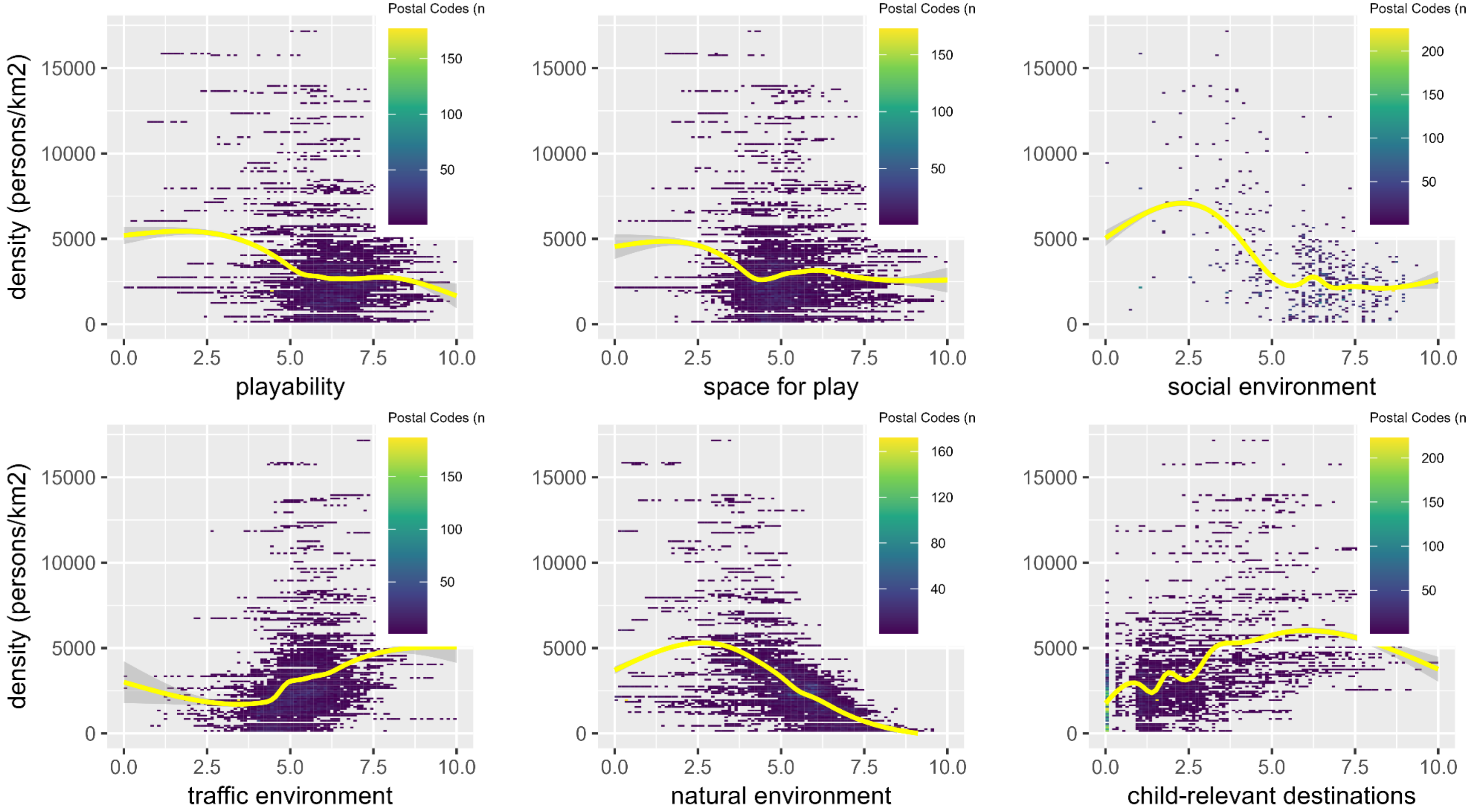

*Generalized additive models can be used to obtain a smoothed response function for the relationship between two variables. In this instance, a piecewise cubic function (spline) is used to approximate the relationship between playability, domains and population density.

Figure D.46. Victoria smoothed trendlines (generalized additive models*) for relationship between postal code-level playability, domains and population density**

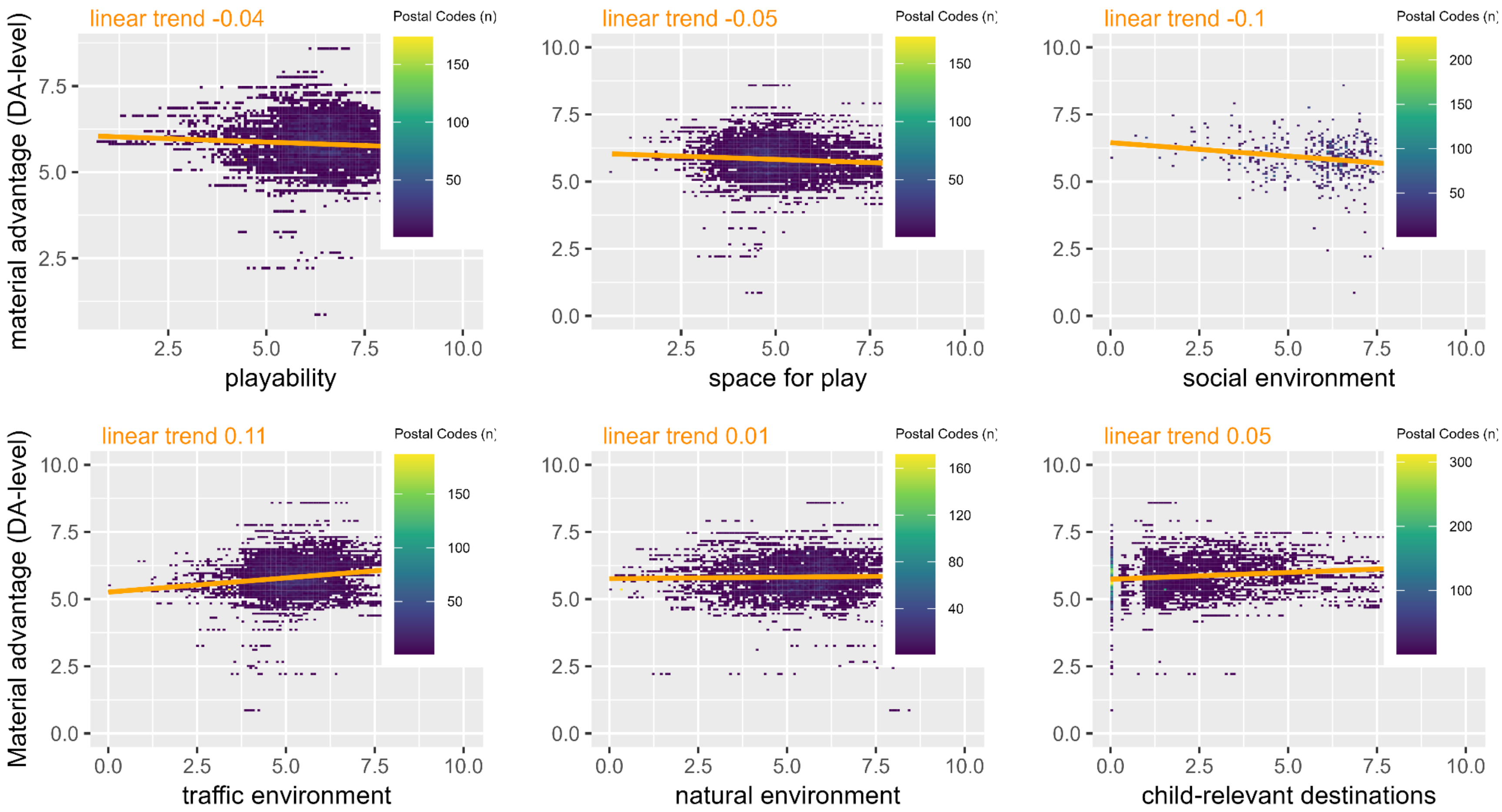

Figure D.47. Victoria density plots and linear trendlines for relationship between postal code-level playability, domains and material advantage.

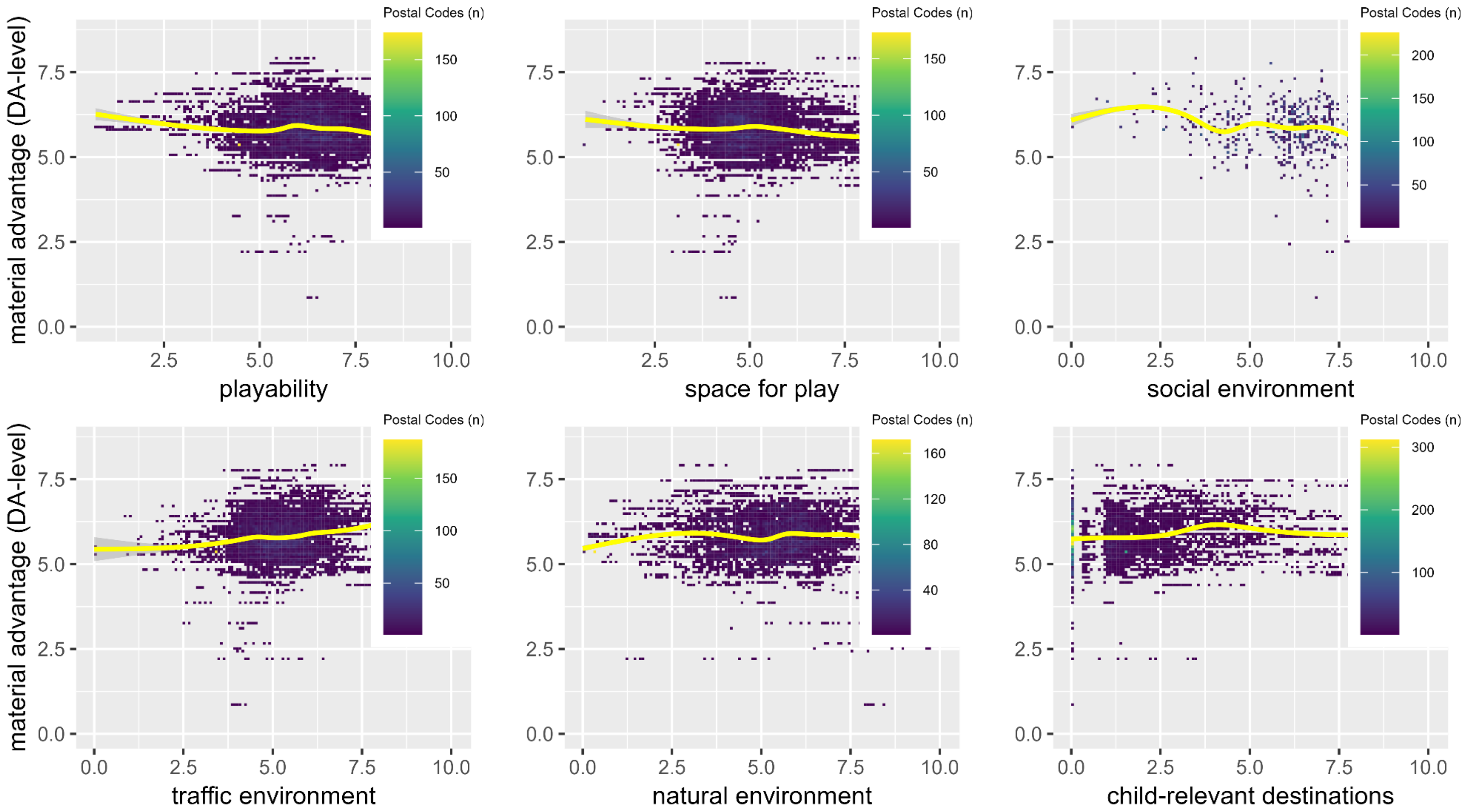

*Generalized additive models can be used to obtain a smoothed response function for the relationship between two variables. In this instance, a piecewise cubic function (spline) is used to approximate the relationship between playability, domains and population density.

Figure D.48. Victoria smoothed trendlines (generalized additive models*) for relationship between postal code-level playability, domains and material advantage.

References for Supplementary Materials

1. OpenStreetMap contributors. Planet dump retrieved from https://planet.osm.org.
2. DMTI Spatial Inc. CanMap Content Suite, v2020.3. (2020) doi:hdl:11272.1/AB2/FHWOBD.
3. Andrews, F. J., Stagnitti, K. & Robertson, N. Social play amongst preschool-aged children from an inner and an outer metropolitan suburb. *J. Soc. Incl.* **10**, 4–17 (2019).
4. Beattie, A. E. A Young Child's Perspectives on Outdoor Play: A Case Study from Vancouver, British Columbia. *Int. J. Early Child. Environ. Educ.* **3**, 38–53 (2015).
5. Removed for blinded peer review
6. Kimbro, R. T., Brooks-Gunn, J. & McLanahan, S. Young children in urban areas: Links among neighborhood characteristics, weight status, outdoor play, and television watching. *Soc. Sci. Med.* **72**, 668–676 (2011).
7. Benwell, M. C. Rethinking conceptualisations of adult-imposed restriction and children's experiences of autonomy in outdoor space. *Child. Geogr.* **11**, 28–43 (2013).
8. Gerlach, A. J., Jenkins, E. & Hodgson, K. Disrupting assumptions of risky play in the context of structural marginalization: A community engagement project in a Canadian inner-city neighbourhood. *Health Place* **55**, 80–86 (2019).
9. Marino, A. J. *et al.* Amount and environmental predictors of outdoor playtime at home and school: a cross-sectional analysis of a national sample of preschool-aged children attending Head Start. *Health Place* **18**, 1224–1230 (2012).

10. French, S. A., Sherwood, N. E., Mitchell, N. R. & Fan, Y. Park use is associated with less sedentary time among low-income parents and their preschool child: The NET-Works study. *Prev. Med. Rep.* **5**, 7–12 (2017).
11. Flowers, E. P., Timperio, A., Hesketh, K. D. & Veitch, J. Examining the Features of Parks That Children Visit During Three Stages of Childhood. *Int. J. Environ. Res. Public. Health* **16**, (2019).
12. CANUE. Canadian Urban Environmental Health Research Consortium. https://canue.ca/.
13. Hinkley, T., Salmon, J., Okely, A. D., Crawford, D. & Hesketh, K. Influences on preschool children's physical activity: Exploration through focus groups. *Fam. Community Health* **34**, 39–50 (2011).
14. Abu-Ghazzeh, T. M. Children's use of the street as a playground in Abu-Nuseir, Jordan. *Enivronment Behav.* **30**, 799–831 (1998).
15. Lee, E. Y. *et al.* Systematic review of the correlates of outdoor play and time among children aged 3-12 years. *Int. J. Behav. Nutr. Phys. Act.* **18**, (2021).
16. Ergler, C. R., Freeman, C. & Guiney, T. Walking with preschool-aged children to explore their local wellbeing affordances. *Geogr. Res.* **59**, 118–135 (2021).
17. Phillips, R. Parent perspectives of the neighbourhood outdoor play spaces for their young child. (University of British Columbia, 2016).
18. McGlone, N. Pop-Up kids: exploring children's experience of temporary public space. *Aust. Plan.* **53**, 117–126 (2016).

19. Hunter, S., Leatherdale, S. T., Spence, J. C. & Carson, V. Perceived Relevance of Neighborhood Features for Encouraging Preschoolers' Active Play, Parents' Active Recreation, and Parent-Child Coactivity. *Can. J. Behav. Sci.-Rev. Can. Sci. Comport.* **54**, 249–255 (2022).

20. Islam, M. Z., Moore, R. & Cosco, N. Child-friendly, active, healthy neighborhoods: Physical characteristics and children's time outdoors. *Environ. Behav.* **48**, 711–736 (2016).

21. Statistics Canada. Social Environment Variables. Data from Statistics Canada 2016, Census of Population. (2016).

22. Aarts, M. J. *et al.* Outdoor play among children in relation to neighborhood characteristics: a cross-sectional neighborhood observation study. *Int. J. Behav. Nutr. Phys. Act.* **9**, (2012).

23. Xu, H., Wen, L. M., Hardy, L. L. & Rissel, C. Mothers' perceived neighbourhood environment and outdoor play of 2- to 3.5-year-old children: Findings from the healthy beginnings trial. *Int. J. Environ. Res. Public. Health* **14**, 1082 (2017).

24. Stut, G. A quantitative analysis of children's (aged 2-18 years) outdoor play in Amsterdam Nieuw-West: comparing playground types and local demographic. (VU University Amsterdam, 2013).

25. Goodway, J. D. & Smith, D. W. Keeping all children healthy: challenges to leading an active lifestyle for preschool children qualifying for at-risk programs. *Fam. Community Health* **28**, 142–155 (2005).

26. Penilla, C., Tschann, J. M., Sanchez-Vaznaugh, E. V., Flores, E. & Ozer, E. J. Obstacles to preventing obesity in children aged 2 to 5 years: Latino mothers' and fathers' experiences and perceptions of their urban environments. *Int. J. Behav. Nutr. Phys. Act.* **14**, 1–12 (2017).

27. Chen, C., Yuan, Z. & Zhu, H. Playing, parenting and family leisure in parks: exploring emotional geographies of families in Guangzhou Children?s Park, China. *Child. Geogr.* **18**, 463–476 (2020).

28. Stanton-Chapman, T. L. & Schmidt, E. L. How Do the Children Play? The Influence of Playground Type on Children’s Play Styles. *Front. Psychol.* **12**, (2021).

29. Cronin-de-Chavez, A., Islam, S. & McEachan, R. R. C. Not a level playing field: A qualitative study exploring structural, community and individual determinants of greenspace use amongst low-income multi-ethnic families. *Health Place* **56**, 118–126 (2019).

30. Allport, T. *et al.* ‘Like a life in a cage’: Understanding child play and social interaction in Somali refugee families in the UK. *Health Place* **56**, 191–201 (2019).

31. Sampson, R. J. How does community context matter? Social mechanisms and the explanation of crime rates. in *The Explanation of Crime* (eds. Wikström, P.-O. H. & Sampson, R. J.) 31–60 (Cambridge University Press, 2006). doi:10.1017/CBO9780511489341.003.

32. Parent, N., Guhn, M., Brussoni, M., Almas, A. & Oberle, E. Social determinants of playing outdoors in the neighbourhood: family characteristics, trust in neighbours and daily outdoor play in early childhood. *Can. J. Public Health.* (2020) doi:10.17269/s41997-020-00355-w.

33. Cho, Y. The association between residential mobility and adolescents’ health: The mediating role of neighborhood social cohesion. *J. Community Psychol.* **48**, 1469–1480 (2020).

34. Berger-Schmitt, R. Considering Social Cohesion in Quality of Life Assessments: Concept and Measurement. in *Assessing Quality of Life and Living Conditions to Guide National Policy* (eds. Hagerty, M. R., Vogel, J. & Møller, V.) vol. 11 403–428 (Kluwer Academic Publishers, Dordrecht, 2002).

35. Dwyer, G. M., Higgs, J., Hardy, L. L. & Baur, L. A. What do parents and preschool stafftell us about young children's physical activity: A qualitative study. *Int. J. Behav. Nutr. Phys. Act.* **5**, 1–11 (2008).

36. Vancouver Police Department. Crime Statistics. *Vancouver Police Department* https://vpd.ca/crime-statistics/.

37. Soltero, E., Cerin, E., Lee, R. & O'Connor, T. Associations Between Objective and Self-Report Measures of Traffic and Crime Safety in Latino Parents of Preschool Children. *J. Immigr. Minor. Health* **19**, 1109–1120 (2017).

38. Kruse, J., Kang, Y., Liu, Y.-N., Zhang, F. & Gao, S. Places for play: Understanding human perception of playability in cities using street view images and deep learning. *Comput. Environ. Urban Syst.* **90**, 101693 (2021).

39. Pampelon. Material and Social Deprivation Index | INSPQ. (2023).

40. Bassul, C., Corish, C. A. & Kearney, J. M. Associations between home environment, children's and parents' characteristics and children's TV screen time behavior. *Int. J. Environ. Res. Public. Health* **18**, 1–15 (2021).

41. Aarts, M. J., Wendel-Vos, W., Van Oers, H. A. M., Van De Goor, I. A. M. & Schuit, A. J. Environmental determinants of outdoor play in children: A large-scale cross-sectional study. *Am. J. Prev. Med.* **39**, 212–219 (2010).

42. Eckert, J. & Al-Habashna, A. *Traffic Volume Estimation from Traffic Camera Imagery: Toward Real-Time Traffic Data Streams*. (2022).

43. Caroli, M. *et al.* Physical activity and play in kindergarten age children. *Int. J. Pediatr. Obes.* **6**, 47–53 (2011).

44. Huntsinger, L. F. Part 2 - Transportation systems planning. in *Highway Engineering (Second Edition)* (eds. Findley, D. J. et al.) 17–81 (Butterworth-Heinemann, 2022). doi:10.1016/B978-0-12-822185-3.00008-X.

45. Ministry of Transportation and Infrastructure. *Traffic Reports User Documentation*. 1–61 (2019).

46. Ferenchak, N. N. & Marshall, W. E. Suppressed child pedestrian and bicycle trips as an indicator of safety: Adopting a proactive safety approach. *Transp. Res. Part Policy Pract.* **124**, 128–144 (2019).

47. Clark, S. N. *et al.* Space-time characterization of community noise and sound sources in Accra, Ghana. *Sci. Rep.* **11**, 11113 (2021).

48. Wang, J. *et al.* Nitrogen oxides (NO and NO2) pollution in the Accra metropolis: Spatiotemporal patterns and the role of meteorology. *Sci. Total Environ.* **803**, 149931 (2022).

49. City of Toronto. About the Road Classification System – City of Toronto. *Streets, Parking and Transportation* https://www.toronto.ca/services-payments/streets-parking-transportation/traffic-management/road-classification-system/about-the-road-classification-system/ (2022).

50. City of Ottowa. Ottawa Road Classifications and Rights-of-Way. *Planning, Development and Construction* https://ottawa.ca/en/planning-development-and-construction/official-plan-and-master-plans/official-plan/volume-1-official-plan/section-7-annexes/annex-1-road-classification-and-rights-way#1-0-classification-summary (2022).

51. US Department of Transportation. *Highway Functional Classification: Concepts, Criteria and Procedures*. 7–9 (2013).

52. Government of B.C. Overview of B.C. Highway Functional Classification. *Transportation Guidelines and Tools* 4 https://www2.gov.bc.ca/assets/gov/driving-and-transportation/transportation-infrastructure/planning/inventories/bc_numbered_hwy_functional_classes.pdf (2014).
53. Zhang, H. & Malczewski, J. Accuracy Evaluation of the Canadian OpenStreetMap Road Networks. *Int. J. Geospatial Environ. Res.* **5**, (2018).
54. OpenStreetMap. Open Street Map Wiki: feature key 'highway'. *Open Street Map Wiki* https://wiki.openstreetmap.org/w/index.php?title=Key:highway&oldid=2250122. (2022).
55. Biddulph, M. Radical streets? The impact of innovative street designs on liveability and activity in residential areas. *Urban Des. Int.* **17**, 178–205 (2012).
56. Lambert, A., Vlaar, J., Herrington, S. & Brussoni, M. What Is the Relationship between the Neighbourhood Built Environment and Time Spent in Outdoor Play? A Systematic Review. *Int. J. Environ. Res. Public. Health* **16**, 3840 (2019).
57. Pokorny, P., Jensen, J. K., Gross, F. & Pitera, K. Safety effects of traffic lane and shoulder widths on two-lane undivided rural roads: A matched case-control study from Norway. *Accid. Anal. Prev.* **144**, 105614 (2020).
58. Bellefleur, O. *Traffic Lane Width of 3.0 m in Urban Environments*. https://ccnpps-ncchpp.ca/docs/2014-Built-Environment-Traffic-Lane-Width-of-3m-in-Urban-Environments.pdf (2014).
59. Xue, H., Cheng, X., Jia, P. & Wang, Y. Road network intersection density and childhood obesity risk in the US: a national longitudinal study. *Public Health Elsevier* **178**, 31–37 (2020).

60. Loptson, K., Muhajarine, N. & Ridalls, T. Walkable for whom? examining the role of the built environment on the neighbourhood-based physical activity of children. *Can. J. Public Health.* **103**, S29 (2012).

61. Janssen, I. & King, N. Walkable school neighborhoods are not playable neighborhoods. *Health Place* **35**, 66–69 (2015).

62. Buck, C. *et al.* Urban Moveability and physical activity in children: longitudinal results from the IDEFICS and I.Family cohort. *Int. J. Behav. Nutr. Phys. Act.* **16**, 1–13 (2019).

63. Wang, Q., Ma, J., Maehashi, A. & Kim, H. The associations between outdoor playtime, screen-viewing time, and environmental factors in chinese young children: The "eat, be active and sleep well" study. *Int. J. Environ. Res. Public. Health* **17**, 1–13 (2020).

64. do Carmo, A. S. *et al.* Influence of parental perceived environment on physical activity, TV viewing, active play and Body Mass Index among Portuguese children: A mediation analysis. *Am. J. Hum. Biol.* e23400 (2020) doi:10.1002/ajhb.23400.

65. Hosseini, M., Sevtsuk, A., Miranda, F., Cesar, R. M. & Silva, C. T. Mapping the walk: A scalable computer vision approach for generating sidewalk network datasets from aerial imagery. *Comput. Environ. Urban Syst.* **101**, 101950 (2023).

66. Ferster, C. *et al.* OSM Can-BICS. Zenodo https://doi.org/10.5281/zenodo.7444398 (2022).

67. Can-BICS National Data - Overview. https://www.arcgis.com/home/item.html?id=c6d2917c4a7d4fb4a8e7a615369b68d5.

68. Winters, M., Zanotto, M. & Butler, G. *The Canadian Bikeway Comfort and Safety (Can-BICS) Classification System: A Common Naming Convention for Cycling Infrastructure*. https://www.canada.ca/en/public-health/services/reports-publications/health-promotion-chronic-disease-prevention-canada-research-policy-practice/vol-40-no-9-2020/canbics-classification-system-naming-convention-cycling-infrastructure.html (2020).

69. City of Ottowa. Ottawa Road Classifications and Rights-of-Way. *Planning, Development and Construction* https://ottawa.ca/en/planning-development-and-construction/official-plan-and-master-plans/official-plan/volume-1-official-plan/section-7-annexes/annex-1-road-classification-and-rights-way#1-0-classification-summary (2022).

70. Overview of B . C . Highway Functional Classification. *Transportation Guidelines and Tools* 4 https://www2.gov.bc.ca/assets/gov/driving-and-transportation/transportation-infrastructure/planning/inventories/bc_numbered_hwy_functional_classes.pdf (2014).

71. Boyes, R. *et al.* Physical environment features that predict outdoor active play can be measured using Google Street View images. *Int. J. Health Geogr.* **22**, 26 (2023).

72. Grigsby-Toussaint, D. S., Chi, S.-H. & Fiese, B. H. Where they live, how they play: Neighborhood greenness and outdoor physical activity among preschoolers. *Int. J. Health Geogr.* **10**, (2011).

73. Kabisch, N. & Kraemer, R. Physical activity patterns in two differently characterised urban parks under conditions of summer heat. *Environ. Sci. Policy* **107**, 56–65 (2020).

74. Lynch, H., Moore, A., Edwards, C. & Horgan, L. Advancing play participation for all: The challenge of addressing play diversity and inclusion in community parks and playgrounds. *Br. J. Occup. Ther.* **83**, 107–117 (2020).

75. Mart, M., Simsar, A. & Uyanik, G. The Playground Perception of Syrian Refugee Children. *Child Indic. Res.* **15**, 349–372 (2022).

76. Oakley, J. *et al*. Backyard benefits? A cross-sectional study of yard size and greenness and children's physical activity and outdoor play. *BMC Public Health* **21**, 1–10 (2021).

77. Jarvis, I. *et al.* The influence of early-life residential exposure to different vegetation types and paved surfaces on early childhood development: A population-based birth cohort study. *Environ. Int.* **163**, (2022).

78. Ergler, C., Smith, K., Kotsanas, C. & Hutchinson, C. What Makes a Good City in Pre-schoolers' Eyes? Findings from Participatory Planning Projects in Australia and New Zealand. *J. Urban Des.* **20**, 461–478 (2015).

79. Ward, K. What's in a Dream? Natural Elements, Risk and Loose Parts in Children's Dream Playspace Drawings. *Australas. J. Early Child.* **43**, 34–42 (2018).

80. Hnatiuk, J. A., Dwyer, G., George, E. S. & Bennie, A. Co-participation in physical activity: Perspectives from Australian parents of pre-schoolers. *Health Promot. Int.* **35**, 1474–1483 (2020).

81. Phillips, R. Parent perspectives of the neighbourhood outdoor play spaces for their young child. (University of British Columbia, 2016).

82. Ganugi, G. & Prandini, R. Fostering social cohesion at the neighbourhood scale: the role of two Social Streets in Ferrara and Verona. *SN Soc. Sci.* **3**, 105 (2023).

83. Wickes, R., Zahnow, R., Corcoran, J. & Hipp, J. R. Neighbourhood social conduits and resident social cohesion. *Urban Stud.* **56**, 226–248 (2019).

84. Lu, C., Shen, T., Huang, G. & Corpeleijn, E. Environmental correlates of sedentary behaviors and physical activity in Chinese preschool children: a cross-sectional study. *J. Sport Health Sci.* **00**, (2020).

85. Padgham, M., Rudis, B., Lovelace, R. & Maelle, S. osmdata. *J. Open Source Softw.* **2**, (2017).

86. Pereira, R. H. M., Saraiva, M., Herszenhut, D., Braga, C. K. V., & Conway, M. W. r5r: Rapid Realistic Routing on Multimodal Transport Networks with R5 in R. *Findings* (2021).

87. R Core Team. R: A language and environment for statistical computing. R Foundation for Statistical Computing (2022).

88. Ferster, Colin. OSM_CAN_BICS_V2.

89. Ferster, C. *et al.* Developing a national dataset of bicycle infrastructure for Canada using open data sources. *Environ. Plan. B Urban Anal. City Sci.* 23998083231159905 (2023) doi:10.1177/23998083231159905.

90. Pereira, R. H. M., Saraiva, M., Herszenhut, D., Braga, C. K. V., & Conway, M. W. r5r: Rapid Realistic Routing on Multimodal Transport Networks with R5 in R. *Findings* (2021).

91. Pebesma, E. Simple Features for R: Standardized Support for Spatial Vector Data. *R J.* **10**, 439–446 (2018).

92. Setton, E. M. & Redivo, A. Canadian Building Footprints. Canadian Urban Environmental Research Consortium (2021).

93. Zhang, W., Liu, E. & Png, J. C. E. A framework for community noise modelling using machine learning methods. *Appl. Acoust.* **157**, 107033 (2020).

94. Gorelick, N. *et al.* Google Earth Engine: Planetary-scale geospatial analysis for everyone. *Remote Sens. Environ.* **202**, 18–27 (2017).

95. United States Geological Surveya. USGS Landsat 5 TM TOA Reflectancd (Orthorectified) 1984 - 2011.

96. United States Geological Surveyb. USGS Landsat 8 TOA Reflectance (Orthorectified) 2013-2017. USGS (2013).

97. United States Geological Surveyd. Landsat 8 Annual Greenest-Pixel TOA Reflectance Composite, 2013 to 2015. USGS.

98. DMTI Spatial Inc. CanMap Content Suite, [CanMap Postal Suite], v2015.3. Abacus Data Network (2015).

99. United States Geological Surveyc. Landsat 5 TM Annual Greenest-Pixel TOA Reflectance Composite, 1984 to 2012,. USGS.

100. Sexton, J. O. *et al.* Global, 30-m resolution continuous fields of tree cover: Landsat-based rescaling of MODIS vegetation continuous fields with lidar-based estimates of error. *Int. J. Digit. Earth* **6**, 427–448 (2013).

101. DMTI Spatial Inc. CanMap Postal Code Suite, v2020.3. Preprint at (2021).

102. Townsend, J. Global Forest Cover Change (GFCC) Tree Cover Multi-Year Global 30 m. NASA EOSDIS Land Processes Distributed Active Archive Center. https://doi.org/10.5067/MEaSUREs/GFCC/GFCC30TC.003.

103. Statistics Canada. Open Database of Educational Facilities. The Linkable Open Data Environment.

104. Statistics Canada. Open Database of Recreational and Sport Facilities (ODRSF). Linkable Open Data Environment6.

105. Institut National de Sante Publique du Quebec. Material and Social Deprivation Index | INSPQ. (2016).

106. Government of Canada, S. C. Dictionary, Census of Population, 2021 – Census subdivision (CSD). https://www12.statcan.gc.ca/census-recensement/2021/ref/dict/az/Definition-eng.cfm?ID=geo012 (2021).

107. Wijtzes, A. I. *et al.* Social inequalities in young children's sports participation and outdoor play. *Int. J. Behav. Nutr. Phys. Act.* **11**, (2014).

108. Pouliou, T. *et al.* Environmental influences on children's physical activity. *J Epidemiol Community Health* **69**, 77–85 (2015).

109. Kepper, M. M. *et al.* Using mixed methods to understand women's parenting practices related to their child's outdoor play and physical activity among families living in diverse neighborhood environments. *Health Place* **62**, 102292 (2020).

110. Brockman, R. *et al.* 'Get off the sofa and go and play': Family and socioeconomic influences on the physical activity of 10–11 year old children. *BMC Public Health* **9**, 253 (2009).

111. Glover, T. D., Todd, J. & Moyer, L. Neighborhood Walking and Social Connectedness. *Front. Sports Act. Living* **4**, (2022).

112. Hennig, C. B.C. condo association urges more tolerance after ban on outdoor play. *CBC News* (2018).

113. Government of Canada, S. C. Age (in single years), average age and median age and gender: Canada, provinces and territories, census divisions, census subdivisions and dissemination areas, 2021 census. (2022).

114. Government of Canada, S. C. Dictionary, Census of Population, 2021 - Low-income measures thresholds for private households of Canada, 2020. (2021).
